# Copper-catalyzed remote C–H arylation of polycyclic aromatic hydrocarbons (PAHs)

**DOI:** 10.3762/bjoc.16.49

**Published:** 2020-03-30

**Authors:** Anping Luo, Min Zhang, Zhangyi Fu, Jingbo Lan, Di Wu, Jingsong You

**Affiliations:** 1 Key Laboratory of Green Chemistry and Technology of Ministry of Education, College of Chemistry, Sichuan University, 29 Wangjiang Road, Chengdu 610064, P.R. China

**Keywords:** C–H arylation, nonprecious metal catalyst, copper catalysis, polycyclic aromatic hydrocarbons (PAHs), regioselectivity

## Abstract

The regioselective C–H arylation of substituted polycyclic aromatic hydrocarbons (PAHs) is a desired but challenging task. A copper-catalyzed C7–H arylation of 1-naphthamides has been developed by using aryliodonium salts as arylating reagents. This protocol does not need to use precious metal catalysts and tolerates wide variety of functional groups. Under standard conditions, the remote C–H arylation of other PAHs including phenanthrene-9-carboxamide, pyrene-1-carboxamide and fluoranthene-3-carboxamide has also accomplished, which provides an opportunity for the development of diverse organic optoelectronic materials.

## Introduction

Polycyclic aromatic hydrocarbons (PAHs) with rigid planar structure, such as naphthalene, phenanthrene, pyrene and their derivatives, can usually emit relatively strong fluorescence, and have been widely applied in many scientific areas including chemistry, biomedicine and materials science [1–6]. The arylation reaction of PAHs is an important strategy to further extend the π-conjugation length, which can effectively adjust the photophysical properties of molecules, thus having drawn much attention. Transition metal-catalyzed C–X/C–M cross-coupling reactions such as Suzuki and Stille couplings are the main approaches to achieve the arylation of PAHs [7–11]. However, the selective arylation of the C7-position of 1-naphthoic acid derivatives remains a challenging task due to the inaccessibility of the corresponding 7-halonaphthalene substrates [12].

**Scheme 1:**
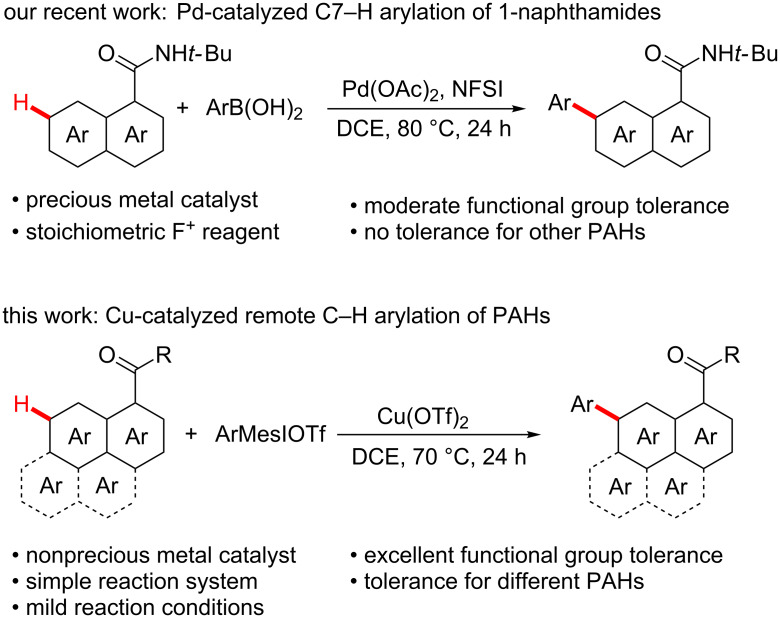
Direct C–H arylation of PAHs.

Recently, transition metal-catalyzed C–H bond functionalization has emerged as a powerful tool to construct various biaryl skeletons [13–17]. The direct C7−H arylation of 1-naphthoic acid derivatives is undoubtedly a more effective route for the synthesis of 7-arylnaphthalene derivatives. Although the transition metal-catalyzed C2−H and C8−H arylations of 1-naphthoic acid derivatives have been widely reported, the studies on their C7−H arylation remain rare [18–25]. Our group has recently reported F^+^ reagent-promoted Pd-catalyzed C7–H arylation of 1‑naphthamides, but this method still suffers from a few disadvantages (Scheme 1) [26]. First, the precious metal palladium is employed as a catalyst. Moreover, stoichiometric F^+^ reagent is needed to oxidize Pd(II) species to more electrophilic high-valent cationic Pd(IV). In addition, this protocol is not compatible with other PAHs except naphthalene, such as phenanthrene, pyrene and fluoranthene, and cannot tolerate some special functional groups, such as alkenyl and alkynyl groups.

As a component part of our ongoing research on direct C–H bond functionalization [20,27–29], we herein represent a copper-catalyzed remote C–H arylation of PAHs with aryliodonium salts as arylating reagents (Scheme 1). This protocol is compatible with different PAH substrates including 1-naphthamides, phenanthrene-9-carboxamide, pyrene-1-carboxamide and fluoranthene-3-carboxamide, which provides an opportunity for the development of diverse organic photoelectrical materials.

## Results and Discussion

Our investigation commenced with the reaction between *N*-(*tert*-butyl)-1-naphthamide (**1a**) and mesityl(phenyl)iodonium triflate (**2a**, for detailed optimization, see Table S1, Supporting Information File 1). Initially, the reaction was performed in 1,2-dichloroethane (DCE, 1 mL) at 80 °C for 24 h in the presence of Cu(OTf)_2_ (10 mol %) as a catalyst. The direct C7–H arylation product *N*-(*tert*-butyl)-7-phenyl-1-naphthamide (**3a**) was obtained in 79% yield (Table 1, entry 1). Gratifyingly, when the reaction temperature was reduced to 70 ºC, **3a** was obtained in 92% yield (Table 1, entry 2). The C7–H arylation could also occur with active copper powder as a catalyst (Table 1, entry 5). Other copper sources including CuO, CuCl and Cu(OAc)_2_ were also found to be effective catalysts in this reaction, albeit with slightly lower yields (Table 1, entries 6–8). The control experiment confirmed that this transformation did not occur in the absence of Cu catalyst (Table 1, entry 9). The screening of other solvents, such as dichloromethane (DCM), *ortho*-dichlorobenzene (ODCB), CHCl_3_ and PhCF_3_, indicated that DCE was still the best effective (Table 1, entries 10–13). Finally, the optimal reaction system was established, which composed of Cu(OTf)_2_ (10 mol %) in DCE (1.0 mL) at 70 °C under a nitrogen atmosphere for 24 hours.

**Table 1:** Optimization of reaction conditions.^a^

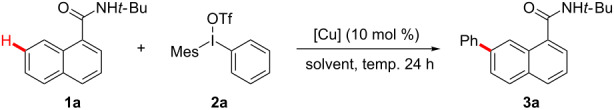				

Entry	Solvent	[Cu]	*T* (°C)	Yield (%)

1	DCE	Cu(OTf)_2_	80	79
2	DCE	Cu(OTf)_2_	70	92
3	DCE	Cu(OTf)_2_	90	53
4	DCE	Cu(OTf)_2_	60	41
5	DCE	Cu	70	54
6	DCE	CuO	70	81
7	DCE	CuCl	70	84
8	DCE	Cu(OAc)_2_	70	80
9	DCE	–	70	nd
10	DCM	Cu(OTf)_2_	70	38
11	ODCB	Cu(OTf)_2_	70	77
12	CHCl_3_	Cu(OTf)_2_	70	trace
13	PhCF_3_	Cu(OTf)_2_	70	trace

^a^Reaction conditions: **1a** (0.2 mmol, 1.0 equiv), **2a** (0.3 mmol, 1.5 equiv), [Cu] (10 mol %) and solvent (1 mL) under N_2_ for 24 h. Isolated yield. DCE = 1,2-dichloroethane. DCM = dichloromethane. ODCB = *ortho*-dichlorobenzene. nd: not detected.

**Scheme 2:**
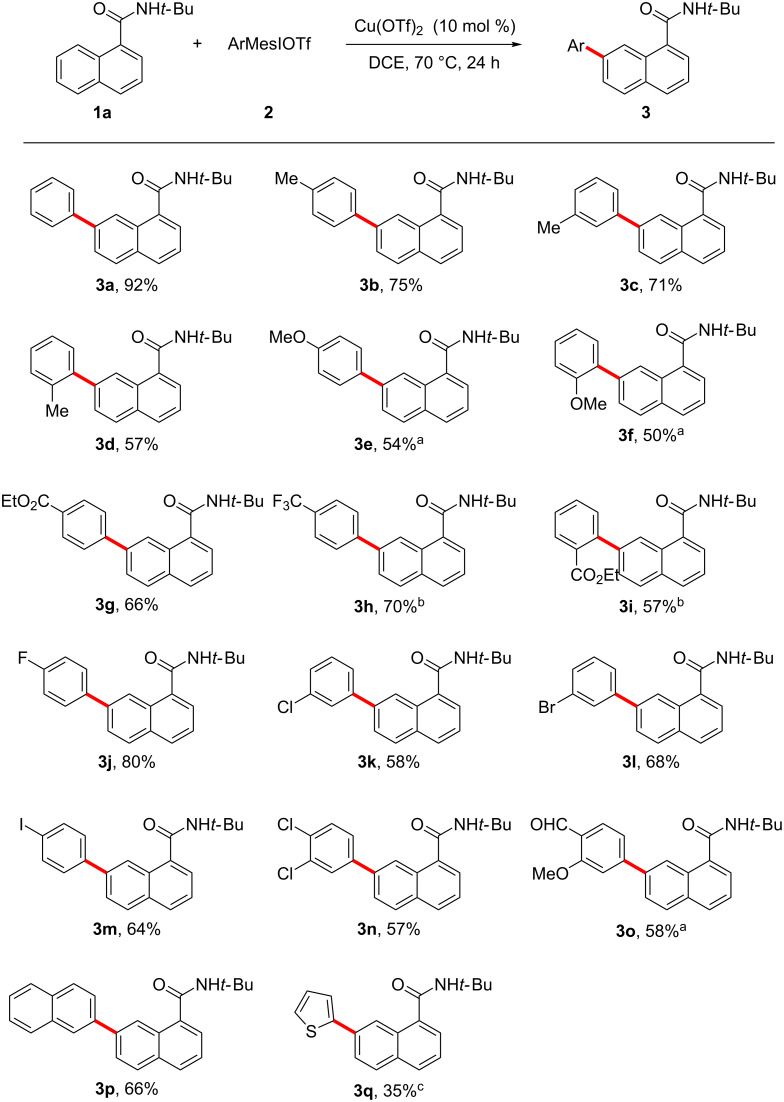
Scope of aryliodonium salts. Reaction conditions: **1a** (0.2 mmol), **2** (0.3 mmol) in DCE (1 mL) at 70 °C under N_2_ for 24 h. Isolated yield. ^a^60 °C. ^b^80 °C. ^c^50 °C.

**Scheme 3:**
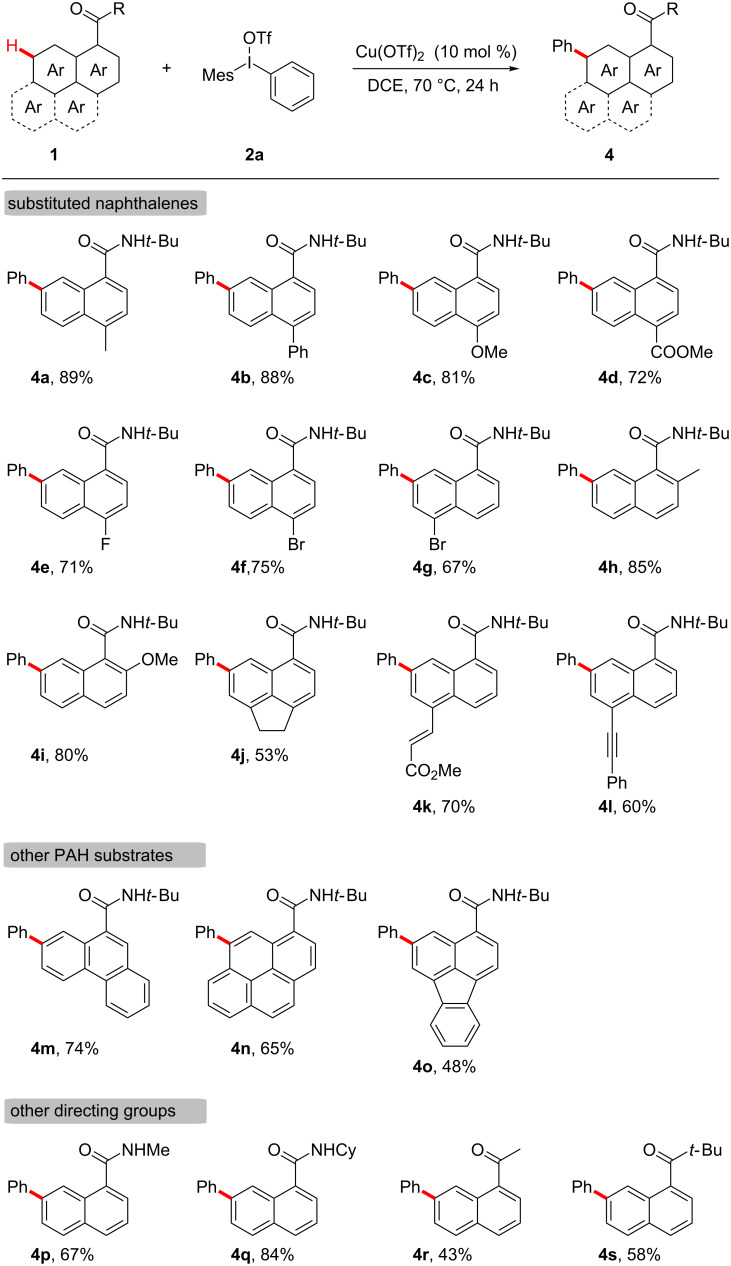
Scope of PAHs. Reaction conditions: **1** (0.2 mmol), **2a** (0.3 mmol) in DCE (1 mL) at 70 °C under N_2_ for 24 h. Isolated yield. DCE = 1,2-dichloroethane.

With the optimal conditions in hand, we first examined the scope of aryliodonium salts. We were very pleased to find that a range of aryliodonium salts could be employed as arylating reagents, affording 7-arylated 1-naphthamides (**3a**–**q**) in moderate to excellent yields (Scheme 2). This protocol tolerated a wide variety of functional groups, including electron-donating methyl and methoxy groups, as well as electron-withdrawing ester, trifluoromethyl, fluoro, chloro, bromo, iodo and formyl groups. The arylating reactivity of aryliodonium salts with various substituents varied greatly due to the different electronic effects and steric hindrances. Arylating reagents with *ortho*-substituents led to slightly reduced yields (Scheme 2, **3f** and **3i**). Aryliodonium salts containing halogen substituents, especially bromo and iodo atoms, could afford the desired products in moderate to good yields (Scheme 2, **3j**–**n**), making it possible to introduce useful functional groups into the products through the further transformation of corresponding aryl halides. 2-Naphthyliodonium salt could react smoothly with **1a** to provide **3p** in 66% yield (Scheme 2, **3p**). Moreover, thiophen-2-yliodonium salt could be tolerated, albeit with a lower yield (Scheme 2, **3q**).

**Scheme 4:**
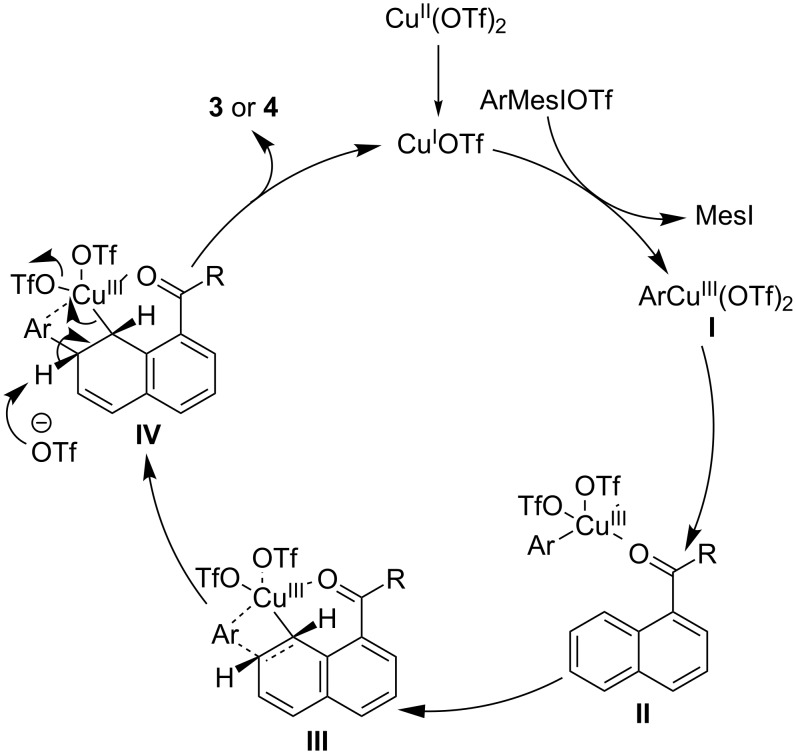
Proposed catalytic cycle.

We next examined the scope of various naphthalene substrates (Scheme 3, **4a**–**l**). The electronic effect of C4-substituents on *N*-(*tert*-butyl)-1-naphthamide was not obvious. The 4-substituted 1-naphthamide substrates, whether with electron-donating methyl and methoxy groups, or with electron-withdrawing phenyl, ester, fluoro and bromo groups, gave the corresponding products in good to excellent yields (Scheme 3, **4a**–**f**). Substrates with C2-substituents also exhibited excellent reactivity, providing the desired products **4h** and **4i** in 85% and 80% yields, respectively (Scheme 3, **4h** and **4i**). Notably, 1-naphthamides with alkenyl (**1l**) and alkynyl (**1m**) groups were also suitable substrates for this direct C7−H arylation, affording **4k** and **4l** in good yields (Scheme 3, **4k** and **4l**). Furthermore, this Cu-catalyzed direct C−H arylation could tolerate other PAH substrates. The regioselective arylation of PAHs is challenging, and so far, there are no examples on the selective remote C–H arylation of phenanthrene-9-carboxamide, pyrene-1-carboxamide and fluoranthene-3-carboxamide. Gratifyingly, the remote C–H arylation of these PAH substrates occurred smoothly, giving the corresponding arylation products in moderate to good yields (Scheme 3, **4m**–**o**).

This catalytic system was also compatible with substrates bearing other directing groups except *tert*-butylaminocarbonyl (Scheme 3, **4p**–**s**). When employing methylaminocarbonyl and cyclohexylaminocarbonyl as directing groups, the 7-arylation products of naphthalene rings were obtained in good yields (Scheme 3, **4p** and **4q**). A keto carbonyl group was also proved to be a suitable directing group, affording the corresponding arylation products in moderate yields (Scheme 3, **4r** and **4s**).

Considering that Cu(0), Cu(I) and Cu(II) all could catalyze this C−H arylation reaction and referring to previous research results [30–32], a Cu(I)/Cu(III) catalytic cycle was proposed (Scheme 4). First, Cu(I) is formed by the reduction or disproportionation of Cu(II). Then, aryliodonium salt oxidizes Cu(I) to highly electrophilic Cu(III)–aryl intermediate **I**. The coordination of the carbonyl oxygen to **I** gives intermediate **II**, which undergoes an aryl-transfer reaction via a Heck-like four-membered-ring transition state **III** to form the intermediate **IV** with Cu(III) and aryl group added at the C8- and C7-positions of the naphthalene ring, respectively. Finally, the breakdown of the C8–Cu bond delivers Cu(I), meantime, the OTf anion takes away the proton from the C7-position, affording the desired product **3** or **4**.

**Figure 1:**
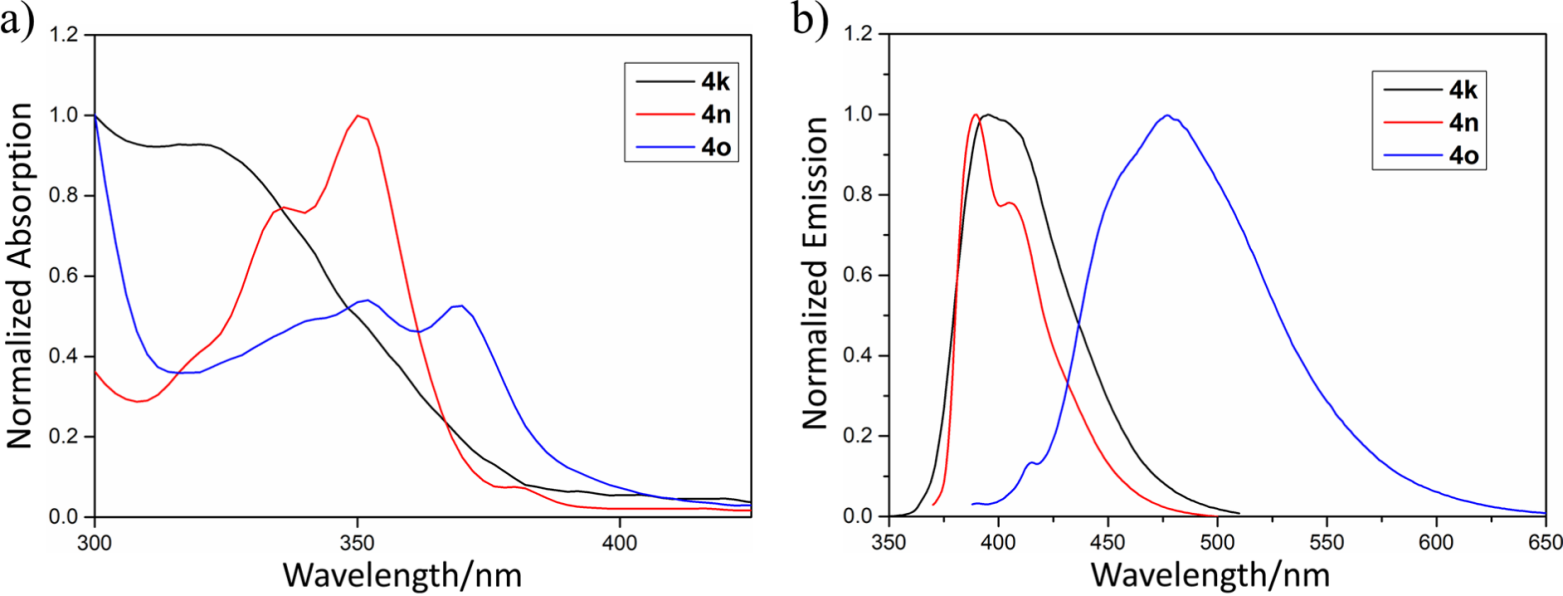
a) UV-visible absorption spectra of **4k**, **4n** and **4o** in toluene (1 × 10^−5^ mol/L). b) Emission spectra of **4k**, **4n** and **4o** in toluene (1 × 10^−5^ mol/L).

Subsequently, the photophysical properties of the arylation products **4k**, **4n** and **4o** were investigated (Figure 1). Their absorption bands cover from 300 nm to 400 nm, which corresponds to π−π* electron transition (Figure 1a and Table S2, Supporting Information File 1). The measurement of emission spectra demonstrates that **4k** and **4n** emit violet fluorescence with emission maxima at 395 nm and 390 nm, respectively, while **4o** exhibits a sky-blue emission with an emission maximum at 477 nm (Figure 1b and Table S2, Supporting Information File 1).

## Conclusion

In summary, we have developed a highly efficient strategy to accomplish the direct C7−H arylation of 1-naphthamides by the usage of Cu(II) as a catalyst and aryliodonium salts as arylating reagents, which features mild reaction conditions, excellent functional group tolerance, and moreover, does not need to use precious metal catalysts. This protocol is also compatible with other PAH substrates including phenanthrene-9-carboxamide, pyrene-1-carboxamide and fluoranthene-3-carboxamide, which provide an opportunity for the development of diverse organic photoelectrical materials.

## Supporting Information

File 1 Detailed experimental procedures, characterization data and copies of ^1^H and ^13^C NMR spectra of products.
