# Supplementary material for: Copper-catalyzed remote C–H arylation of polycyclic aromatic hydrocarbons (PAHs)
Source: Beilstein J Org Chem. 2020 Mar 30;16:530–6. doi: 10.3762/bjoc.16.49 (PMC7136547; doi:10.3762/bjoc.16.49)
Supplement: File 1 — Detailed experimental procedures, characterization data and copies of 1H and 13C NMR spectra of products. [file Beilstein_J_Org_Chem-16-530-s001.pdf]

BEILSTEIN JOURNAL OF ORGANIC CHEMISTRY

Supporting Information

for

Copper-catalyzed remote C–H arylation of polycyclic aromatic hydrocarbons (PAHs)

Anping Luo, Min Zhang, Zhangyi Fu, Jingbo Lan, Di Wu and Jingsong You

Beilstein J. Org. Chem. **2020,** *16,* 530–536. doi:10.3762/bjoc.16.49

Detailed experimental procedures, characterization data and copies of ^{1}H and ^{13}C NMR spectra of products

License and Terms: This is a supporting information file under the terms of the Creative Commons Attribution License (http://creativecommons.org/licenses/by/4.0). Please note that the reuse, redistribution and reproduction in particular requires that the authors and source are credited.
The license is subject to the *Beilstein Journal of Organic Chemistry* terms and conditions: (https://www.beilstein-journals.org/bjoc)

Table of Contents

I. General remarks S2

II. General procedure for the synthesis of starting materials S2

III. Optimization of the reaction conditions of remote C–H arylation S7

IV. General procedure for the synthesis of arylation products S9

V. Experimental data for the described substances S9

VI. Photophysical data S27

VII. References. S28

VIII. Copies of ^{1}H and ^{13}C NMR spectra S29

I. General remarks

NMR spectra were obtained on an Agilent 400-MR DD2 spectrometer. The ^{1}H NMR (400 MHz) chemical shifts were measured relative to $CDCl_3$ and DMSO-d_6 as the internal reference ($CDCl_3$: δ = 7.26 ppm; DMSO-d_6: δ = 2.50 ppm). The ^{13}C NMR (100 MHz) chemical shifts were given using $CDCl_3$ or DMSO-d_6 as the internal standard ($CDCl_3$: δ = 77.16 ppm; DMSO-d_6: δ = 39.52 ppm). High-resolution mass spectra (HRMS) were obtained with a Shimadzu LCMS-IT-TOF (ESI).

Unless otherwise noted, all reagents were obtained from commercial suppliers and used without further purification. $Cu(OTf)_2$ were purchased from Shanxi Kaida Chemical Engineering (China) Co., Ltd. Aryl iodides were purchased from Energy Chemical (China) Co., Ltd. Arylboronic acids and 1-(naphthalen-1-yl)ethanone (**1s**) were purchased from Energy Chemical (China) CO., Ltd. Solvents were dried with an innovative technology solvent purification system (model no.: PS-MD-5). All syntheses and manipulations were carried out under an air atmosphere unless specially noted.

Naphthamide derivatives **1a–d**, **1f–j**, **1q** and **1r** were synthesized according to literature procedures.[1] All diaryliodonium salts **2a–q** were synthesized according to literature procedures.[2-5]

II. General procedure for the synthesis of starting materials

General procedure A:

Preparation of methyl 4-(*tert*-butylcarbamoyl)-1-naphthoate (1e)[6]

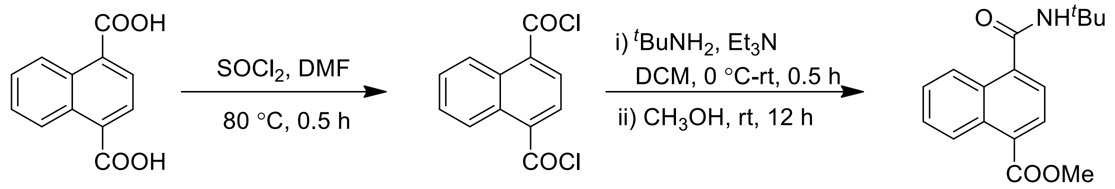

A 25 mL round bottom flask was charged with a magnetic stir bar, the corresponding naphthalene-1,4-dicarboxylic acid (5 mmol), DMF (*N*,*N*-dimethylformamide, 2 drops) and $SOCl_2$ (5.0 mL). Then the reaction solution was stirred at 80 °C to become clear for 0.5 h. The mixture was concentrated in vacuo (Bright yellow solid powder). Evaporation residue was dissolved in CH_2Cl_2 (DCM, 10 mL). The mixture of *tert*-butylamine (1.1 equiv) and Et_3N (3 equiv) was added drop by drop at 0 °C. The reaction

mixture was then stirred at room temperature for 0.5 h. Then 2 mL CH_3OH was added to the mixture. After addition, the solution was stirred at room temperature for 12 h. Then the reaction mixture was quenched with water and extracted with DCM. The organic layer was dried over Mg_2SO_4 and concentrated in vacuo. The residue was purified by column chromatography on silica gel (PE (petroleum ether)/EA (ethyl acetate) = 8/1, v/v) to yield **1e** (641.2 mg, 45%) as a white solid. ^{1}H NMR (400 MHz, $CDCl_3$): δ = 1.54 (s, 9H), 4.01 (s, 3H), 5.82 (bs, 1H), 7.52 (d, J = 7.6 Hz, 1H), 7.57-7.66 (m, 2H), 8.09 (d, J = 7.2 Hz, 1H), 8.21-8.24 (m, 1H), 8.88 (dd, J = 8.0, 1.3 Hz, 1H) ppm. ^{13}C NMR (100 MHz, $CDCl_3$): δ = 29.0, 52.51, 52.53, 122.9, 125.8, 126.1, 127.3, 128.2, 128.8, 129.1, 130.5, 131.6, 140.6, 167.7, 168.7 ppm. HRMS (ESI): calcd for $C_{17}H_{19}NNaO_3$ $[M+Na]^+$ 308.1257, found 308.1263. The analytical data matched those reported in the literature.[6]

General procedure B:

Preparation of (*E*)-methyl 3-(5-(*tert*-butylcarbamoyl)naphthalen-1-yl)acrylate (1l)

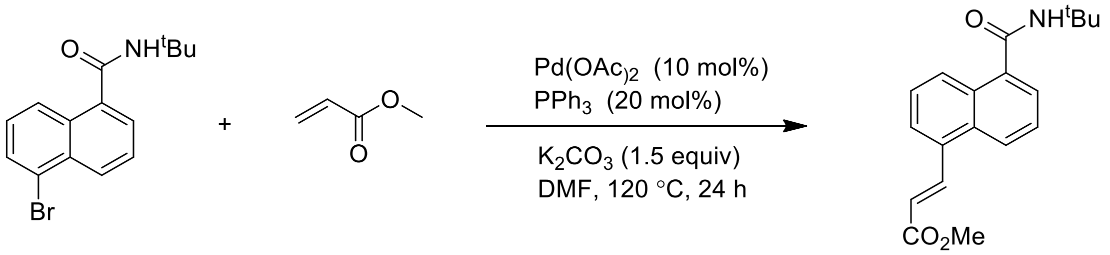

A Schlenk tube with a magnetic stir bar was charged with the corresponding 5-bromo-*N*-(*tert*-butyl)-1-naphthamide (0.6 mmol), methyl acrylate (0.9 mmol, 1.5 equiv), $Pd(OAc)_2$ (0.06 mmol, 10 mol %), PPh_3 (0.12 mmol, 20 mol %), K_2CO_3 (0.9 mmol, 1.5 equiv) and DMF (3.6 mL) under a N_2 atmosphere. Then the reaction solution was stirred at 120 °C for 24 h, and the reaction mixture was cooled to room temperature, and diluted with 3 mL of DCM. The mixture was filtered through a celite pad and washed with 10–20 mL of DCM. The filtrate was concentrated and the residue was purified by column chromatography on silica gel (PE/EA = 5/1, v/v) yield **1l** (140.6 mg, 81%) as a white solid. ^{1}H NMR (400 MHz, $CDCl_3$): δ = 1.54 (s, 9H), 3.85 (s, 3H), 5.82 (bs, 1H), 6.51 (d, J = 16.0 Hz, 1H), 7.50-7.59 (m, 3H), 7.76 (d, J = 7.2 Hz, 1H), 8.23 (d, J = 8.4 Hz, 1H), 8.31 (d, J = 8.8 Hz, 1H), 8.50 (d, J = 15.6 Hz, 1H) ppm. ^{13}C NMR (100 MHz,

CDCl$_3$): δ = 29.0, 52.0, 52.3, 121.1, 124.8, 125.4, 125.6, 125.9, 126.6, 127.9, 130.4, 131.7, 132.2, 136.8, 141.9, 167.3, 169.1 ppm. HRMS (ESI): calcd for $C_{19}H_{22}NO_3$ $[M+Na]^+$ 312.1594, found 312.1598.

General procedure C:

Preparation of *N*-(*tert*-butyl)-5-(phenylethynyl)-1-naphthamide (1m)

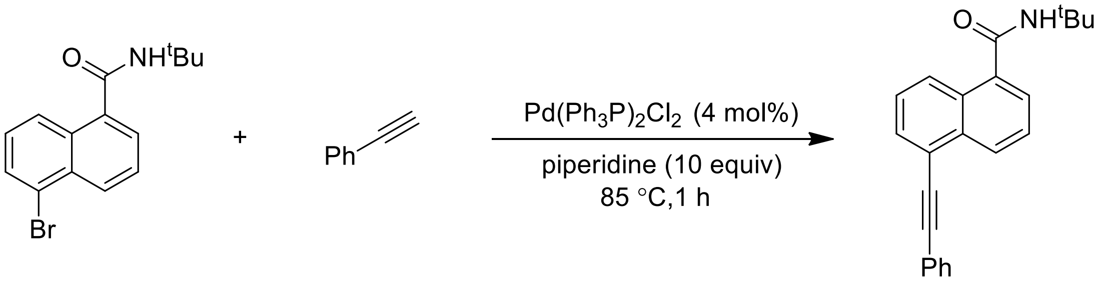

A Schlenk tube with a magnetic stir bar was charged with the corresponding 610 mg 5-bromo-*N*-(*tert*-butyl)-1-naphthamide (2 mmol), 0.2 mL phenylacetylene (2 mmol), 56 mg $Pd(PPh_3)_2Cl_2$ (0.08 mmol, 4 mol %), and 2 mL piperidine (20 mmol, 10 equiv) under a N_2 atmosphere. Then the reaction solution was stirred at 85 °C for 1 h, and the reaction mixture was cooled to room temperature, and diluted with 3 mL of DCM. The mixture was extracted with DCM (10 mL) and 1 M aqueous HCl (10 mL) for two times, saturated aqueous NaCl for two times and dried over anhydrous $MgSO_4$. The filtrate was concentrated, and the residue was purified by column chromatography on silica gel (PE/EA = 10/1, v/v) to yield **1m** (516.7 mg, 79%) as a white solid. ^{1}H NMR (400 MHz, CDCl$_3$): δ = 1.55 (s, 9H), 5.83 (bs, 1H), 7.36-7.43 (m, 3H), 7.50-7.61 (m, 3H), 7.63-7.67 (m, 2H), 7.79 (dd, J = 7.2, 1.0 Hz, 1H), 8.27 (d, J = 8.4 Hz, 1H), 8.49-8.54 (m, 1H) ppm. ^{13}C NMR (100 MHz, CDCl$_3$): δ = 29.1, 52.3, 87.5, 94.8, 121.4, 123.3, 125.1, 125.8, 126.3, 126.5, 128.4, 128.6, 128.7, 130.1, 131.05, 131.8, 133.6, 136.5, 169.1. ppm HRMS (ESI): calcd for $C_{23}H_{22}NO$ $[M+H]^+$ 328.1696, found 328.1702.

General procedure D:

Preparation of polycyclic aromatic hydrocarbon (PAH) substrates (1k, 1n–p)

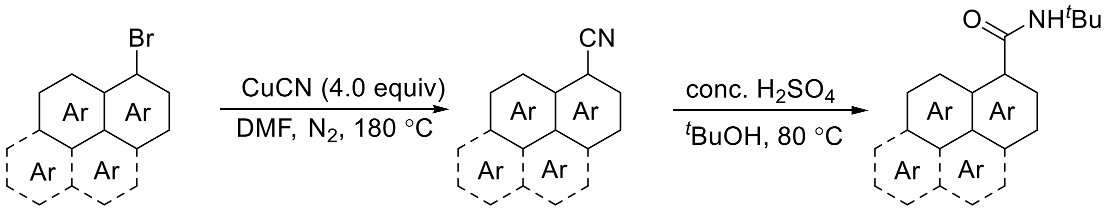

Polycyclic aromatic hydrocarbon (PAH) substrates were prepared by the following procedure adapted from the literature.[1]

i) Add 1-bromobenzene (2.0 mmol), CuCN (8.0 mmol) and DMF (2.0 mL) to the Schlenk bottle with a magnetic stir bar, and stir the reaction solution at 180 °C for 24 hours under a N_2 atmosphere. Then cooling the reaction mixture to room temperature, to this mixture, $FeCl_3$ (4.4g, 26.7 mmol) in 2 M aqueous HCl (8.0 mL) was added and stirred at 70 °C for 1 h. Then the mixture was extracted with DCM for four times, washed by 6 M aqueous HCl for two times, saturated aqueous Na_2CO_3 for two times, water for two times, and dried over anhydrous $MgSO_4$. The organic layer was dried and concentrated, and the resulting crude product 1-naphthonitrile could be used for next step without purification.

ii) A Schlenk tube with a magnetic stir bar was charged with corresponding 1-naphthonitrile (2.0 mmol), conc. H_2SO_4 (0.25 mL, 4.5 mmol) and *t*-BuOH (3.0 mL). The resulting mixture was stirred at 80 °C for 8–12 h (detected by TLC) and was cooled to room temperature. Then the reaction mixture was diluted with 8 mL DCM and extracted with brine (10 mL × 2). The organic layer was dried and concentrated, and the resulting residue was purified by column chromatography on silica gel (PE/EA = 20/1–10/1, v/v) to provide PAH substrates in 55–74% yields.

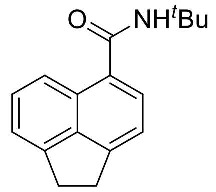

N-(tert-Butyl)-1,2-dihydroacenaphthylene-5-carboxamide (1k)

According to above procedure, compound **1k** was prepared from 5-bromo-1,2-dihydroacenaphthylene in 74% yield as a yellow solid. ^{1}H NMR (400 MHz, $CDCl_3$): δ = 1.53 (s, 9H), 3.36-3.44 (m, 4H), 5.84 (bs, 1H), 7.24 (d, J = 6.8 Hz, 1H), 7.32 (d, J = 7.2 Hz, 1H), 7.52 (m, 1H), 7.62 (d, J = 6.8 Hz, 1H), 8.06 (d, J = 8.4 Hz, 1H) ppm. ^{13}C NMR (100 MHz, $CDCl_3$): δ = 29.1, 30.4, 30.5, 52.0, 118.4, 120.0, 120.9, 127.4, 128.8, 129.1, 130.8, 139.6, 146.3, 149.1, 168.7 ppm. HRMS (ESI): calcd for $C_{17}H_{20}NO$ $[M+H]^+$ 254.1539, found 254.1548.

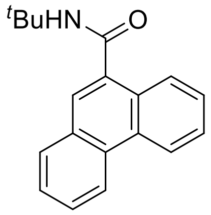

***N*-(*tert*-Butyl)phenanthrene-9-carboxamide (1n)**

According to above procedure, compound **1n** was prepared from 9-bromophenanthrene in 70% yield as a white solid. ^{1}H NMR (400 MHz, $CDCl_3$): δ = 1.57 (s, 9H), 5.93 (bs, 1H), 7.59-7.72 (m, 4H), 7.80 (s, 1H), 7.89 (dd, J = 8.0, 1.2 Hz, 1H), 8.27-8.32 (m, 1H), 8.67 (d, J = 8.4 Hz, 1H), 8.69-8.72 (m, 1H). ppm ^{13}C NMR (100 MHz, $CDCl_3$): δ = 29.1, 52.3, 122.8, 123.0, 125.6, 126.3, 127.1, 127.2, 127.3, 127.8, 128.8, 129.1, 130.6, 130.7, 130.9, 135.0, 169.3 ppm. HRMS (ESI): calcd for $C_{19}H_{20}NO$ $[M+H]^+$ 278.1539, found 278.1536. The analytical data matched those reported in the literature.[7]

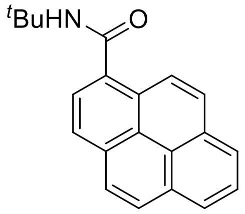

***N*-(*tert*-Butyl)pyrene-1-carboxamide (1o)**

According to above procedure, compound **1o** was prepared from 1-bromopyrene in 61% yield as a white solid. ^{1}H NMR (400 MHz, $CDCl_3$): δ = 1.61 (s, 9H), 5.96 (bs, 1H), 8.02-8.07 (m, 3H), 8.09-8.16 (m, 3H), 8.22 (d, J = 7.2 Hz, 2H), 8.53 (d, J = 9.2 Hz, 1H) ppm. ^{13}C NMR (100 MHz, $CDCl_3$): δ = 29.2, 52.4, 124.45, 124.49, 124.6, 124.9, 125.7, 125.8, 126.4, 127.3, 128.4, 128.5, 128.7, 130.9, 131.3, 132.3, 132.7, 169.7 ppm. HRMS (ESI): calcd for $C_{21}H_{19}NNaO$ $[M+Na]^+$ 324.1359, found 324.1363. The analytical data matched those reported in the literature.[8]

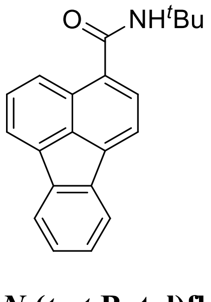

***N*-(*tert*-Butyl)fluoranthene-3-carboxamide (1p)**

According to above procedure, compound **1p** was prepared from 3-bromofluoranthene in 55% yield as a yellow solid. ^{1}H NMR (400 MHz, $CDCl_3$): δ = 1.56 (s, 9H), 5.95 (bs, 1H), 7.36-7.43 (m, 2H), 7.66 (dd, J = 8.4, 6.8 Hz, 1H), 7.77 (d, J = 7.2 Hz, 1H), 7.84-7.93 (m, 4H), 8.25-8.28 (m, 1H) ppm. ^{13}C NMR (100 MHz, $CDCl_3$): δ = 29.1, 52.3, 119.1, 120.7, 121.7, 122.0, 125.5, 127.2, 127.5, 127.9, 128.3, 129.1, 132.7, 134.8, 137.2, 138.7, 139.2, 140.1, 168.2 ppm. HRMS (ESI): calcd for $C_{21}H_{20}NO$ [M+Na]$^+$ 302.1539, found 302.1548.

General procedure E:

Preparation of 2,2-dimethyl-1-(naphthalen-1-yl)propan-1-one (1t)[9]

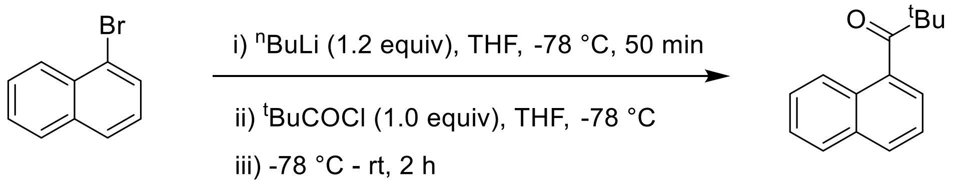

A Schlenk tube with a magnetic stir bar was charged with 1-bromonaphthalene (2.0 mmol) and THF (tetrahydrofuran, 4 mL) under a N_2 atmosphere. 1M of *n*-BuLi in hexane (2.5 mL) was added dropwise to the reaction solution at −78 °C, and then the reaction mixture was stirred at −78 °C for 50 min. Then 0.25 mL (2 mmol, 1.0 equiv) *t*-BuCOCl was added dropwise to the reaction mixture, warmed and stirred at room temperature for 2 h. Finally, the reaction mixture was quenched with NH_4Cl (aq.), diluted with DCM and extracted with brine, the organic layer was dried and concentrated, the residue was purified by column chromatography on silica gel (PE/EA = 60/1, v/v) yield **1t** (377.5 mg, 89%) as a white solid. ^{1}H NMR (400 MHz, $CDCl_3$): δ = 1.32 (s, 9H), 7.35 (dd, J = 7.2, 1.2 Hz, 1H), 7.43-7.52 (m, 3H), 7.59-7.64 (m, 1H), 7.84-7.88 (m, 2H) ppm. ^{13}C NMR (100 MHz, $CDCl_3$): δ = 27.4, 45.7, 122.4, 124.4, 125.6, 126.3, 126.9, 128.6, 129.1, 130.0, 133.7, 139.0, 214.8 ppm. HRMS (ESI): calcd for $C_{15}H_{16}NaO$ [M+Na]$^+$ 235.1093, found 235.1095. The analytical data matched those reported in the literature.[9]

III. Optimization of the reaction conditions of remote C–H arylation

An oven-dried Schlenk test tube with a magnetic stirring bar was charged with *N*-(*tert*-

butyl)-1-naphthamide **1a** (0.2 mmol, 1.0 equiv), mesityl(phenyl)iodonium triflate **2a** (0.3 mmol, 1.5 equiv), [Cu] catalyst (10 mol %), and solvent (1 mL) under a N_2 atmosphere. The mixture was stirred at the designed temperature for 24 h. After the reaction was cooled down to ambient temperature, it was diluted with 3 mL of CH_2Cl_2, filtered through a celite pad, and then washed with 15–20 mL of CH_2Cl_2. The combined organic phase was concentrated under reduced pressure and the residue was purified by column chromatography on silica gel (PE/THF = 20/1, v/v) to provide the desired product **3a**.

Table S1. Optimization of the arylation reaction of 1a and 2a[a]

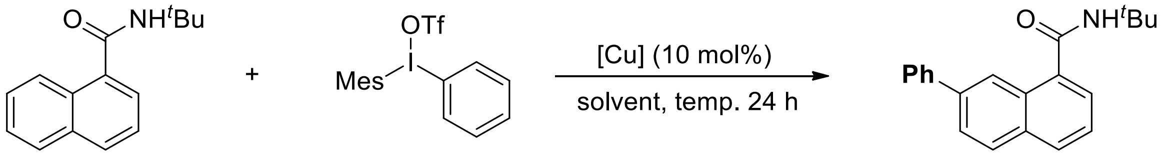

1a 2a 3a

Entry	Solvent	[Cu] (10 mol%)	T (°C)	Yield (%)[b]
1	DCE	$Cu(OTf)_2$	80	79
2	DCE	$Cu(OTf)_2$	70	92
3	DCE	$Cu(OTf)_2$	90	53
4	DCE	$Cu(OTf)_2$	60	41
5	DCE	$Cu(OTf)_2$	50	24
6	DCE	$Cu(OTf)_2$	100	35
7	DCE	-	70	nd
8	DCE	Cu	70	64
9	DCE	CuO	70	81
10	DCE	CuCl	70	84
11	DCE	$CuCl_2$	70	82
12	DCE	CuBr	70	81
13	DCE	$Cu(OAc)_2$	70	80
14	DCE	CuTc	70	62
15	DCM	$Cu(OTf)_2$	70	38
16	ODCB	$Cu(OTf)_2$	70	77
17	Toluene	$Cu(OTf)_2$	70	nd
18	MeOH	$Cu(OTf)_2$	70	nd

19	DMF	$Cu(OTf)_2$	70	nd
20	$CHCl_3$	$Cu(OTf)_2$	70	trace
21	$PhCF_3$	$Cu(OTf)_2$	70	trace

[a]Reaction conditions: **1a** (0.2 mmol, 1.0 equiv), **2a** (0.3 mmol, 1.5 equiv), [Cu] (10 mol%) and solvent (1 mL) under a N_2 atmosphere for 24 h. [b]Isolated yield. DCE = 1,2-Dichloroethane. DCM = Dichloromethane. ODCB= 1,2-Dichlorobenzene. MeOH = Methyl alcohol. DMF = *N,N*-Dimethylformamide. nd: not detected.

IV. General procedure for the synthesis of arylation products

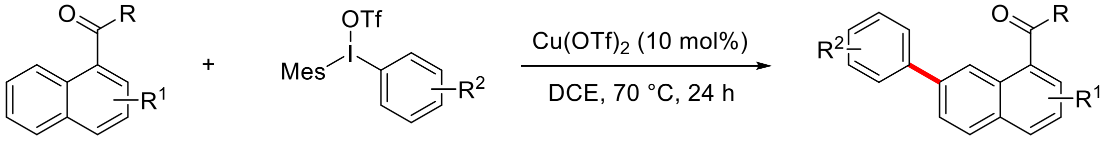

1 **2** **3 or 4**

An oven-dried Schlenk tube with a magnetic stir bar was charged with 1-naphthoic acid derivative **1** (0.2 mmol, 1.0 equiv), diaryliodonium salts **2** (0.3 mmol, 1.5 equiv), $Cu(OTf)_2$ (7.2 mg, 10 mol %) and DCE (1 mL) under a N_2 atmosphere. The mixture was stirred at 50–80 °C for 24 h. After the reaction was cooled down to ambient temperature, it was diluted with 3 mL of CH_2Cl_2. The solution was filtered through a celite pad and washed with 15–20 mL of CH_2Cl_2. The filtrate was concentrated under reduced pressure and the residue was purified by column chromatography on silica gel to provide the corresponding products **3** and **4**.

V. Experimental data for the described substances

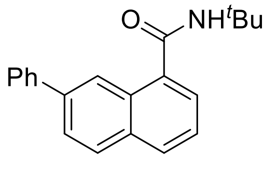

***N*-(*tert*-Butyl)-7-phenyl-1-naphthamide (3a)**

Following the general procedure, the reaction of *N*-(*tert*-butyl)-1-naphthamide (**1a**) (45.4 mg, 0.20 mmol), mesityl(phenyl)iodonium triflate (**2a**) (141.3 mg, 0.30 mmol), $Cu(OTf)_2$ (7.2 mg, 10 mol %), in DCE (1 mL) at 70 °C. After 24 h, purification by column chromatography on silica gel (PE/THF = 20/1, v/v) yields **3a** (55.8 mg, 92%)

as colorless oil. ^{1}H NMR (400 MHz, CDCl$_3$): δ = 1.55 (s, 9H), 5.87 (s, 1H), 7.37-7.41 (m, 1H), 7.42-7.46 (m, 1H), 7.46-7.52 (m, 2H), 7.58 (dd, J = 7.0, 1.1 Hz, 1H), 7.71-7.74 (m, 2H), 7.79 (dd, J = 8.4, 1.8 Hz, 1H), 7.89-7.94 (m, 2H), 8.51 (t, J = 0.8 Hz,1H) ppm. ^{13}C NMR (100 MHz, CDCl$_3$): δ = 29.1, 52.2, 123.4, 124.9, 125.0, 126.2, 127.63, 127.64, 128.9, 129.0, 129.9, 130.5, 132.9, 136.2, 139.7, 141.1, 169.3 ppm. The analytical data matched those reported in the literature.[1]

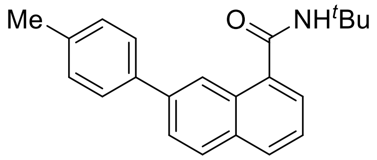

***N*-(*tert*-Butyl)-7-(*p*-tolyl)-1-naphthamide (3b)**

Following the general procedure, the reaction of *N*-(*tert*-butyl)-1-naphthamide (**1a**) (45.4 mg, 0.20 mmol), mesityl(*p*-tolyl)iodonium triflate (**2b**) (145.8 mg, 0.30 mmol), Cu(OTf)$_2$ (7.2 mg, 10 mol %), in DCE (1 mL) at 70 °C. After 24 h, purification by column chromatography on silica gel (PE/THF = 20/1, v/v) yields **3b** (47.6 mg, 75%) as colorless oil. ^{1}H NMR (400 MHz, CDCl$_3$): δ = 1.55 (s, 9H), 2.42 (s, 3H), 5.84 (s, 1H), 7.30 (d, J = 8.0 Hz, 2H), 7.4-7.45 (m, 1H), 7.57 (dd, J = 7.2, 1.2 Hz, 1H), 7.61-7.65 (m, 2H), 7.77 (dd, J = 8.4, 1.6 Hz, 1H), 7.90 (t, J = 8.4 Hz, 2H), 8.48 (s, 1H) ppm. ^{13}C NMR (100 MHz, CDCl$_3$): δ = 21.3, 29.1, 52.2, 123.0, 124.8, 125.0, 126.1, 127.5, 128.9, 129.8, 129.9, 130.5, 132.8, 136.2, 137.5, 138.2, 139.6, 169.3 ppm. The analytical data matched those reported in the literature.[1]

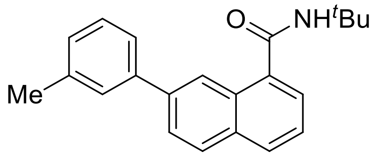

***N*-(*tert*-Butyl)-7-(*m*-tolyl)-1-naphthamide (3c)**

Following the general procedure, the reaction of *N*-(*tert*-butyl)-1-naphthamide (**1a**) (45.4 mg, 0.20 mmol), mesityl(*m*-tolyl)iodonium triflate (**2c**) (145.8 mg, 0.30 mmol), Cu(OTf)$_2$ (7.2 mg, 10 mol %), in DCE (1 mL) at 70 °C. After 24 h, purification by column chromatography on silica gel (PE/THF = 20/1, v/v) yields **3c** (45.1 mg, 71%) as colorless oil. ^{1}H NMR (400 MHz, CDCl$_3$): δ = 1.55 (s, 9H), 2.45 (s, 3H), 5.85 (s, 1H), 7.21 (d, J = 7.6 Hz, 1H), 7.38 (t, J = 7.4 Hz, 1H), 7.41-7.46 (m, 1H), 7.53 (d, J =

8.4 Hz, 2H), 7.58 (d, J = 6.8 Hz, 1H), 7.78 (dd, J = 8.4, 1.6 Hz, 1H), 7.91 (t, J = 8.4 Hz, 2H), 8.48 (s, 1H) ppm. ^{13}C NMR (100 MHz, $CDCl_3$): δ = 21.7, 29.1, 52.22, 76.8, 77.2, 77.5, 123.3, 124.8, 126.3, 128.39, 128.41, 128.8, 129.0, 129.9, 130.5, 132.9, 136.3, 138.6, 139.8, 141.1, 169.3 ppm. The analytical data matched those reported in the literature.[1]

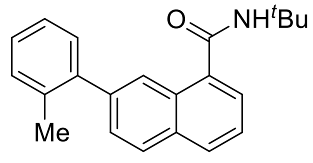

***N*-(*tert*-Butyl)-7-(*o*-tolyl)-1-naphthamide (3d)**

Following the general procedure, the reaction of *N*-(*tert*-butyl)-1-naphthamide (**1a**) (45.4 mg, 0.20 mmol), mesityl(*o*-tolyl)iodonium triflate (**2d**) (145.8 mg, 0.30 mmol), $Cu(OTf)_2$ (7.2 mg, 10 mol %), in DCE (1 mL) at 70 °C. After 24 h, purification by column chromatography on silica gel (PE/DCM/EA = 40/200/1, v/v/v) yields **3d** (36.2 mg, 57%) as colorless oil ^{1}H NMR (400 MHz, $CDCl_3$): δ =1.51 (s, 9H), 2.34 (s, 3H), 5.83 (bs, 1H), 7.27-7.35 (m, 4H), 7.43-7.47 (m, 1H), 7.52 (dd, J = 8.4, 1.6 Hz, 1H), 7.58 (dd, J = 7.2, 1.2 Hz, 1H), 7.91 (t, J = 8.8 Hz, 2H), 8.24 (s, 1H) ppm. ^{13}C NMR (100 MHz, $CDCl_3$): δ = 20.8, 29.1, 52.2, 124.8, 125.6, 126.0, 127.6, 128.0, 128.4, 130.0, 130.2, 130.6, 132.6, 135.7, 136.2, 140.6, 141.8, 169.2 ppm. The analytical data matched those reported in the literature.[1]

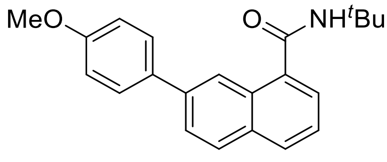

***N*-(*tert*-Butyl)-7-(4-methoxyphenyl)-1-naphthamide (3e)**

Following the general procedure, the reaction of *N*-(*tert*-butyl)-1-naphthamide (**1a**) (45.4 mg, 0.20 mmol), mesityl(4-methoxyphenyl)iodonium triflate (**2e**) (150.6 mg, 0.30 mmol), $Cu(OTf)_2$ (7.2 mg, 10 mol %), in DCE (1 mL) at 60 °C. After 24 h, purification by column chromatography on silica gel (PE/THF = 15/1, v/v) yields **3e** (36.2 mg, 54%) as colorless oil. ^{1}H NMR (400 MHz, $CDCl_3$): δ = 1.55 (s, 9H), 3.87 (s, 3H), 5.84 (bs, 1H), 7.00-7.05 (m, 2H), 7.39-7.43 (m, 1H), 7.56 (dd, J = 6.8, 1.2 Hz, 1H), 7.64-7.68 (m, 2H), 7.75 (dd, J = 8.4, 1.6 Hz, 1H), 7.89 (t, J = 7.6 Hz, 2H), 8.44 (t, J = 0.8 Hz, 1H)

ppm. ^{13}C NMR (100 MHz, $CDCl_3$): δ = 29.1, 52.2, 55.5, 114.5, 122.6, 124.6, 125.0, 126.0, 128.6, 128.9, 129.9, 130.5, 132.6, 133.6, 136.1, 139.3, 159.5, 169.3 ppm. HRMS (ESI): calcd for $C_{22}H_{24}NO_2$ $[M+H]^+$ 334.1802, found 334.1807.

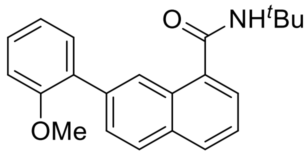

N-(*tert*-Butyl)-7-(2-methoxyphenyl)-1-naphthamide (3f)

Following the general procedure, the reaction of *N*-(*tert*-butyl)-1-naphthamide (**1a**) (45.4 mg, 0.20 mmol), mesityl(2-methoxyphenyl)iodonium triflate (**2f**) (150.6 mg, 0.30 mmol), $Cu(OTf)_2$ (7.2 mg, 10 mol %), in DCE (1 mL) at 60 °C.. After 24 h, purification by column chromatography on silica gel (PE/THF = 15/1, v/v) yields **3f** (33.4 mg, 50%) as colorless oil. ^1H NMR (400 MHz, $CDCl_3$): δ = 1.52 (s, 9H), 3.84 (s, 3H), 5.83 (bs, 1H), 7.03 (d, J = 8.0 Hz, 1H), 7.08 (td, J = 7.6, 0.8 Hz, 1H), 7.37 (td, J = 8.0, 1.6 Hz, 1H), 7.41-7.46 (m, 2H), 7.56 (dd, J = 7.2, 1.2 Hz, 1H), 7.74 (dd, J = 8.4, 1.6 Hz, 1H), 7.86-7.90 (m, 2H), 8.39 (s, 1H) ppm. ^{13}C NMR (100 MHz, $CDCl_3$): δ = 29.1, 52.1, 55.7, 111.4, 121.2, 124.7, 124.8, 125.6, 127.7, 128.7, 129.0, 129.8, 130.1, 130.7, 131.4, 132.7, 136.2, 137.4, 156.7, 169.3 ppm. HRMS (ESI): calcd for $C_{22}H_{24}NO_2$ $[M+H]^+$ 334.1802, found 334.1808.

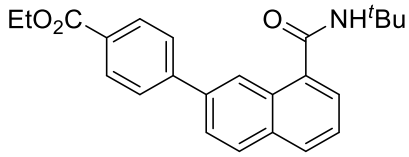

Ethyl 4-(8-(*tert*-butylcarbamoyl)naphthalen-2-yl)benzoate (3g)

Following the general procedure, the reaction of *N*-(*tert*-butyl)-1-naphthamide (**1a**) (45.4 mg, 0.20 mmol), (4-(ethoxycarbonyl)phenyl)(mesityl)iodonium triflate (**2g**) (163.2 mg, 0.30 mmol), $Cu(OTf)_2$ (7.2 mg, 10 mol %), in DCE (1 mL) at 70 °C. After 24 h, purification by column chromatography on silica gel (PE/THF = 10/1, v/v) yields **3g** (49.6 mg, 66%) as colorless oil. ^1H NMR (400 MHz, $CDCl_3$): δ = 1.42 (t, J = 7.2 Hz, 3H), 1.54 (s, 9H), 4.41 (q, J = 7.2 Hz, 2H), 5.90 (s, 1H), 7.43-7.48 (m, 1H), 7.58 (d, J = 6.9 Hz, 1H), 7.75-7.81 (m, 3H), 7.89-7.95 (m, 2H), 8.14 (d, J = 8.4 Hz, 2H), 8.54 (s, 1H) ppm. ^{13}C NMR (100 MHz, $CDCl_3$): δ = 14.5, 29.1, 52.3, 61.2, 76.8, 77.2, 77.45,

124.0, 125.1, 125.4, 125.9, 127.5, 129.1, 129.5, 130.0, 130.3, 130.4, 133.3, 136.3, 138.4, 145.4, 166.6, 169.1 ppm. The analytical data matched those reported in the literature.[1]

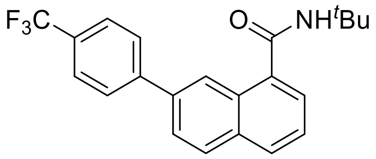

N-(*tert*-Butyl)-7-(4-(trifluoromethyl)phenyl)-1-naphthamide (3h)

Following the general procedure, the reaction of *N*-(*tert*-butyl)-1-naphthamide (**1a**) (45.4 mg, 0.20 mmol), mesityl(4-(trifluoromethyl)phenyl)iodonium triflate (**2h**) (162.0 mg, 0.30 mmol), $Cu(OTf)_2$ (7.2 mg, 10 mol %), in DCE (1 mL) at 80 °C. After 24 h, purification by column chromatography on silica gel (PE/THF = 10/1, v/v) yields **3h** (52.1 mg, 70%) as colorless oil. ^{1}H NMR (400 MHz, $CDCl_3$): δ = 1.55 (s, 9H), 5.86 (bs, 1H), 7.44-7.50 (m, 1H), 7.60 (dd, J = 7.2, 1.2 Hz, 1H), 7.72-7.78 (m, 3H), 7.82 (d, J = 8.0 Hz, 2H), 7.92 (d, J = 8.0 Hz, 1H), 7.96 (d, J = 8.4 Hz, 1H), 8.54 (s, 1H) ppm. ^{13}C NMR (100 MHz, $(CD_3)_2SO$): δ = 28.6, 51.1, 123.6, 124.4 (q, J_{CF} = 271 Hz), 125.2, 125.6, 125.8, 126.2 (q, J_{CF} = 3.7 Hz), 127.7, 128.1 (d, J_{CF} = 32 Hz), 129.0, 129.3, 129.9, 132.7, 136.49, 136.53, 144.3 (d, J_{CF} = 1.3 Hz), 168.3 ppm. HRMS (ESI): calcd for $C_{22}H_{21}F_3NO$ $[M+H]^+$ 372.1570, found 372.1572.

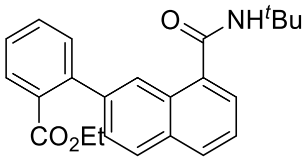

Ethyl 2-(8-*(tert*-butylcarbamoyl)naphthalen-2-yl)benzoate (3i)

Following the general procedure, the reaction of *N*-(*tert*-butyl)-1-naphthamide (**1a**) (45.4 mg, 0.20 mmol), (2-(ethoxycarbonyl)phenyl)(mesityl)iodonium triflate (**2i**) (163.2 mg, 0.30 mmol), $Cu(OTf)_2$ (7.2 mg, 10 mol %), in DCE (1 mL) at 80 °C. After 24 h, purification by column chromatography on silica gel (PE/THF = 10/1, v/v) yields **3i** (42.8 mg, 57%) as a white solid. ^{1}H NMR (400 MHz, $CDCl_3$): δ = 0.98 (t, J = 7.2 Hz, 3H), 1.51 (s, 9H), 4.10 (q, J = 7.2 Hz, 2H), 5.83 (bs, 1H), 7.42-7.50 (m, 4H), 7.57 (td, J = 7.6, 1.2 Hz, 2H), 7.85 (d, J = 8.4 Hz, 1H), 7.89-7.92 (m, 2H), 8.26 (t, J = 0.8 Hz 1H) ppm. ^{13}C NMR (100 MHz, $CDCl_3$): δ = 14.0, 29.1, 52.2, 61.1, 124.6, 124.9, 125.0, 127.5, 127.7, 127.9, 130.0, 130.1, 130.2, 131.1, 131.4, 131.6, 132.8, 136.1, 140.3,

142.7, 168.5, 169.2 ppm. HRMS (ESI): calcd for $C_{24}H_{25}NNaO_3$ $[M+Na]^+$ 398.1727, found 398.1731.

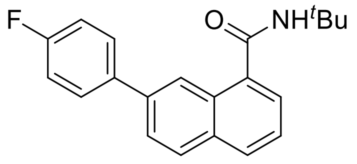

***N-(tert*-Butyl)-7-(4-fluorophenyl)-1-naphthamide (3j)**

Following the general procedure, the reaction of *N*-(*tert*-butyl)-1-naphthamide (**1a**) (45.4 mg, 0.20 mmol), (4-fluorophenyl)(mesityl)iodonium triflate (**2j**) (147.0 mg, 0.30 mmol), $Cu(OTf)_2$ (7.2 mg, 10 mol %), in DCE (1 mL) at 70 °C. After 24 h, purification by column chromatography on silica gel (PE/THF = 20/1, v/v) yields **3j** (51.4 mg, 80%) as colorless oil. ^{1}H NMR (400 MHz, $CDCl_3$): δ = 1.55 (s, 9H), 5.85 (s, 1H), 7.14-7.20 (m, 2H), 7.42-7.46 (m, 1H), 7.58 (dd, J = 7.2, 1.2 Hz, 1H), 7.65-7.70 (m, 2H), 7.73 (dd, J = 8.4, 2.0 Hz, 1H), 7.91 (t, J = 8.0 Hz, 2H), 8.45 (s, 1H) ppm. ^{13}C NMR (100 MHz, $CDCl_3$): δ = 29.1, 52.3, 115.9 ($J_{C\text{-}F}$ = 21 Hz), 123.3, 125.0 ($J_{C\text{-}F}$ = 10 Hz), 126.0, 129.0, 129.15, 129.23, 130.0, 130.5, 132.9, 136.1, 137.3, ($J_{C\text{-}F}$ = 3 Hz)，138.7, 162.8 ($J_{C\text{-}F}$ = 245 Hz), 169.2 ppm. The analytical data matched those reported in the literature.[1]

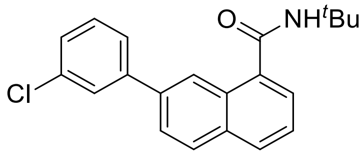

***N*-(*tert*-Butyl)-7-(3-chlorophenyl)-1-naphthamide (3k)**

Following the general procedure, the reaction of *N*-(*tert*-butyl)-1-naphthamide (**1a**) (45.4 mg, 0.20 mmol), (3-chlorophenyl)(mesityl)iodonium triflate (**2k**) (151.8 mg, 0.30 mmol), $Cu(OTf)_2$ (7.2 mg, 10 mol %), in DCE (1 mL) at 70 °C. After 24 h, purification by column chromatography on silica gel (PE/THF = 20/1, v/v) yields **3k** (39.1 mg, 58%) as colorless oil. ^{1}H NMR (400 MHz, $CDCl_3$): δ = 1.55 (s, 9H), 5.85 (bs, 1H), 7.35 (dt, J = 8.0, 1.4 Hz, 1H), 7.41 (t, J = 7.6 Hz, 1H), 7.45-7.48 (m, 1H), 7.58-7.61 (m, 2H), 7.69 (t, J = 2.0 Hz, 1H), 7.73 (dd, J = 8.4, 1.8 Hz, 1H), 7.92 (t, J = 9.2 Hz, 2H), 8.48 (s, 1H) ppm. ^{13}C NMR (100 MHz, $CDCl_3$): δ = 29.1, 52.3, 123.7, 125.2, 125.3, 125.8, 125.9, 127.65, 127.71, 129.1, 130.0, 130.3, 130.4, 133.2, 134.9, 136.3, 138.3, 143.0, 169.1 ppm. HRMS (ESI): calcd for $C_{21}H_{20}ClNNaO$ $[M+Na]^+$ 360.1126, 362.1096,

found 360.1128, 362.1097.

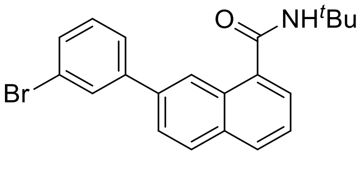

7-(3-Bromophenyl)-*N*-(*tert*-butyl)-1-naphthamide (3l)

Following the general procedure, the reaction of *N*-(*tert*-butyl)-1-naphthamide (**1a**) (45.4 mg, 0.20 mmol), (3-bromophenyl)(mesityl)iodonium triflate (**2l**) (165.3 mg, 0.30 mmol), $Cu(OTf)_2$ (7.2 mg, 10 mol %), in DCE (1 mL) at 70 °C. After 24 h, purification by column chromatography on silica gel (PE/THF = 20/1, v/v) yields **3l** (51.8 mg, 68%) as a white solid. ^{1}H NMR (400 MHz, $CDCl_3$): δ = 1.55 (s, 9H), 5.86 (bs, 1H), 7.35 (t, J = 8.0 Hz, 1H), 7.43-7.48 (m, 1H), 7.51 (ddd, J = 8.0, 2.0, 0.8 Hz, 1H), 7.59 (dd, J = 7.2, 1.2 Hz, 1H), 7.64 (ddd, J = 7.6, 1.6, 0.8 Hz, 1H), 7.73 (dd, J = 8.8, 2.0 Hz, 1H), 7.84 (t, J = 2.0 Hz, 1H), 7.92 (t, J = 9.2 Hz, 2H), 8.47 (t, J = 0.8 Hz, 1H) ppm. ^{13}C NMR (100 MHz, $CDCl_3$): δ = 29.1, 52.3, 123.1, 123.7, 125.2, 125.3, 125.9, 126.3, 129.1, 130.0, 130.4, 130.55, 130.57, 130.6, 133.2, 136.3, 138.2, 143.0, 169.1 ppm. HRMS (ESI): calcd for $C_{21}H_{20}BrNNaO$ [M+Na]$^+$ 404.0620, 406.0600, found 404.0623, 406.0596.

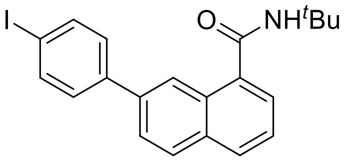

***N*-(*tert*-Butyl)-7-(4-iodophenyl)-1-naphthamide (3m)**

Following the general procedure, the reaction of *N*-(*tert*-butyl)-1-naphthamide (**1a**) (45.4 mg, 0.20 mmol), (4-iodophenyl)(mesityl)iodonium triflate (**2m**) (179.4 mg, 0.30 mmol), $Cu(OTf)_2$ (7.2 mg, 10 mol %), in DCE (1 mL) at 70 °C. After 24 h, purification by column chromatography on silica gel (PE/THF = 20/1, v/v) yields **3m** (54.9 mg, 64%) as a white solid. ^{1}H NMR (400 MHz, $CDCl_3$): δ = 1.54 (s, 9H), 5.85 (bs, 1H), 7.42-7.47 (m, 3H), 7.58 (dd, J = 7.2, 1.2 Hz, 1H), 7.72 (dd, J = 8.4, 2.0 Hz, 1H), 7.79-7.83 (m, 2H), 7.91 (t, J = 8.8 Hz, 2H), 8.47 (t, J = 0.8 Hz 1H) ppm. ^{13}C NMR (100 MHz, $CDCl_3$): δ = 29.1, 52.3, 93.5, 123.38, 125.1, 125.2, 125.7, 129.1, 129.4, 130.0, 130.5, 133.1, 136.2, 138.1, 138.5, 140.7, 169.2 ppm. HRMS (ESI): calcd for $C_{21}H_{20}INNaO$ [M+Na]$^+$ 452.0482, 453.0515, found 452.0489, 453.0520.

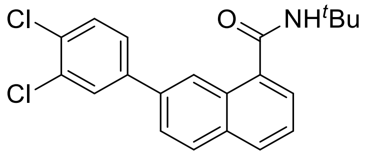

***N*-(*tert*-Butyl)-7-(3,4-dichlorophenyl)-1-naphthamide (3n)**

Following the general procedure, the reaction of *N*-(*tert*-butyl)-1-naphthamide (**1a**) (45.4 mg, 0.20 mmol), (3,4-dichlorophenyl)(mesityl)iodonium triflate (**2n**) (162.0 mg, 0.30 mmol), $Cu(OTf)_2$ (7.2 mg, 10 mol %), in DCE (1 mL) at 70 °C. After 24 h, purification by column chromatography on silica gel (PE/THF = 20/1, v/v) yields **3n** (42.3 mg, 57%) as a white solid. ^{1}H NMR (400 MHz, $CDCl_3$): δ = 1.55 (s, 9H), 5.87 (bs, 1H), 7.44-7.48 (m, 1H), 7.53 (d, J = 1.2 Hz, 2H), 7.59 (dd, J = 6.8, 1.2 Hz, 1H), 7.69 (dd, J = 8.4, 2.0 Hz, 1H), 7.77 (t, J = 1.2 Hz, 1H), 7.89-7.94 (m, 2H), 8.45-8.47 (m, 1H) ppm. ^{13}C NMR (100 MHz, $CDCl_3$): δ = 29.1, 52.3, 123.7, 125.3, 125.4, 125.5, 126.9, 129.3, 129.4, 130.0, 130.4, 131.0, 131.9, 133.1, 133.2, 136.2, 137.2, 141.2, 169.1 ppm. HRMS (ESI): calcd for $C_{21}H_{20}Cl_2NO$ $[M+H]^+$ 372.0916, 374.0887, 373.0950, found 372.0920, 374.0891, 373.0952.

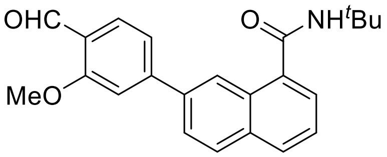

***N*-(*tert*-Butyl)-7-(4-formyl-3-methoxyphenyl)-1-naphthamide (3o)**

Following the general procedure, the reaction of *N*-(*tert*-butyl)-1-naphthamide (**1a**) (45.4 mg, 0.20 mmol), (4-formyl-3-methoxyphenyl)(mesityl)iodonium triflate (**2o**) (159.6 mg, 0.30 mmol), $Cu(OTf)_2$ (7.2 mg, 10 mol %), in DCE (1 mL) at 60 °C. After 24 h, purification by column chromatography on silica gel (PE/DCM/EA = 10/10/1, v/v/v) yields **3o** (42.1 mg, 58%) as colorless oil. ^{1}H NMR (400 MHz, $CDCl_3$): δ = 1.55 (s, 9H), 4.00 (s, 3H), 5.88 (bs, 1H), 7.12 (d, J = 8.8 Hz, 1H), 7.43 (dd, J = 8.4, 7.2 Hz, 1H), 7.58 (dd, J = 7.0, 1.2 Hz, 1H), 7.76 (dd, J = 8.5, 1.8 Hz, 1H), 7.87-7.96 (m, 3H), 8.17 (d, J = 2.5 Hz, 1H), 8.45 (t, J = 0.8 Hz 1H), 10.52 (s, 1H) ppm. ^{13}C NMR (100 MHz, $CDCl_3$): δ = 29.1, 52.3, 56.0, 112.5, 122.8, 124.99, 125.04, 125.1, 125.7, 127.3, 129.1, 130.0, 130.4, 132.9, 133.7, 134.9, 136.1, 138.0, 161.6, 169.2, 189.9 ppm. HRMS (ESI): calcd for $C_{23}H_{24}NO_3$ $[M+H]^+$ 362.1751, found 362.1757.

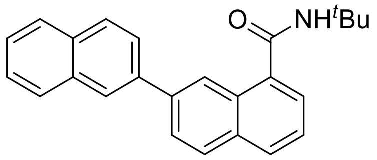

***N-(tert*-Butyl)-[2,2'-binaphthalene]-8-carboxamide (3p)**

Following the general procedure, the reaction of *N*-(*tert*-butyl)-1-naphthamide (**1a**) (45.4 mg, 0.20 mmol), mesityl(naphthalen-2-yl)iodonium triflate (**2p**) (156.6 mg, 0.30 mmol), $Cu(OTf)_2$ (7.2 mg, 10 mol %), in DCE (1 mL) at 70 °C. After 24 h, purification by column chromatography on silica gel (PE/THF = 20/1, v/v) yields **3p** (46.6 mg, 66%) as colorless oil. ^{1}H NMR (400 MHz, $CDCl_3$): δ = 1.56 (s, 9H), 5.89 (s, 1H), 7.43-7.48 (m, 1H), 7.48-7.56 (m, 2H), 7.60 (dd, J = 6.8, 1.2 Hz, 1H), 7.86-7.98 (m, 7H), 8.17-8.19 (m, 1H), 8.63 (t, J = 0.8, Hz 1H) ppm. ^{13}C NMR (100 MHz, $CDCl_3$): δ = 29.1, 52.3, 76.8, 77.2, 77.45, 123.7, 125.0, 125.1, 125.83, 126.2, 126.35, 126.41, 126.5, 127.8, 128.5, 128.7, 129.0, 130.0, 130.5, 132.8, 133.0, 133.8, 136.2, 138.4, 139.6, 169.3 ppm. The analytical data matched those reported in the literature.[1]

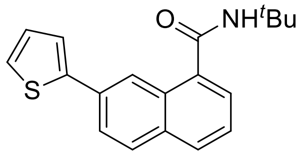

***N*-(*tert*-Butyl)-7-(thiophen-2-yl)-1-naphthamide (3q)**

Following the general procedure, the reaction of *N*-(*tert*-butyl)-1-naphthamide (**1a**) (45.4 mg, 0.20 mmol), mesityl(thiophen-2-yl)iodonium triflate (**2q**) (111.3 mg, 0.30 mmol), $Cu(OTf)_2$ (7.2 mg, 10 mol %), in DCE (1 mL) at 50 °C. After 24 h, purification by column chromatography on silica gel (PE/THF = 20/1, v/v) yields **3q** (21.6 mg, 35%) as colorless oil ^{1}H NMR (400 MHz, $CDCl_3$): δ = 1.57 (s, 9H), 5.84 (bs, 1H), 7.10-7.14 (m, 1H), 7.33 (dd, J = 5.2, 1.2 Hz, 1H), 7.39-7.44 (m, 1H), 7.45 (dd, J = 3.6, 0.8 Hz, 1H), 7.57 (dd, J = 7.2, 1.2 Hz, 1H), 7.78 (dd, J = 8.4, 1.6 Hz, 1H), 7.85 (dd, J = 8.4, 3.6 Hz, 2H), 8.48 (s, 1H) ppm. ^{13}C NMR (100 MHz, $CDCl_3$): δ = 29.1, 52.3, 121.7, 123.9, 124.9, 125.0, 125.4, 125.6, 128.4, 129.1, 129.9, 130.4, 132.9, 133.0, 136.1, 144.5, 169.1 ppm. HRMS (ESI): calcd for $C_{19}H_{20}NOS$ $[M+H]^+$ 310.1260, found 310.1262.

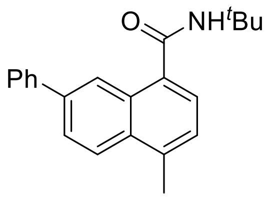

***N-(tert*-Butyl)-4-methyl-7-phenyl-1-naphthamide (4a)**

Following the general procedure, the reaction of *N-(tert*-butyl)-4-methyl-1-naphthamide (**1b**) (48.2 mg, 0.20 mmol), mesityl(phenyl)iodonium triflate (**2a**) (141.3 mg, 0.30 mmol), Cu(OTf)$_2$ (7.2 mg, 10 mol %), in DCE (1 mL) at 70 °C. After 24 h, purification by column chromatography on silica gel (PE/THF = 20/1, v/v) yields **4a** (56.5 mg, 89%) as a white solid. ^{1}H NMR (400 MHz, CDCl$_3$): δ = 1.54 (s, 9H), 2.73 (d, J = 0.8 Hz, 3H), 5.83 (s, 1H), 7.27 (dd, J = 7.2, 0.8 Hz, 1H), 7.36-7.41 (m, 1H), 7.46-7.51 (m, 3H), 7.72-7.75 (m, 2H), 7.82 (dd, J = 8.8, 2.0 Hz, 1H), 8.09 (d, J = 9.2 Hz, 1H), 8.54 (d, J = 1.6 Hz, 1H) ppm. ^{13}C NMR (100 MHz, CDCl$_3$): δ = 19.8, 29.1, 52.1, 124.0, 124.8, 125.1, 125.6, 125.9, 127.59, 127.61, 129.0, 130.6, 132.0, 134.7, 136.7, 139.2, 141.1, 169.5 ppm. The analytical data matched those reported in the literature.[1]

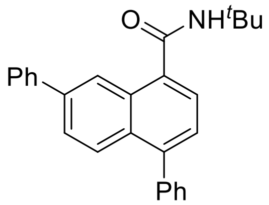

***N-(tert*-Butyl)-4,7-diphenyl-1-naphthamide (4b)**

Following the general procedure, the reaction of *N-(tert*-butyl)-4-phenyl-1-naphthamide (**1c**) (60.6 mg, 0.20 mmol), mesityl(phenyl)iodonium triflate (**2a**) (141.3 mg, 0.30 mmol), Cu(OTf)$_2$ (7.2 mg, 10 mol %), in DCE (1 mL) at 70 °C. After 24 h, purification by column chromatography on silica gel (PE/THF = 10/1, v/v) yields **4b** (66.7 mg, 88%) as a white solid. ^{1}H NMR (400 MHz, CDCl$_3$): δ = 1.57 (s, 9H), 5.91 (s, 1H), 7.37-7.42 (m, 2H), 7.46-7.55 (m, 7H), 7.62 (d, J = 7.2 Hz, 1H), 7.71-7.75 (m, 3H), 7.98 (d, J = 8.8 Hz, 1H), 8.56 (d, J = 1.6 Hz, 1H) ppm. ^{13}C NMR (100 MHz, CDCl$_3$): δ = 29.1, 52.3, 123.6, 124.5, 125.9, 126.2, 127.2, 127.6, 127.7, 127.78, 128.5, 129.1, 130.1, 130.9, 131.2, 135.8, 139.4, 140.3, 140.9, 142.3, 169.4 ppm. The analytical data matched those reported in the literature.[1]

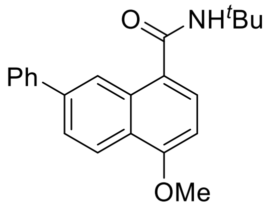

***N-(tert*-Butyl)-4-methoxy-7-phenyl-1-naphthamide (4c)**

Following the general procedure, the reaction of *N-(tert*-butyl)-4-methoxy-1-naphthamide (**1d**) (51.4 mg, 0.20 mmol), mesityl(phenyl)iodonium triflate (**2a**) (141.3 mg, 0.30 mmol), $Cu(OTf)_2$ (7.2 mg, 10 mol %), in DCE (1 mL) at 70 °C. After 24 h, purification by column chromatography on silica gel (PE/THF = 10/1, v/v) yields **4c** (54.0 mg, 81%) as a white solid. ^{1}H NMR (400 MHz, $CDCl_3$): δ = 1.53 (s, 9H), 4.03 (s, 3H), 5.82 (s, 1H), 6.74 (d, J = 8.0 Hz, 1H), 7.36-7.41 (m, 1H), 7.48 (t, J = 7.6 Hz, 2H), 7.54 (d, J = 8.0 Hz, 1H), 7.71-7.75 (m, 2H), 7.77 (dd, J = 8.8, 1.6 Hz, 1H), 8.35 (d, J = 8.8 Hz, 1H), 8.54 (d, J = 1.6 Hz, 1H) ppm. ^{13}C NMR (100 MHz, $CDCl_3$): δ = 29.1, 52.0, 55.8, 102.5, 123.0, 123.3, 124.9, 125.4, 126.1, 127.63, 127.64, 128.7, 129.0, 131.8, 140.1, 141.2, 157.0, 169.3 ppm. The analytical data matched those reported in the literature.[1]

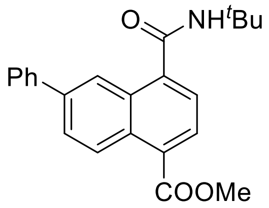

Methyl 4-(*tert*-butylcarbamoyl)-6-phenyl-1-naphthoate (4d)

Following the general procedure, the reaction of methyl 4-(*tert*-butylcarbamoyl)-1-naphthoate (**1e**) (57.0 mg, 0.20 mmol), mesityl(phenyl)iodonium triflate (**2a**) (141.3 mg, 0.30 mmol), $Cu(OTf)_2$ (7.2 mg, 10 mol %), in DCE (1 mL) at 70 °C. After 24 h, purification by column chromatography on silica gel (PE/DCM/EA = 20/20/1, v/v/v) yields **4d** (51.9 mg, 72%) as colorless oil ^{1}H NMR (400 MHz, $CDCl_3$): δ = 1.55 (s, 9H), 4.02 (s, 3H), 5.91 (bs, 1H), 7.38-7.42 (m, 1H), 7.47-7.54 (m, 3H), 7.72 (dd, J = 8.4, 1.2 Hz, 2H), 7.89 (dd, J = 9.2, 2.0 Hz, 1H), 8.08 (d, J = 7.2 Hz, 1H), 8.43 (d, J = 2.0 Hz, 1H), 8.96 (d, J = 9.2 Hz, 1H) ppm. ^{13}C NMR (100 MHz, $CDCl_3$): δ = 29.0, 52.54, 52.55, 123.3, 123.4, 126.7, 127.5, 127.7, 127.9, 128.6, 129.08, 129.12, 130.8, 130.9, 139.6,

140.5, 140.7, 167.6, 168.7 ppm. HRMS (ESI): calcd for $C_{23}H_{24}NO_3$ $[M+H]^+$ 362.1751 , found 362.1752.

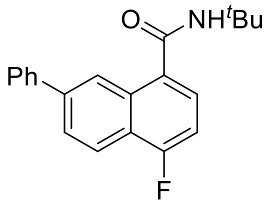

N-(*tert*-Butyl)-4-fluoro-7-phenyl-1-naphthamid*e* (4e)

Following the general procedure, the reaction of N-(*tert*-butyl)-4-fluoro-1-naphthamide (**1f**) (49.0 mg, 0.20 mmol), mesityl(phenyl)iodonium triflate (**2a**) (141.3 mg, 0.30 mmol), $Cu(OTf)_2$ (7.2 mg, 10 mol %), in DCE (1 mL) at 70 °C. After 24 h, purification by column chromatography on silica gel (PE/THF = 10/1, v/v) yields **4e** (45.6 mg, 71%) as a white solid. ^{1}H NMR (400 MHz, $CDCl_3$): δ = 1.54 (s, 9H), 5.86 (bs, 1H), 7.05-7.10 (m, 1H), 7.38-7.43 (m, 1H), 7.47-7.54 (m, 3H), 7.70-7.73 (m, 2H), 7.84 (dd, J = 8.8, 1.6 Hz, 1H), 8.19 (d, J = 8.8 Hz, 1H), 8.52 (s, 1H) ppm. ^{13}C NMR (100 MHz, $CDCl_3$): δ = 29.1, 52.3, 108.4 (d, J_{C-F} = 21 Hz), 121.5 (d, J_{C-F} = 5 Hz), 123.0, 123.1, 123.5 (d, J_{C-F} = 2 Hz), 125.4 (d, J_{C-F} = 9 Hz), 126.6 (d, J_{C-F} = 2 Hz), 127.7, 128.0, 129.1, 132.3 (d, J_{C-F} = 4 Hz), 132.4 (d, J_{C-F} = 5 Hz), 140.7, 159.7 (d, J_{C-F} = 254 Hz), 168.6 ppm. The analytical data matched those reported in the literature.[1]

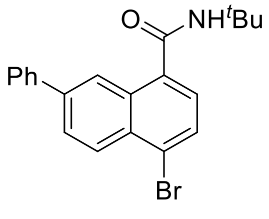

4-Bromo-N-(*tert*-butyl)-7-phenyl-1-naphthamid*e* (4f)

Following the general procedure, the reaction of N-(*tert*-butyl)-4-phenyl-1-naphthamide (**1g**) (61.2 mg, 0.20 mmol), mesityl(phenyl)iodonium triflate (**2a**) (141.3 mg, 0.30 mmol), $Cu(OTf)_2$ (7.2 mg, 10 mol %), in DCE (1 mL) at 70 °C. After 24 h, purification by column chromatography on silica gel (PE/DCM/EA = 50/110/1, v/v/v) yields **4f** (57.2 mg, 75%) as a white solid. ^{1}H NMR (400 MHz, $CDCl_3$): δ = 1.53 (s, 9H), 5.87 (bs, 1H), 7.37-7.43 (m, 2H), 7.47-7.52 (m, 2H), 7.70-7.74 (m, 3H), 7.88 (dd, J = 8.8, 1.6 Hz, 1H), 8.33 (d, J = 9.6 Hz, 1H), 8.47 (d, J = 1.6 Hz, 1H) ppm. ^{13}C NMR

(100 MHz, $CDCl_3$): δ = 29.0, 52.4, 123.7, 124.8, 125.1, 127.5, 127.6, 128.0, 128.2, 128.9, 129.1, 131.4, 131.7, 136.1, 140.4, 168.6 ppm. The analytical data matched those reported in the literature.[1]

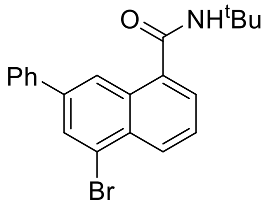

5-Bromo-*N*-(*tert*-butyl)-7-phenyl-1-naphthamide (4g)

Following the general procedure, the reaction of 5-bromo-*N*-(*tert*-butyl)-1-naphthamide (**1h**) (61.0 mg, 0.20 mmol), mesityl(phenyl)iodonium triflate (**2a**) (141.3 mg, 0.30 mmol), $Cu(OTf)_2$ (7.2 mg, 10 mol %), in DCE (1 mL) at 70 °C. After 24 h, purification by column chromatography on silica gel (PE/THF = 20/1, v/v) yields **4g** (51.1 mg, 67%) as colorless oil ^1H NMR (400 MHz, $CDCl_3$): δ = 1.54 (s, 9H), 5.84 (bs, 1H), 7.40 (t, J = 7.2 Hz, 1H), 7.46-7.57 (m, 3H), 7.59-7.71 (m, 3H), 8.11 (s, 1H), 8.32 (d, J = 8.4 Hz, 1H), 8.45 (s, 1H) ppm. ^{13}C NMR (100 MHz, $CDCl_3$): δ = 29.1, 52.4, 123.3, 123.7, 125.7, 126.3, 127.5, 128.1, 129.1, 129.2, 130.2, 131.3, 131.6, 136.7, 139.7, 140.2, 168.8 ppm. HRMS (ESI): calcd for $C_{21}H_{21}BrNO$ $[M+H]^+$ 382.0801, 384.0781, found 382.0809, 384.0782.

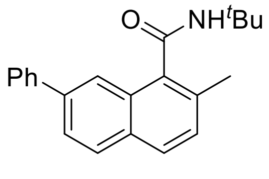

***N*-(*tert*-Butyl)-2-methyl-7-phenyl-1-naphthamide (4h)**

Following the general procedure, the reaction of *N-(tert*-butyl)-2-methyl-1-naphthamide (**1i**) (48.2 mg, 0.20 mmol), mesityl(phenyl)iodonium triflate (**2a**) (141.3 mg, 0.30 mmol), $Cu(OTf)_2$ (7.2 mg, 10 mol %), in DCE (1 mL) at 70 °C. After 24 h, purification by column chromatography on silica gel (PE/THF = 20/1, v/v) yields **4h** (53.9 mg, 85%) as a white solid. ^1H NMR (400 MHz, $CDCl_3$): δ = 1.56 (s, 9H), 2.53 (s, 3H), 5.71 (bs, 1H), 7.30 (d, J = 8.4 Hz, 1H), 7.36-7.40 (m, 1H), 7.46-7.51 (m, 2H), 7.68-7.73 (m, 3H), 7.77 (d, J = 8.4 Hz, 1H), 7.87 (d, J = 8.4 Hz, 1H), 8.10 (s, 1H) ppm. ^{13}C NMR (100 MHz, $CDCl_3$): δ = 19.5, 29.1, 52.3, 122.6, 125.2, 127.5, 127.6, 128.4,

128.57, 128.62, 129.1, 130.5, 131.0, 132.2, 135.2, 139.4, 141.2, 169.3 ppm. The analytical data matched those reported in the literature.[1]

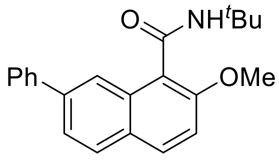

N-(tert-Butyl)-2-methoxy-7-phenyl-1-naphthamide (4i)

Following the general procedure, the reaction of *N-(tert*-butyl)-2-methoxy-1-naphthamide (**1j**) (51.4 mg, 0.20 mmol), mesityl(phenyl)iodonium triflate (**2a**) (141.3 mg, 0.30 mmol), $Cu(OTf)_2$ (7.2 mg, 10 mol %), in DCE (1 mL) at 70 °C. After 24 h, purification by column chromatography on silica gel (PE/THF = 15/1, v/v) yields **4i** (53.3 mg, 80%) as a white solid. ^{1}H NMR (400 MHz, $CDCl_3$): δ = 1.55 (s, 9H), 3.97 (s, 3H), 5.80 (bs, 1H), 7.25 (d, J = 8.8 Hz, 1H), 7.35-7.40 (m, 1H), 7.45-7.50 (m, 2H), 7.63 (dd, J = 8.6, 1.6 Hz, 1H), 7.69-7.72 (m, 2H), 7.84 (d, J = 5.6 Hz, 1H), 7.86 (d, J = 6.4 Hz, 1H), 8.14 (s, 1H) ppm. ^{13}C NMR (100 MHz, $CDCl_3$): δ = 29.1, 52.2, 57.0, 113.5, 122.2, 122.4, 124.0, 127.5, 127.6, 128.1, 128.5, 129.0, 130.5, 131.8, 140.0, 141.2, 153.7, 167.0 ppm. The analytical data matched those reported in the literature.[1]

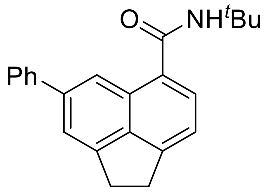

2,2-Dimethyl-1-(7-phenylnaphthalen-1-yl)propan-1-one (4j)

Following the general procedure, the reaction of *N-(tert*-butyl)-1,2-dihydroacenaphthylene-5-carboxamide (**1k**) (50.6 mg, 0.20 mmol), mesityl(phenyl)iodonium triflate (**2a**) (141.3 mg, 0.30 mmol), $Cu(OTf)_2$ (7.2 mg, 10 mol %), in DCE (1 mL) at 70 °C. After 24 h, purification by column chromatography on silica gel (PE/THF = 20/1, v/v) yields **4j** (34.9 mg, 53%) as a white solid. ^{1}H NMR (400 MHz, $CDCl_3$): δ = 1.54 (s, 9H), 3.40-3.49 (m, 4H), 5.89 (bs, 1H), 7.24 (dt, J = 7.2, 1.2 Hz, 1H), 7.35-7.40 (m, 1H), 7.45-7.50 (m, 2H), 7.57 (d, J = 1.2 Hz, 1H), 7.64 (d, J = 7.2 Hz, 1H), 7.69-7.72 (m, 2H), 8.31 (d, J = 1.2 Hz, 1H) ppm. ^{13}C NMR (100 MHz, $CDCl_3$): δ = 29.2, 30.5, 30.6, 52.0, 118.5, 119.8, 120.1, 127.4, 127.8, 128.0, 128.9, 131.0, 139.0, 142.3, 142.5, 146.9, 149.0, 168.7 ppm. HRMS (ESI): calcd for $C_{23}H_{24}NO_3$

[M+H]$^+$ 330.1852, found 330.1759.

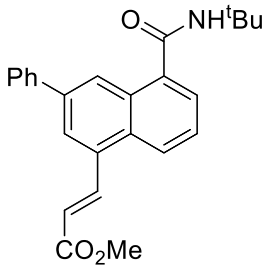

***(E)*-Methyl 3-(5-(*tert*-butylcarbamoyl)-3-phenylnaphthalen-1-yl)acrylate (4k)**

Following the general procedure, the reaction of (*E*)-methyl 3-(5-(*tert*-butylcarbamoyl)naphthalen-1-yl)acrylate (**1l**) (62.2 mg, 0.20 mmol), mesityl(phenyl)iodonium triflate (**2a**) (141.3 mg, 0.30 mmol), Cu(OTf)$_2$ (7.2 mg, 10 mol %), in DCE (1 mL) at 70 °C. After 24 h, purification by column chromatography on silica gel (PE/DCM/EA = 20/20/1, v/v/v) yields **4k** (54.2 mg, 70%) as colorless oil. ^{1}H NMR (400 MHz, CDCl$_3$): δ = 1.55 (s, 9H), 3.87 (s, 3H), 5.87 (bs, 1H), 6.59 (d, J = 16.0 Hz, 1H), 7.39-7.43 (m, 1H), 7.47-7.55 (m, 3H), 7.59-7.62 (m, 1H), 7.69-7.73 (m, 2H), 8.02 (d, J = 1.6 Hz, 1H), 8.23 (d, J = 8.4 Hz, 1H), 8.53 (d, J = 15.6), 8.54 (s, 1H) ppm. ^{13}C NMR (100 MHz, CDCl$_3$): δ = 29.1, 52.0, 52.4, 121.5, 125.2, 125.3, 125.4, 125.7, 125.82, 127.6, 128.0, 129.2, 130.87, 130.89, 132.8, 137.0, 139.2, 140.5, 142.0, 167.3, 169.1 ppm. HRMS (ESI): calcd for $C_{25}H_{26}NO_3$ [M+H]$^+$, 388.1907, found 388.1906.

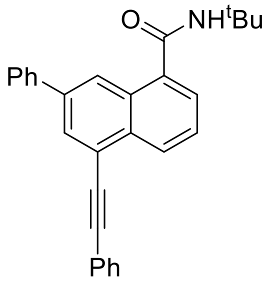

***N*-(*tert*-Butyl)-7-phenyl-5-(phenylethynyl)-1-naphthamide (4l)**

Following the general procedure, the reaction of *N*-(*tert*-butyl)-5-(phenylethynyl)-1-naphthamide (**1m**) (65.4 mg, 0.20 mmol), mesityl(phenyl)iodonium triflate (**2a**) (141.3 mg, 0.30 mmol), Cu(OTf)$_2$ (7.2 mg, 10 mol %), in DCE (1 mL) at 70 °C. After 24 h, purification by column chromatography on silica gel (PE/THF = 20/1, v/v) yields **4l** (48.4 mg, 60%) as colorless oil. ^{1}H NMR (400 MHz, CDCl$_3$): δ = 1.55 (s, 9H), 5.88 (bs,

1H), 7.37-7.44 (m, 4H), 7.47-7.52 (m, 2H), 7.52-7.57 (m, 1H), 7.62 (dd, J = 6.8, 1.2 Hz, 1H), 7.65-7.69 (m, 2H), 7.72-7.76 (m, 2H), 8.08 (d, J = 1.6 Hz, 1H), 8.49-8.53 (m, 2H) ppm. ^{13}C NMR (100 MHz, $CDCl_3$): δ = 29.1, 52.3, 87.5, 94.8, 122.0, 123.3, 124.1, 125.5, 125.7, 127.6, 127.9, 128.3, 128.6, 128.7, 129.1, 130.50, 130.54, 131.8, 132.7, 136.6, 139.2, 140.4, 169.1ppm. HRMS (ESI): calcd for $C_{29}H_{26}NO$ [M+H]$^+$ 404.2009, found 404.2016.

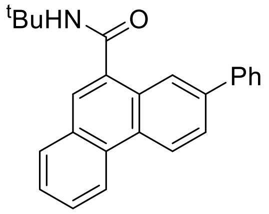

***N*-(*tert*-Butyl)-7-phenylphenanthrene-9-carboxamide (4m)**

Following the general procedure, the reaction of *N*-(*tert*-butyl)phenanthrene-9-carboxamide (**1n**) (55.4 mg, 0.20 mmol), mesityl(phenyl)iodonium triflate (**2a**) (141.3 mg, 0.30 mmol), Cu(OTf)$_2$ (7.2 mg, 10 mol %), in DCE (1 mL) at 70 °C. After 24 h, purification by column chromatography on silica gel (PE/THF = 20/1, v/v) yields **4m** (52.4 mg, 74%) as a white solid. ^{1}H NMR (400 MHz, $CDCl_3$): δ = 1.58 (s, 9H), 5.97 (bs, 1H), 7.38-7.42 (m, 1H), 7.48-7.53 (m, 2H), 7.60-7.65 (m, 1H), 7.68-7.73 (m, 1H), 7.74-7.78 (m, 2H), 7.83 (s, 1H), 7.90 (dd, J = 8.0, 1.6 Hz, 1H), 7.94 (dd, J = 8.4, 2.0 Hz, 1H), 8.53 (d, J = 1.8 Hz, 1H), 8.67 (d, J = 7.8 Hz, 1H), 8.75 (d, J = 8.6 Hz, 1H) ppm. ^{13}C NMR (100 MHz, $CDCl_3$): δ = 29.1, 52.3, 122.8, 123.6, 124.4, 126.1, 126.4, 127.2, 127.5, 127.7, 127.9, 129.08, 129.14, 129.2, 129.8, 130.6, 130.8, 135.1, 139.8, 140.9, 169.3 ppm. HRMS (ESI): calcd for $C_{25}H_{23}NNaO_3$ [M+Na]$^+$ 376.1672, found 376.1679.

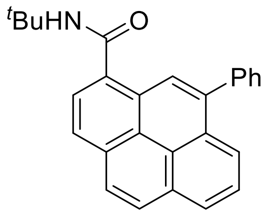

***N*-(*tert*-Butyl)-9-phenylpyrene-1-carboxamide (4n)**

Following the general procedure, the reaction of *N-(tert*-butyl)pyrene-1-carboxamide (**1o**) (60.2 mg, 0.20 mmol), mesityl(phenyl)iodonium triflate (**2a**) (141.3 mg, 0.30

mmol), Cu(OTf)$_2$ (7.2 mg, 10 mol %), in DCE (1 mL) at 70 °C. After 24 h, purification by column chromatography on silica gel (PE/THF = 20/1, v/v) yields **6c** (49.0 mg, 65%) as a white solid. ^{1}H NMR (400 MHz, CDCl$_3$): δ = 1.57 (s, 9H), 5.96 (bs, 1H), 7.48-7.53 (m, 1H), 7.54-7.59 (m, 2H), 7.65-7.69 (m, 2H), 7.98 (t, J = 8.0 Hz, 1H), 8.06-8.09 (m, 2H), 8.13-8.16 (m, 2H), 8.22-8.26 (m, 2H), 8.50 (s, 1H) ppm. ^{13}C NMR (100 MHz, CDCl$_3$): δ = 29.2, 52.4, 124.4, 124.5, 124.7, 124.8, 124.97, 124.99, 126.1, 126.3, 127.2, 127.8, 128.2, 128.6, 128.8, 130.2, 130.3, 131.6, 132.3, 132.8, 140.8, 140.9, 169.6 ppm. HRMS (ESI): calcd for $C_{27}H_{24}NO$ [M+H]$^+$, 378.1852, found 378.1858.

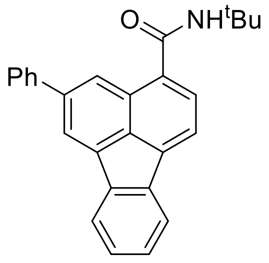

***N*-(*tert*-Butyl)-5-phenylfluoranthene-3-carboxamide (4o)**

Following the general procedure, the reaction of *N*-(*tert*-butyl)fluoranthene-3-carboxamide (**1p**) (60.2 mg, 0.20 mmol), mesityl(phenyl)iodonium triflate (**2a**) (141.3 mg, 0.30 mmol), Cu(OTf)$_2$ (7.2 mg, 10 mol %), in DCE (1 mL) at 70 °C. After 24 h, purification by column chromatography on silica gel (PE/THF = 20/1, v/v) yields **4o** (36.2 mg, 48%) as a yellow solid. ^{1}H NMR (400 MHz, CDCl$_3$): δ = 1.57 (s, 9H), 6.02 (bs, 1H), 7.38-7.45 (m, 3H), 7.50-7.55 (m, 2H), 7.75-7.79 (m, 3H), 7.82 (d, J = 7.2 Hz, 1H), 7.86-7.89 (m, 1H), 7.90-7.93 (m, 1H), 8.14 (d, J = 1.2 Hz, 1H), 8.48 (d, J = 1.2 Hz, 1H) ppm. ^{13}C NMR (100 MHz, CDCl$_3$): δ = 29.2, 52.3, 119.0, 120.8, 121.8, 122.1, 124.0, 127.61, 127.62, 127.7, 127.97, 128.01, 128.4, 129.0, 132.2, 134.8, 137.7, 139.10, 139.12, 140.0, 142.0, 142.7, 168.2 ppm. HRMS (ESI): calcd for $C_{27}H_{24}NO$ [M+H]$^+$ 378.1852, found 378.1861.

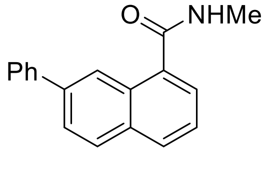

***N*-Methyl-7-phenyl-1-naphthamide (4p)**

Following the general procedure, the reaction of *N*-methyl-1-naphthamide (**1q**) (37.0

mg, 0.20 mmol), mesityl(phenyl)iodonium triflate (**2a**) (141.3 mg, 0.30 mmol), $Cu(OTf)_2$ (7.2 mg, 10 mol %), in DCE (1 mL) at 70 °C. After 24 h, purification by column chromatography on silica gel (PE/THF = 20/1, v/v) yields **4p** (35.0 mg, 67%) as a white solid. ^{1}H NMR (400 MHz, $CDCl_3$): δ = 3.09 (d, J = 4.8 Hz, 3H), 6.07 (bs, 1H), 7.36-7.40 (m, 1H), 7.41-7.45 (m, 1H), 7.45-7.50 (m, 2H), 7.59 (dd, J = 7.2, 1.2 Hz, 1H), 7.71-7.74 (m, 2H), 7.79 (dd, J = 8.4, 2.0 Hz, 1H), 7.90-7.94 (m, 2H), 8.51 (s, 1H) ppm. ^{13}C NMR (100 MHz, $CDCl_3$): δ = 27.1, 123.6, 124.8, 125.4, 126.3, 127.7, 127.7, 128.9, 129.0, 130.45, 130.53, 133.0, 134.8, 139.9, 141.0, 170.4 ppm. The analytical data matched those reported in the literature.[1]

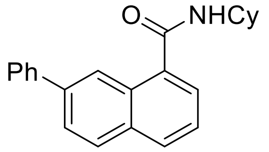

N-Cyclohexyl-7-phenyl-1-naphthamide (4q)

Following the general procedure, the reaction of *N*-cyclohexyl-1-naphthamide (**1r**) (50.6 mg, 0.20 mmol), mesityl(phenyl)iodonium triflate (**2a**) (141.3 mg, 0.30 mmol), $Cu(OTf)_2$ (7.2 mg, 10 mol %), in DCE (1 mL) at 70 °C. After 24 h, purification by column chromatography on silica gel (PE/THF = 20/1, v/v) yields **4q** (55.3 mg, 84%) as a white solid. ^{1}H NMR (400 MHz, $CDCl_3$): δ = 1.14-1.32 (m, 3H), 1.41-1.53 (m, 2H), 1.64-1.71 (m, 1H), 1.73-1.81 (m, 2H), 2.08-2.16 (m, 2H), 4.07-4.16 (m, 1H), 5.92 (d, J = 8.4 Hz, 1H), 7.36-7.41 (m, 1H), 7.42-7.51 (m, 3H), 7.60 (dd, J = 6.8, 1.2 Hz, 1H), 7.71-7.75 (m, 2H), 7.79 (dd, J = 8.4, 1.6 Hz, 1H), 7.92 (t, J = 7.6 Hz, 2H), 8.53 (s, 1H) ppm. ^{13}C NMR (100 MHz, $CDCl_3$): δ = 25.0, 25.7, 33.4, 48.9, 123.5, 124.9, 125.3, 126.2, 127.66, 127.68, 128.9, 129.0, 130.3, 130.5, 133.0, 135.3, 139.8, 141.0, 168.8 ppm. The analytical data matched those reported in the literature.[1]

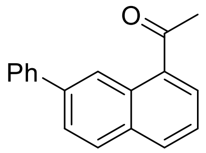

1-(7-Phenylnaphthalen-1-yl)ethanone (4r)

Following the general procedure, the reaction of 1-(naphthalen-1-yl)ethanone (**1s**) (34.0 mg, 0.20 mmol), mesityl(phenyl)iodonium triflate (**2a**) (141.3 mg, 0.30 mmol),

$Cu(OTf)_2$ (7.2 mg, 10 mol %), in DCE (1 mL) at 70 °C. After 24 h, purification by column chromatography on silica gel (PE/THF = 40/1, v/v) yields **4r** (21.1 mg, 43%) as a yellow oil. ^{1}H NMR (400 MHz, $CDCl_3$): δ = 2.78 (s, 3H), 7.37-7.42 (m, 1H), 7.47-7.53 (m, 3H), 7.74-7.77 (m, 2H), 7.82 (dd, J = 8.4, 1.6 Hz, 1H), 7.95 (d, J = 8.4 Hz, 1H), 7.98 (dd, J = 7.2, 1.2 Hz, 1H), 8.02 (d, J = 8.0 Hz, 1H), 9.03 (t, J = 0.8 Hz, 1H) ppm. ^{13}C NMR (100 MHz, $CDCl_3$): δ = 30.2, 124.2, 124.5, 126.2, 127.7, 127.8, 128.99, 129.04, 129.4, 130.6, 133.0, 133.3, 135.6, 140.8, 141.1, 202.0 ppm. HRMS (ESI): calcd for $C_{18}H_{15}O$ $[M+H]^+$ 247.1117, found 247.1124.

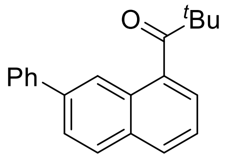

2,2-Dimethyl-1-(7-phenylnaphthalen-1-yl)propan-1-one (4s)

Following the general procedure, the reaction of 2,2-dimethyl-1-(naphthalen-1-yl)propan-1-one (**1t**) (42.4 mg, 0.20 mmol), mesityl(phenyl)iodonium triflate (**2a**) (141.3 mg, 0.30 mmol), $Cu(OTf)_2$ (7.2 mg, 10 mol %), in DCE (1 mL) at 70 °C. After 24 h, purification by column chromatography on silica gel (PE/THF = 60/1, v/v) yields **4s** (33.4 mg, 58%) as yellow oil. ^{1}H NMR (400 MHz, $CDCl_3$): δ = 1.35 (s, 9H), 7.36-7.41 (m, 2H), 7.50 (t, J = 1.2 Hz, 3H), 7.64-7.67 (m, 2H), 7.76 (dd, J = 8.4, 1.6 Hz, 1H), 7.79 (t, J = 0.8 Hz, 1H), 7.89 (d, J = 8.4 Hz, 1H), 7.94 (d, J = 8.4 Hz, 1H) ppm. ^{13}C NMR (100 MHz, $CDCl_3$): δ = 27.5, 45.7, 122.9, 123.5, 124.4, 126.2, 127.6, 127.7, 128.9, 129.1, 130.3, 132.9, 139.2, 139.6, 141.0, 214.8 ppm. HRMS (ESI): calcd for $C_{21}H_{20}NaO$ $[M+Na]^+$ 311.1406, found 311.1411.

VI. Photophysical data

Table S2. Photophysical data of 4k, 4n and 4o in toluene (1×10^{-5} mol/L) at 298 K.

Compound	$\lambda_{abs}{}^{a}$ (nm)	$\lambda_{em}{}^{b}$ (nm)
**4k**	322	395
**4n**	335/350	390
**4o**	351/370	477

^{a}UV-visible absorption peaks tested in toluene (1×10^{-5} mol/L). ^{b}Emission peaks tested in toluene (1×10^{-5} mol/L).

VII. References.

1. Zhang, M.; Luo, A.; Shi, Y.; Su, R.; Yang, Y.; You, J. *ACS Catal.* **2019,** 9, 11802-11807.

2. Bielawski, M.; Zhu, M.; Olofsson, B. *Adv. Synth. Catal*. **2007**, *349*, 2610-2618.

3. Bielawski, M.; AiliBerit, D.; Olofsson, B. *J. Org. Chem*. **2008**, *73*, 4602-4607.

4. Phipps, R. J.; Gaunt, M. J. *Science*. **2009**, *323*, 1593-1597.

5. Allen, A. E.; MacMillan, D. W. C. *J. Am. Chem. Soc*. **2011**, *133*, 4260-4263.

6. Wang, M.; Zhang, M.; Luo, Y.; Liu, Z.; Yang, C.; Lan, J.; Wu, D.; You, J. *Org. Lett*. **2020**, *22*, 135-139.

7. Kaicharla, T.; Thangaraj, M.; Biju, A. T. *Org. Lett*. **2014**, *16*, 1728-1731.

8. Wrona-Piotrowicz, A.; Zakrzewski, J.; Métivier, R.; Brosseau, A.; Makal, A.; Woźniakc, K. *RSC Adv*. **2014**, *4*, 56003-56012.

9. Yang, Z.-W.; Zhang, Q.; Jiang, Y.-Y.; Li, L.; Xiao, B.; Fu, Y. *Chem. Commun*. **2016**, *52*, 6709-6711.

VIII. Copies of ^{1}H and ^{13}C NMR spectra

^{1}H and ^{13}C NMR Spectra of substrates

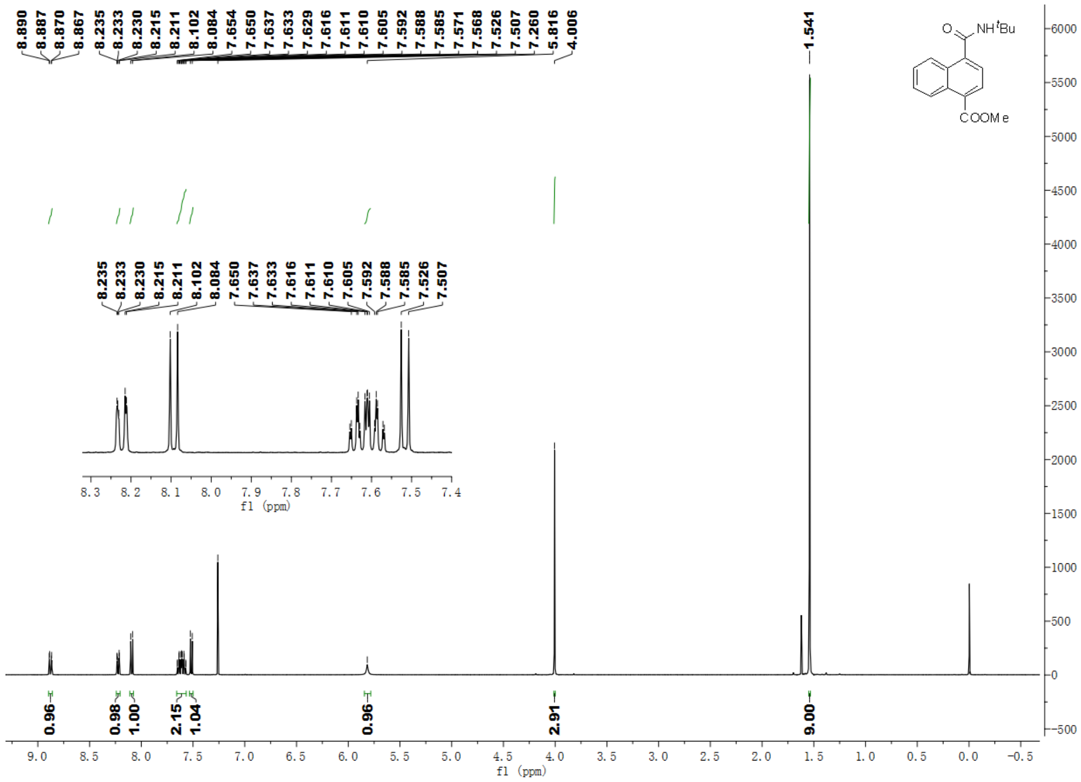

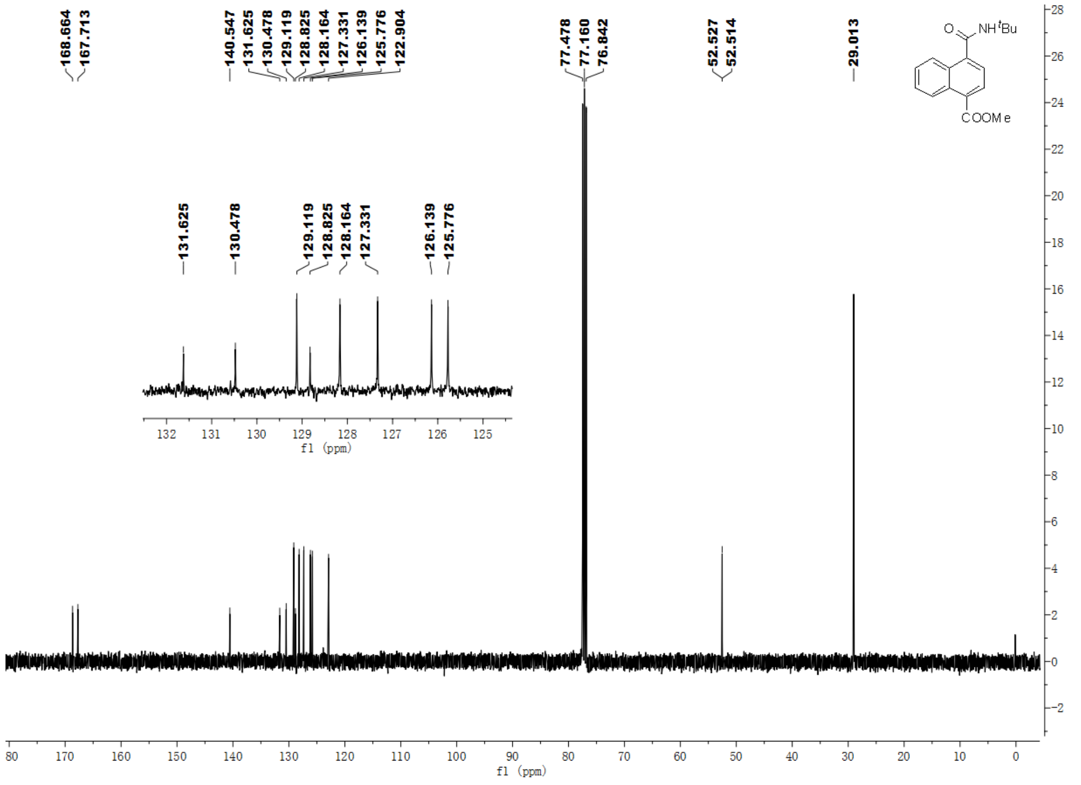

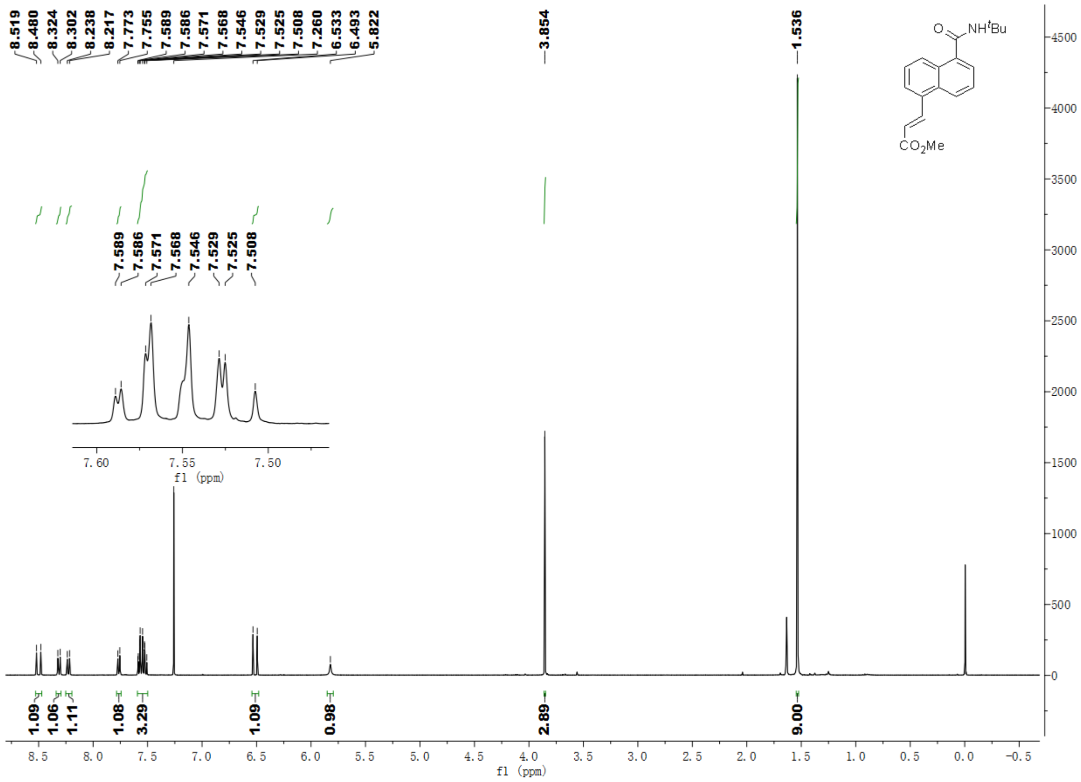
8.519
8.480
8.324
8.302
8.238
8.217
7.773
7.755
7.589
7.586
7.571
7.568
7.546
7.529
7.525
7.508
7.260
6.533
6.493
5.822
3.854
1.536
O
NH^{t}Bu
CO_2Me
1.09
1.06
1.11
1.08
3.29
1.09
0.98
2.89
9.00
f1 (ppm)

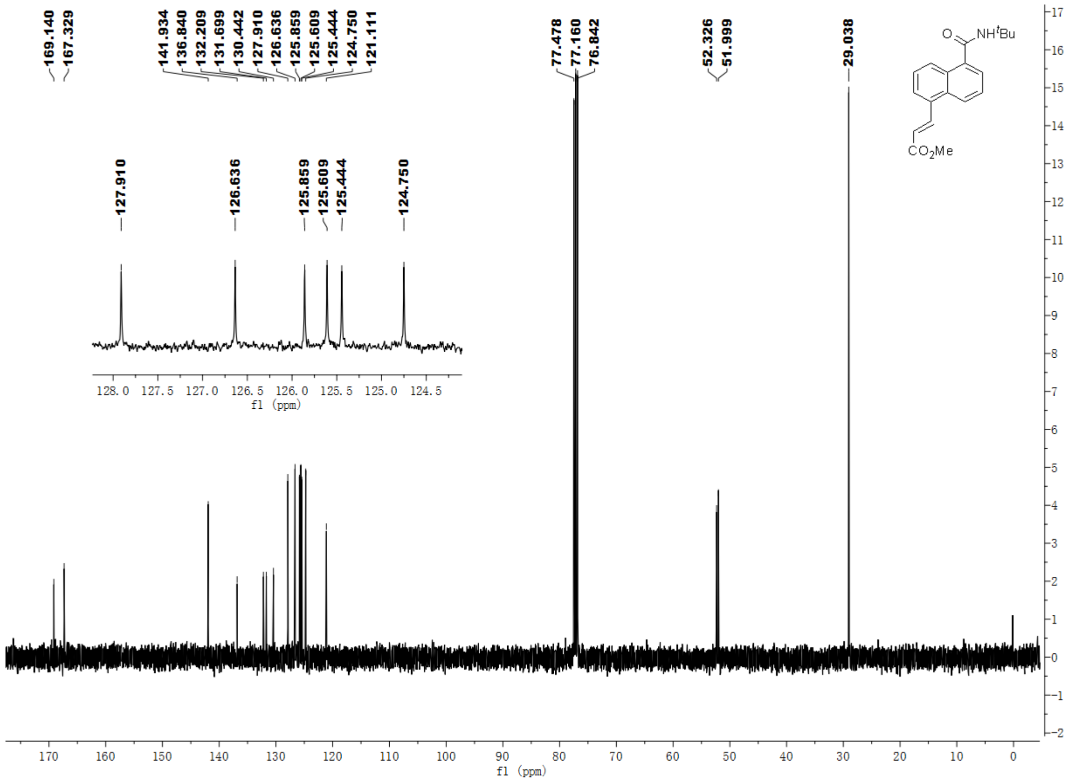
169.140
167.329
141.934
136.840
132.209
131.699
130.442
127.910
126.636
125.859
125.609
125.444
124.750
121.111
77.478
77.160
76.842
52.326
51.999
29.038
O
NH^{t}Bu
CO_2Me
f1 (ppm)

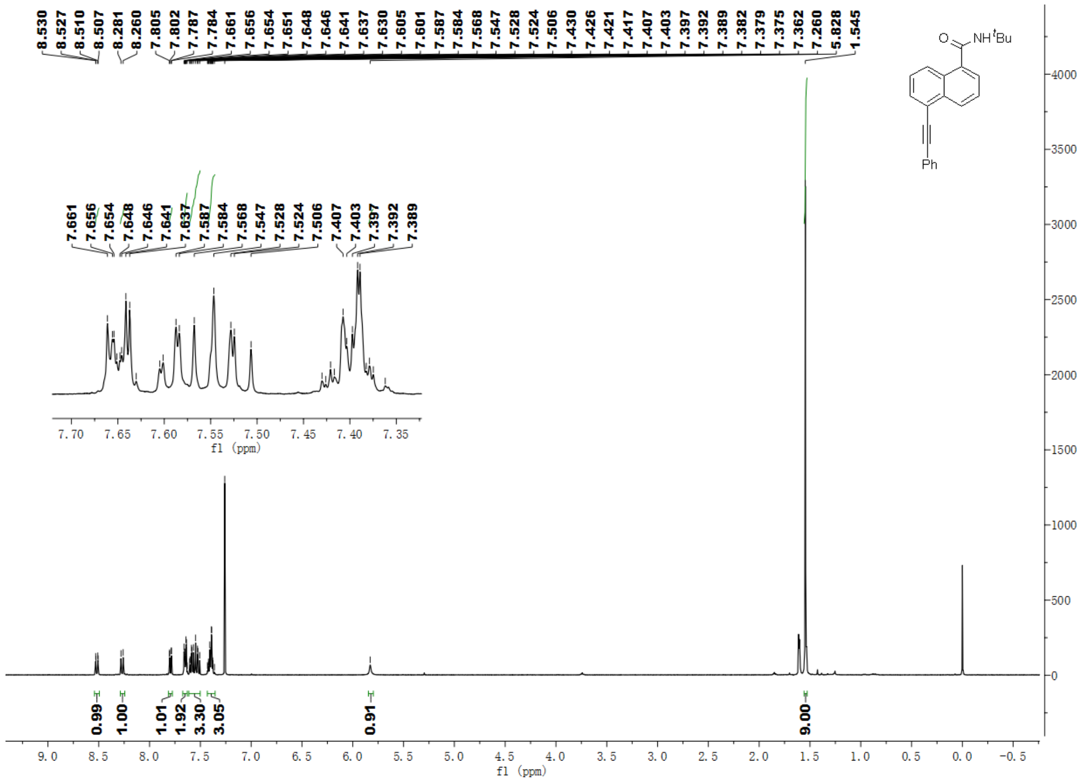

8.530
8.527
8.510
8.507
8.281
8.260
7.805
7.802
7.787
7.784
7.661
7.656
7.654
7.651
7.648
7.646
7.641
7.637
7.630
7.605
7.601
7.587
7.584
7.568
7.547
7.528
7.524
7.506
7.430
7.426
7.421
7.417
7.407
7.403
7.397
7.392
7.389
7.382
7.379
7.375
7.362
7.260
5.828
1.545
O
NH^{t}Bu
Ph
0.99
1.00
1.01
1.92
3.30
3.05
0.91
9.00
f1 (ppm)

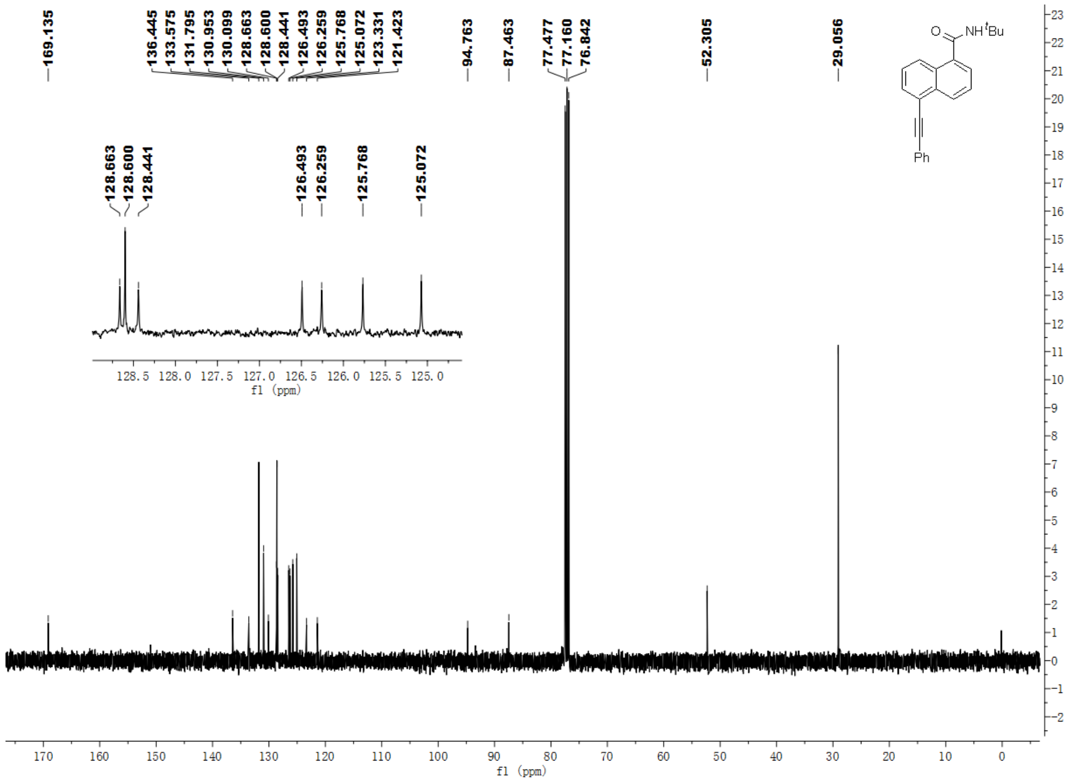

169.135
136.445
133.575
131.795
130.953
130.099
128.663
128.600
128.441
126.493
126.259
125.768
125.072
123.331
121.423
94.763
87.463
77.477
77.160
76.842
52.305
29.056
O
NH^{t}Bu
Ph
f1 (ppm)

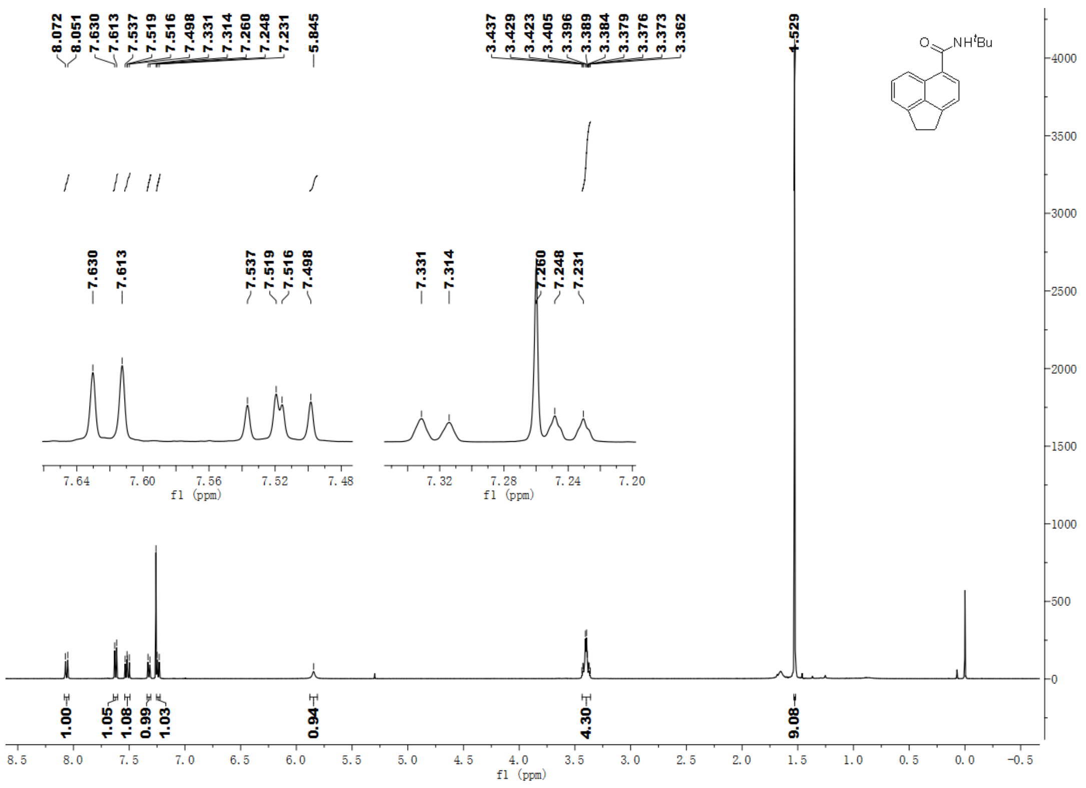
8.072
8.051
7.630
7.613
7.537
7.519
7.516
7.498
7.331
7.314
7.260
7.248
7.231
5.845
3.437
3.429
3.423
3.405
3.396
3.389
3.384
3.379
3.376
3.373
3.362
1.529
1.00
1.05
1.08
0.99
1.03
0.94
4.30
9.08
f1 (ppm)

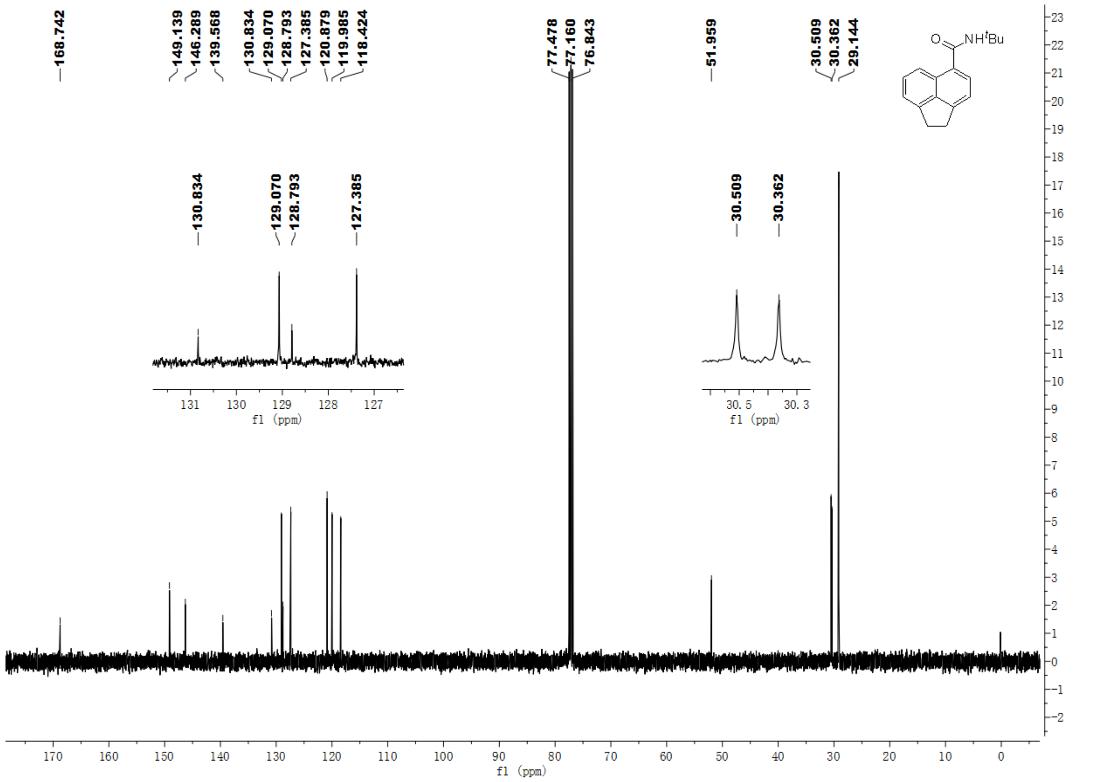
168.742
149.139
146.289
139.568
130.834
129.070
128.793
127.385
120.879
119.985
118.424
77.478
77.160
76.843
51.959
30.509
30.362
29.144
f1 (ppm)

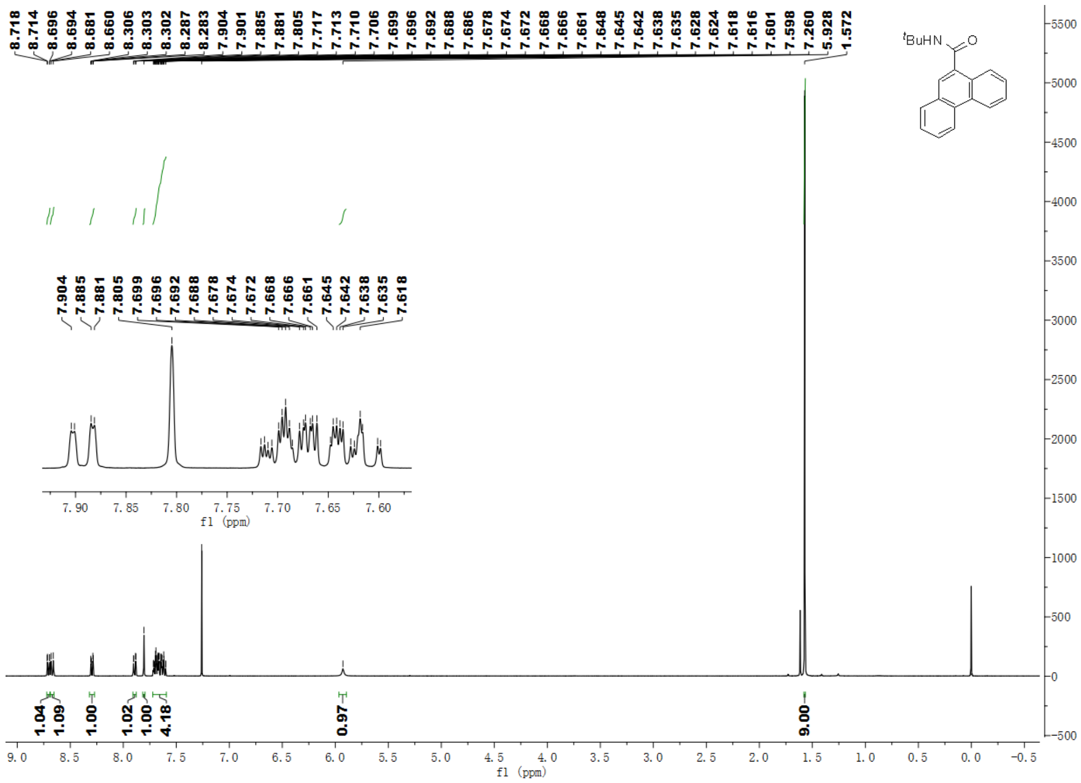

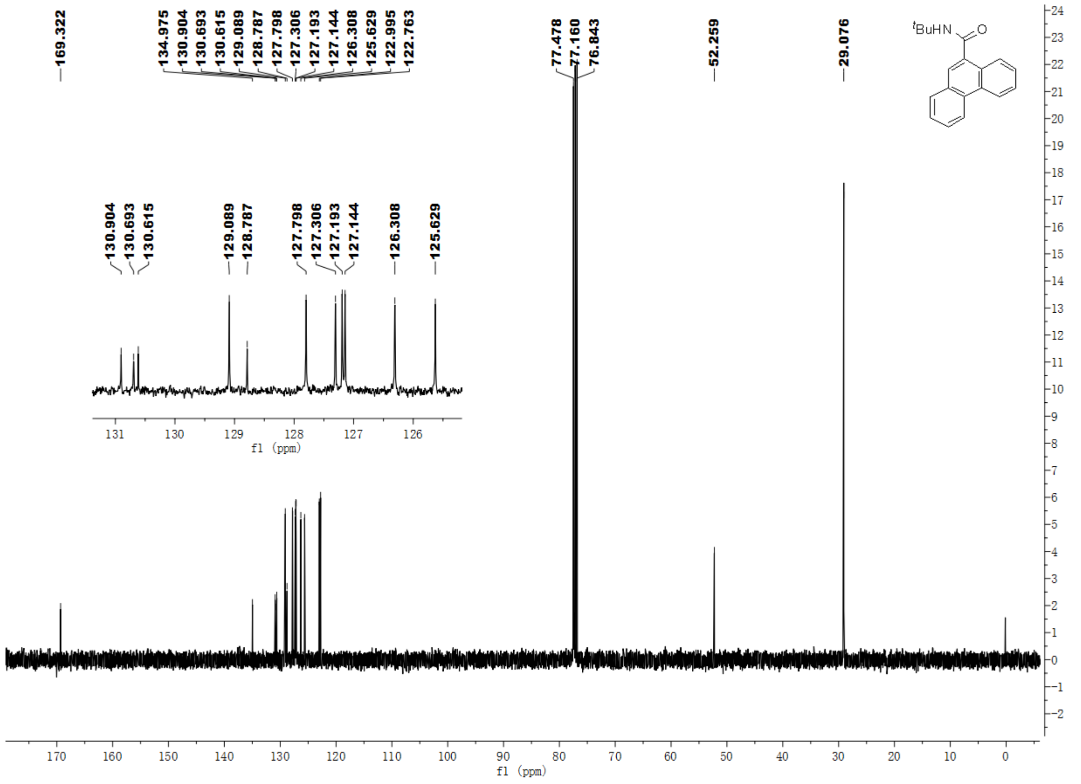

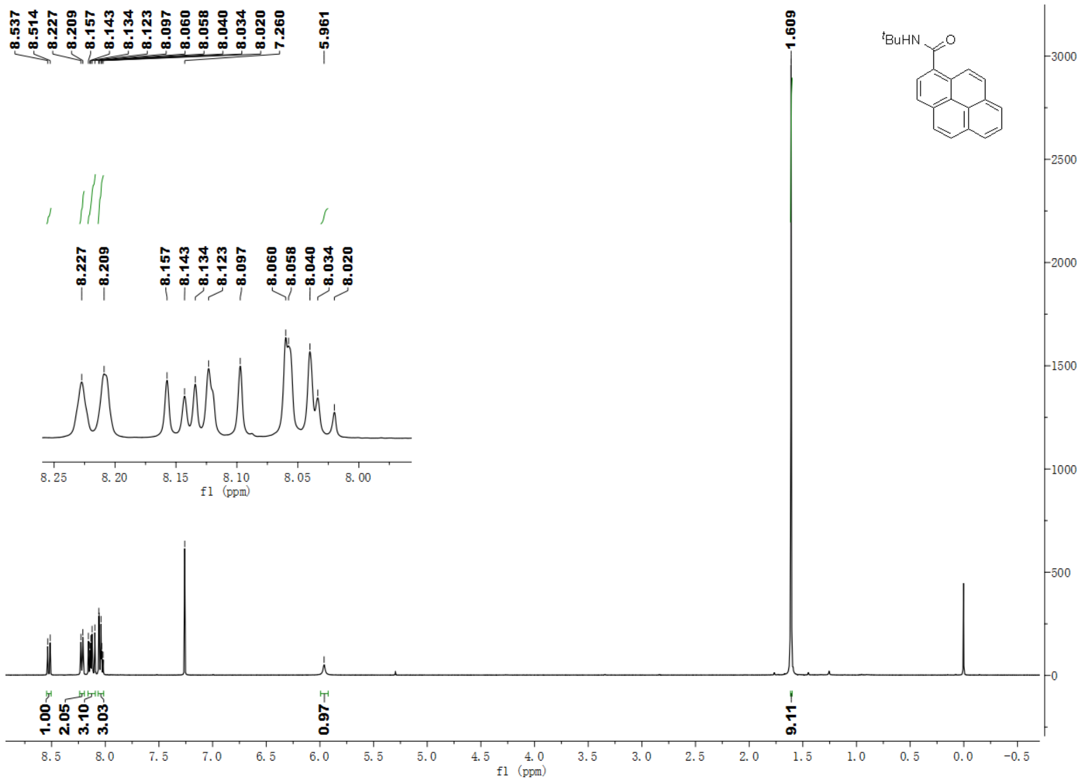
8.537
8.514
8.227
8.209
8.157
8.143
8.134
8.123
8.097
8.060
8.058
8.040
8.034
8.020
7.260
5.961
1.609
tBuHN
O
1.00
2.05
3.10
3.03
0.97
9.11
f1 (ppm)

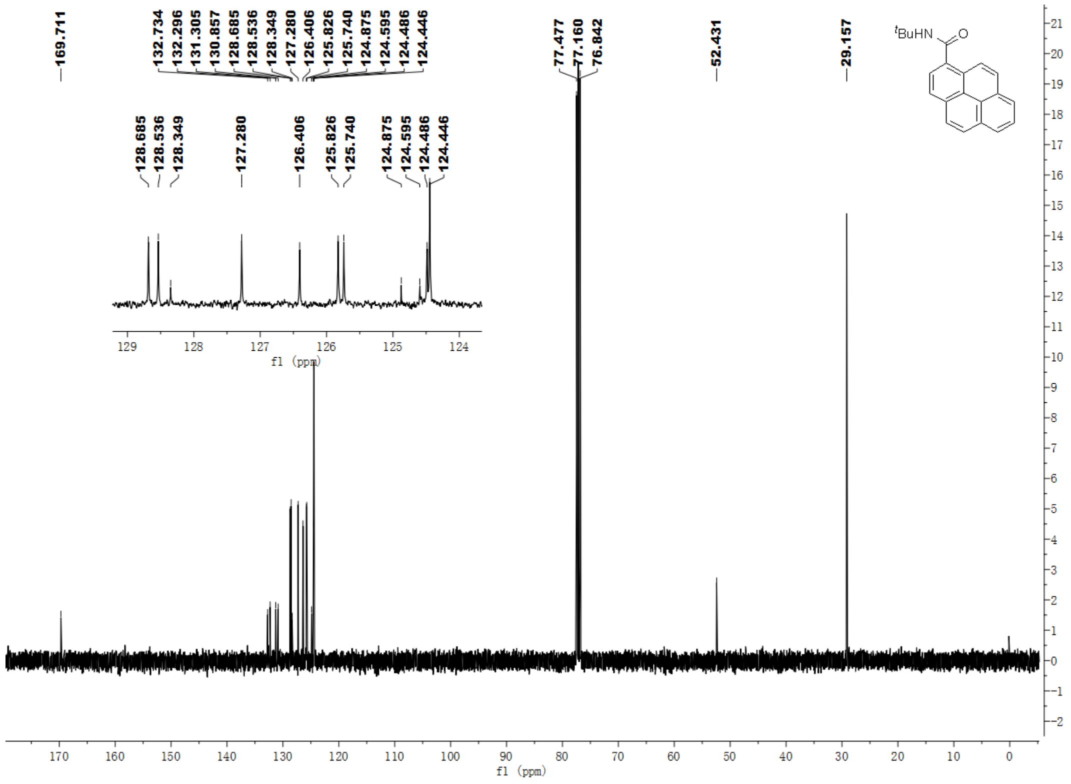
169.711
132.734
132.296
131.305
130.857
128.685
128.536
128.349
127.280
126.406
125.826
125.740
124.875
124.595
124.486
124.446
77.477
77.160
76.842
52.431
29.157
tBuHN
O
f1 (ppm)

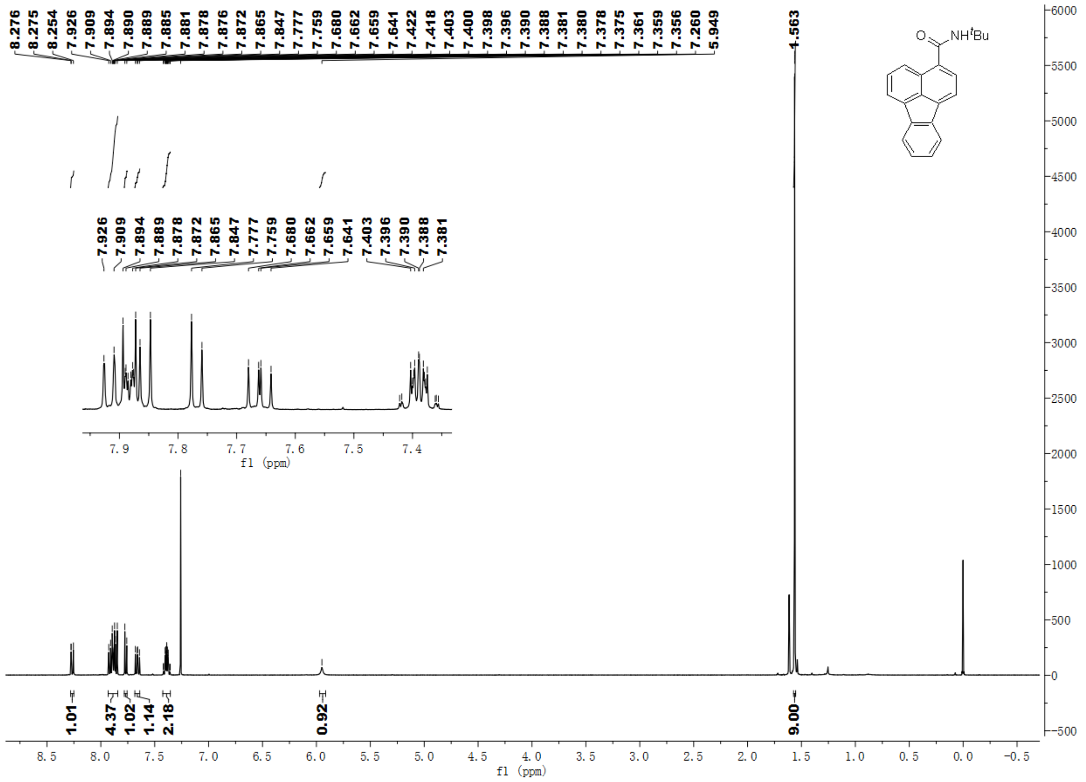

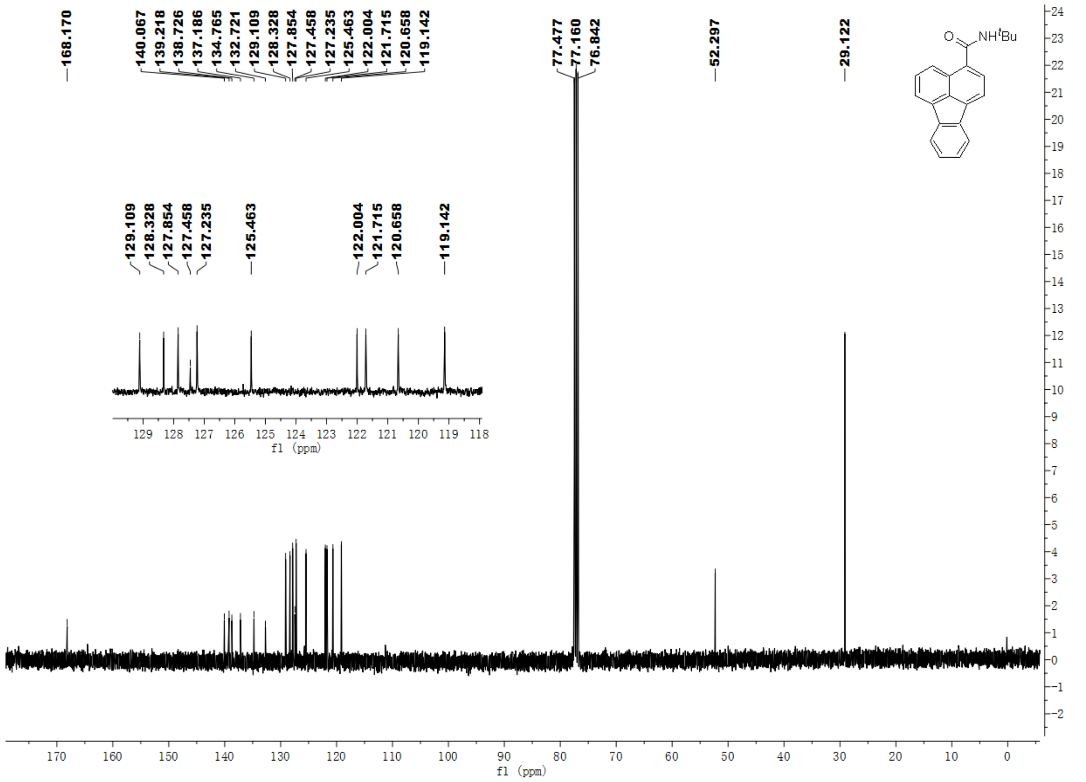

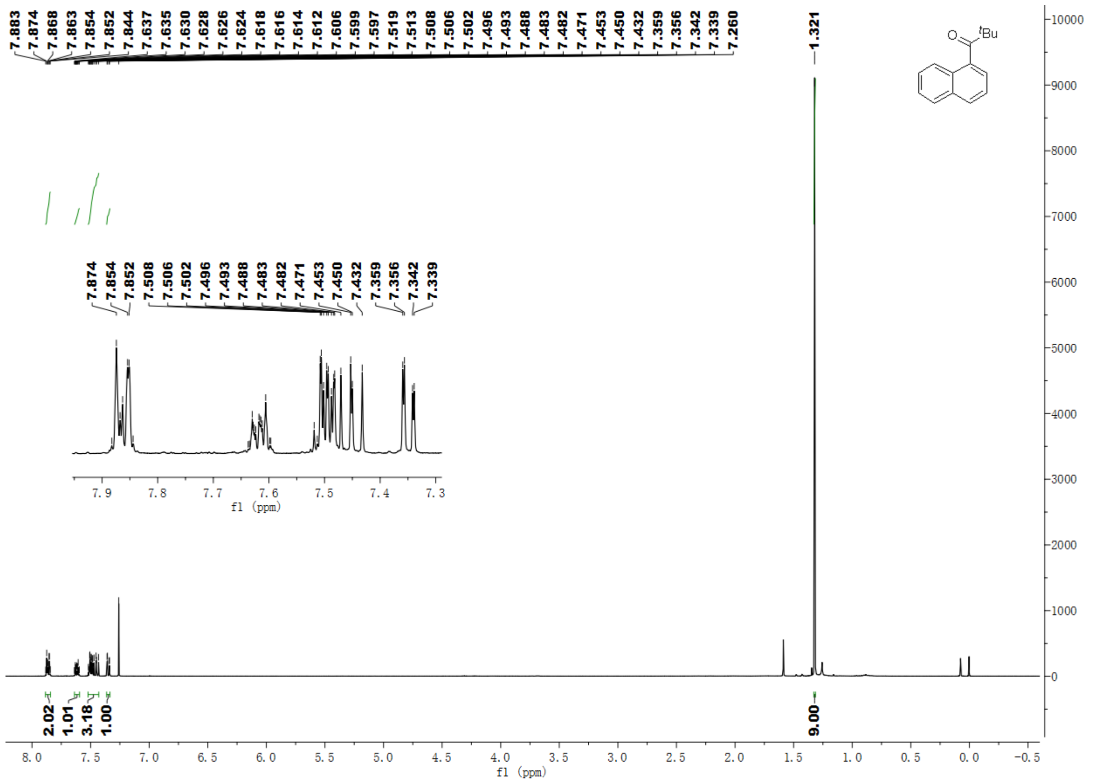
7.883
7.874
7.868
7.863
7.854
7.852
7.844
7.637
7.635
7.630
7.628
7.626
7.624
7.618
7.616
7.614
7.612
7.606
7.599
7.597
7.519
7.513
7.508
7.506
7.502
7.496
7.493
7.488
7.483
7.482
7.471
7.453
7.450
7.432
7.359
7.356
7.342
7.339
7.260
1.321
O
tBu
2.02
1.01
3.18
1.00
9.00
f1 (ppm)

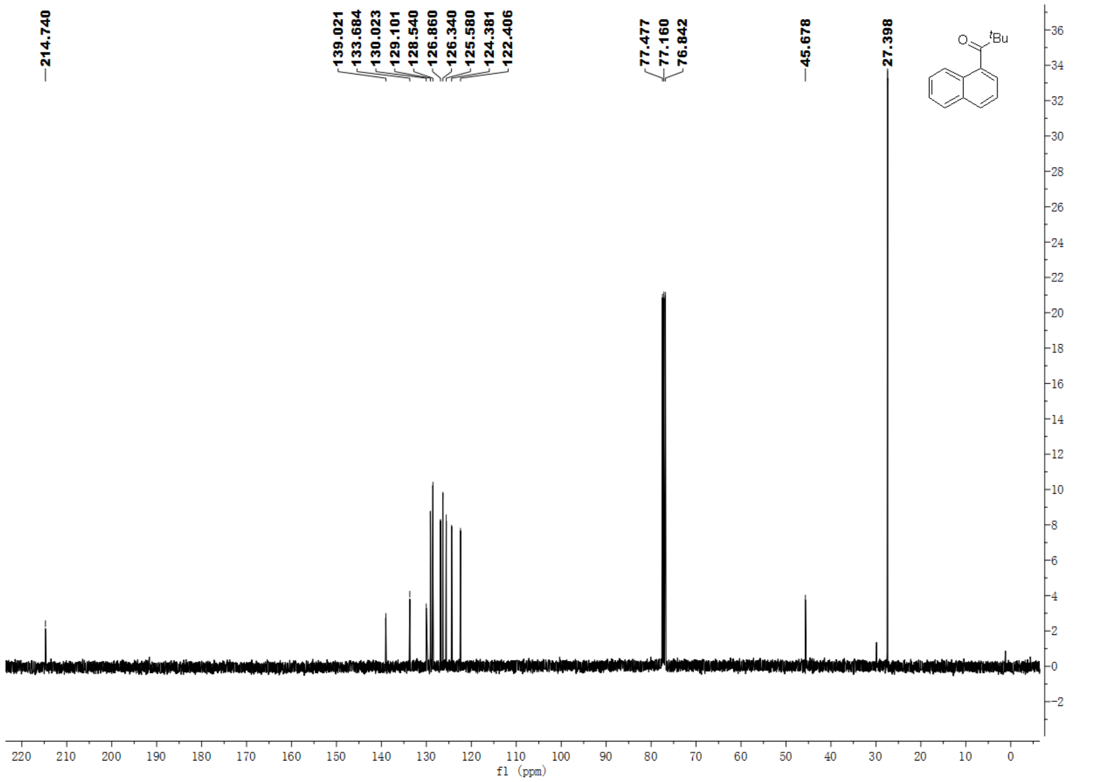
214.740
139.021
133.684
130.023
129.101
128.540
126.860
126.340
125.580
124.381
122.406
77.477
77.160
76.842
45.678
27.398
O
tBu
f1 (ppm)

^{1}H and ^{13}C NMR Spectra of Products

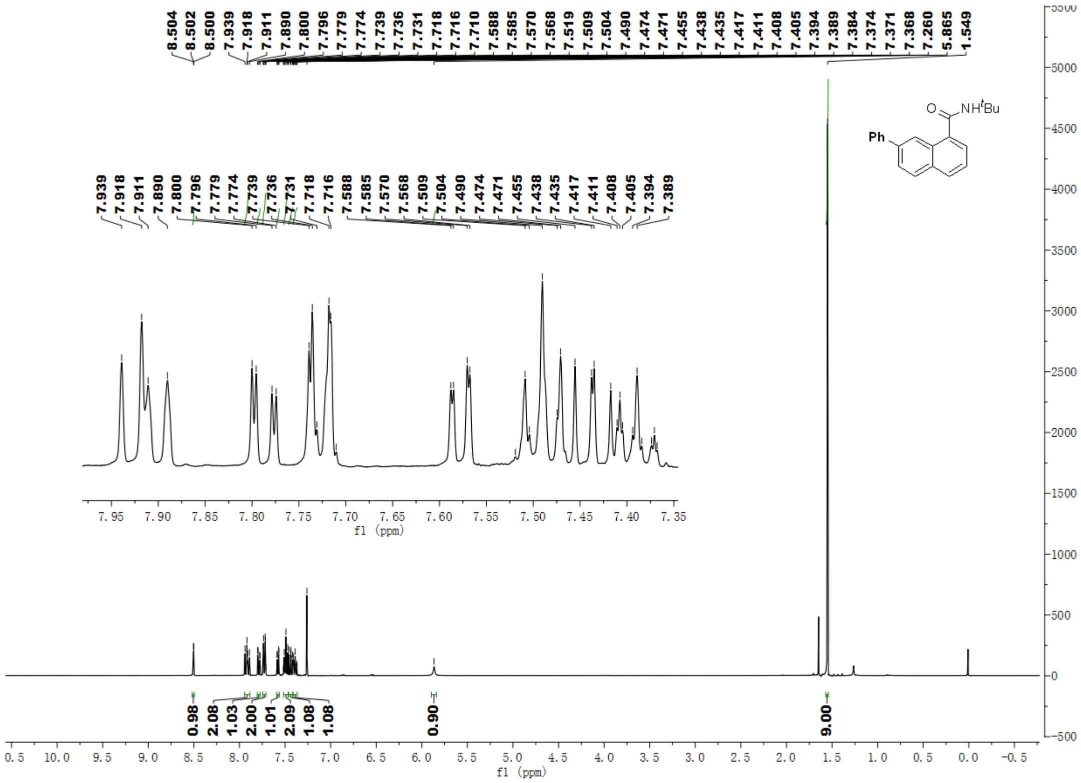

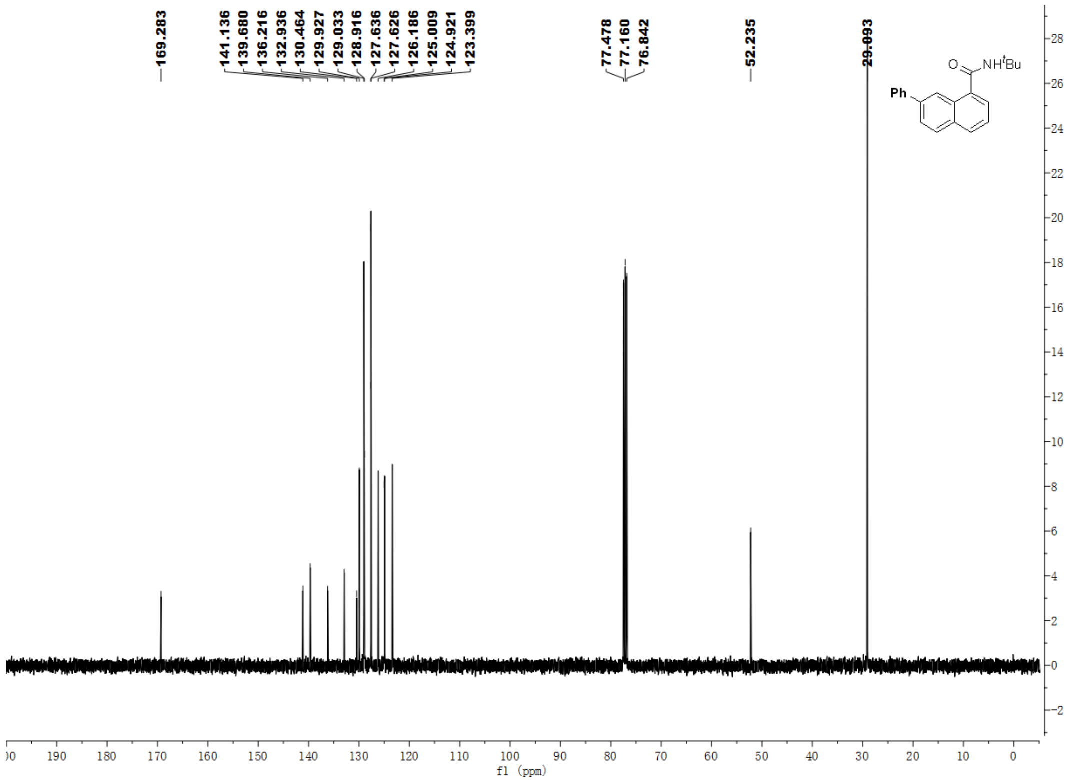

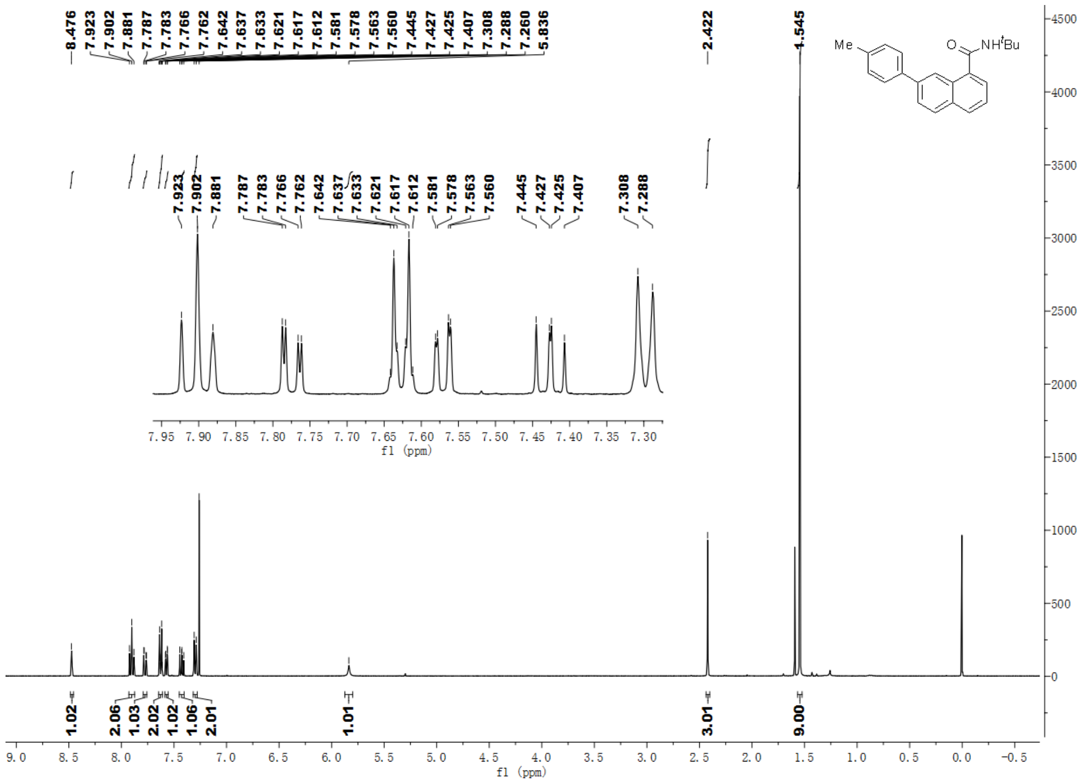
8.476
7.923
7.902
7.881
7.787
7.783
7.766
7.762
7.642
7.637
7.633
7.621
7.617
7.612
7.581
7.578
7.563
7.560
7.445
7.427
7.425
7.407
7.308
7.288
7.260
5.836
2.422
1.545
Me
O
NH^{t}Bu
1.02
2.06
1.03
2.02
1.02
1.06
2.01
1.01
3.01
9.00
f1 (ppm)

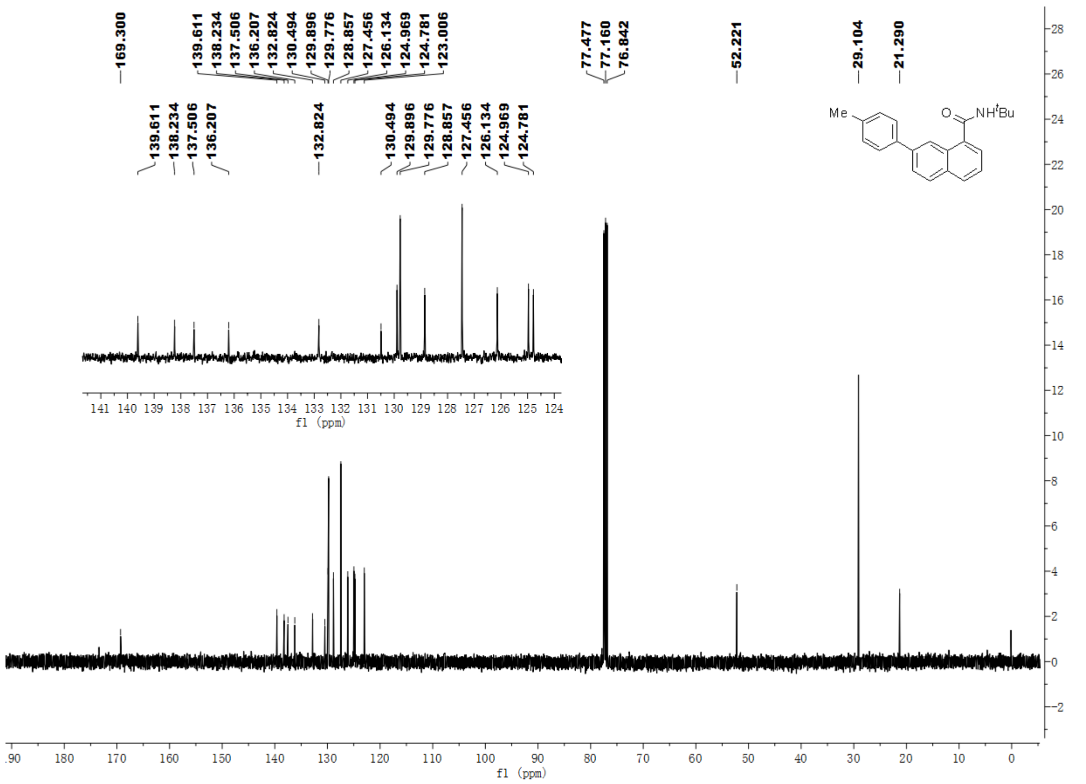
169.300
139.611
138.234
137.506
136.207
132.824
130.494
129.896
129.776
128.857
127.456
126.134
124.969
124.781
123.006
77.477
77.160
76.842
52.221
29.104
21.290
Me
O
NH^{t}Bu
f1 (ppm)

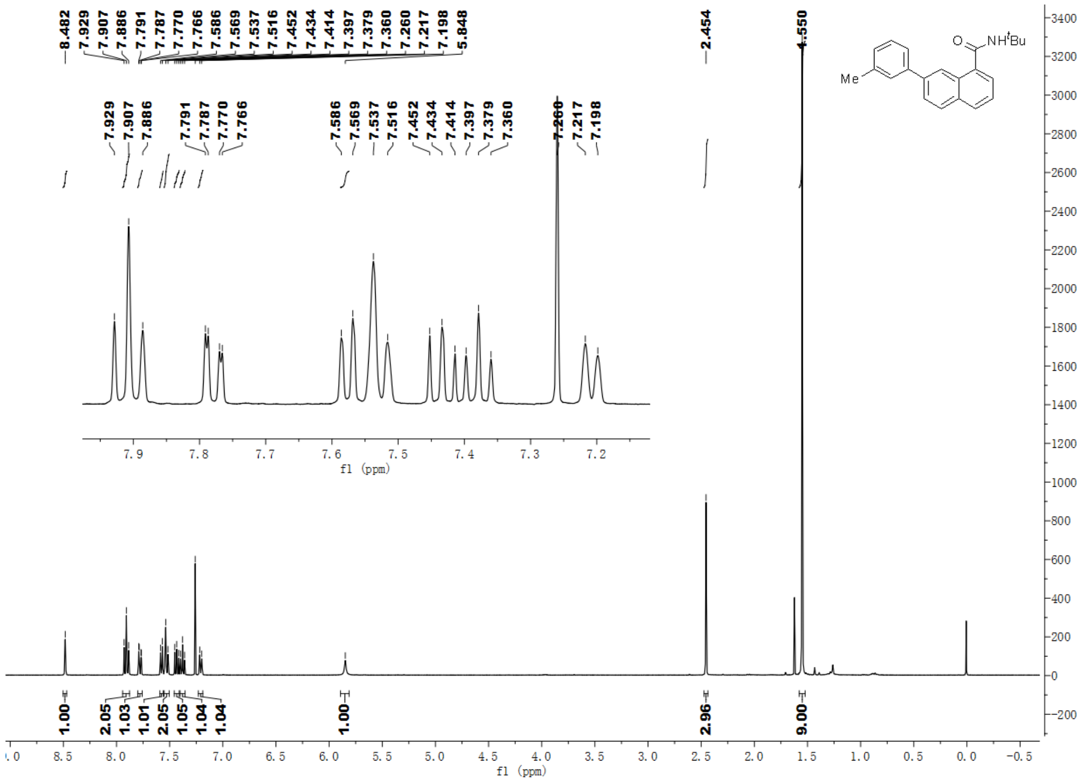

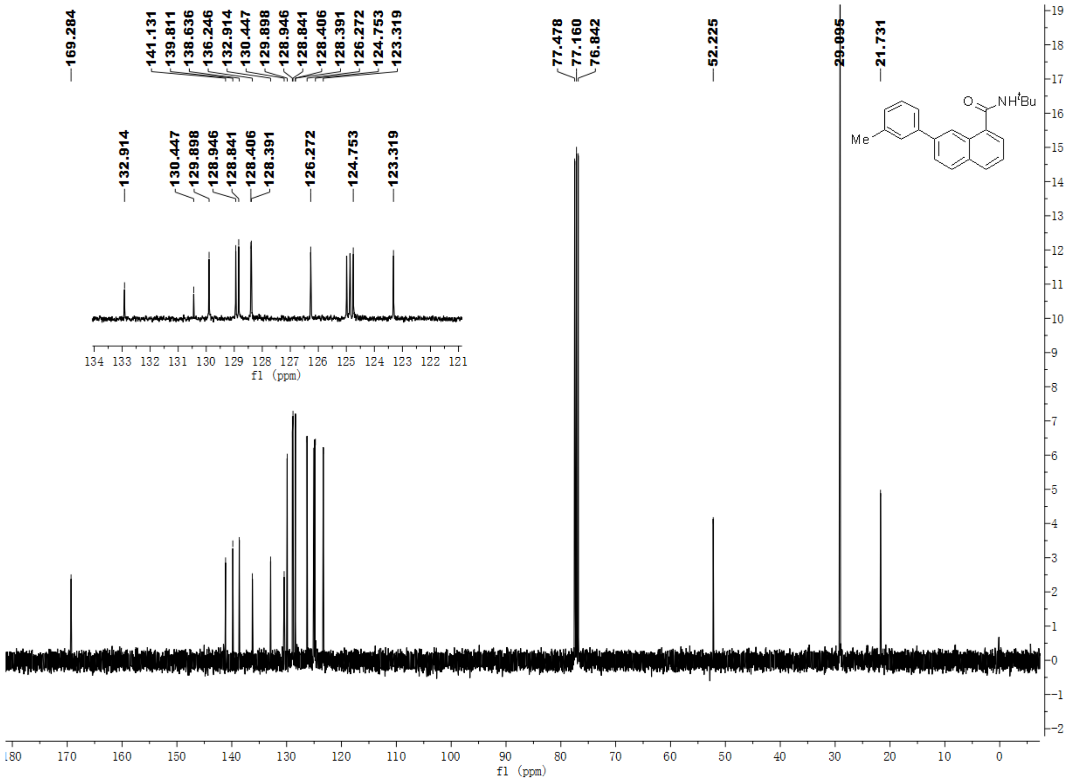

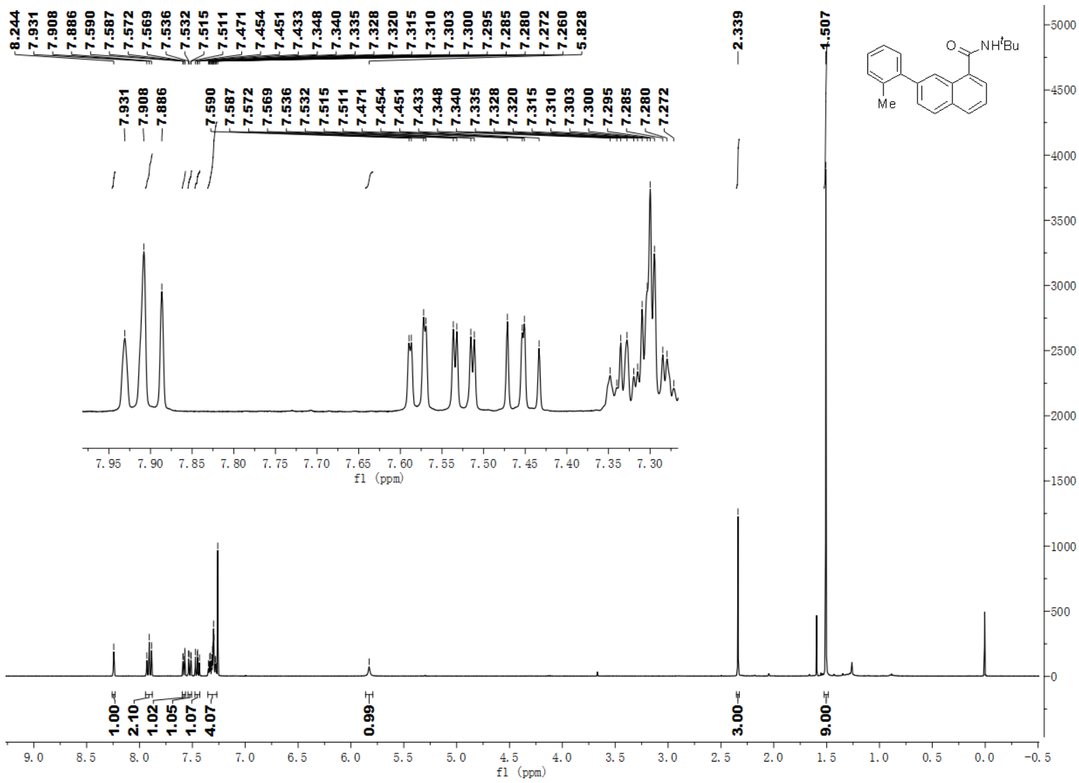

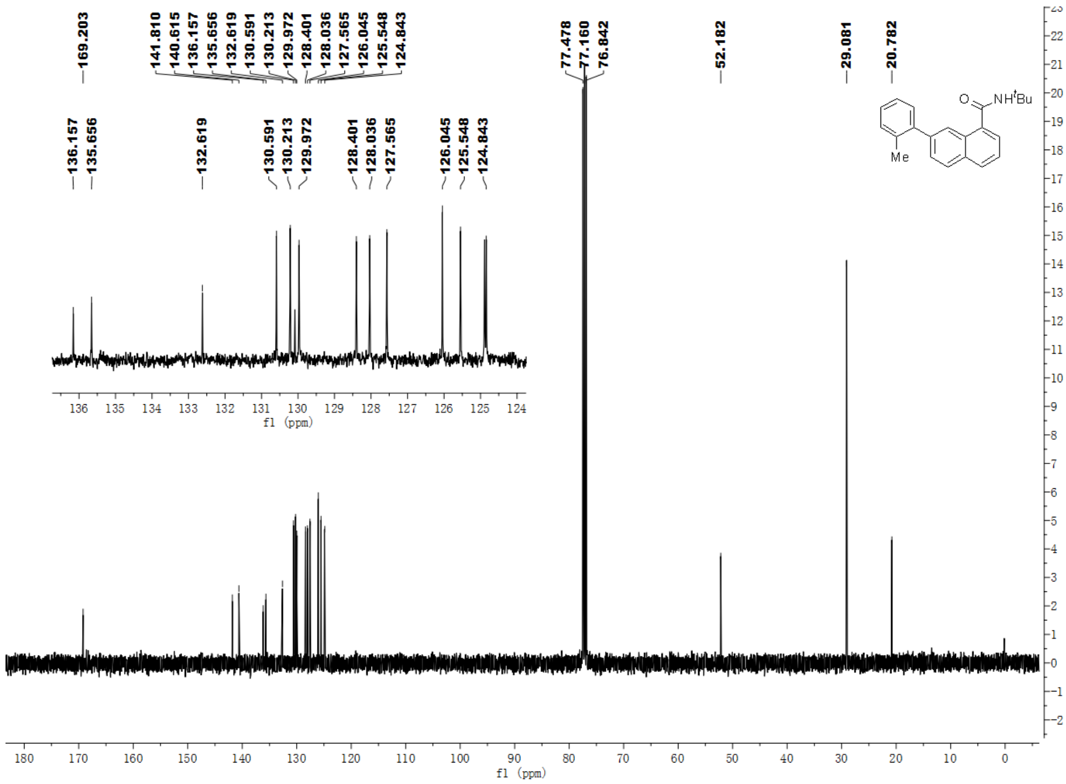

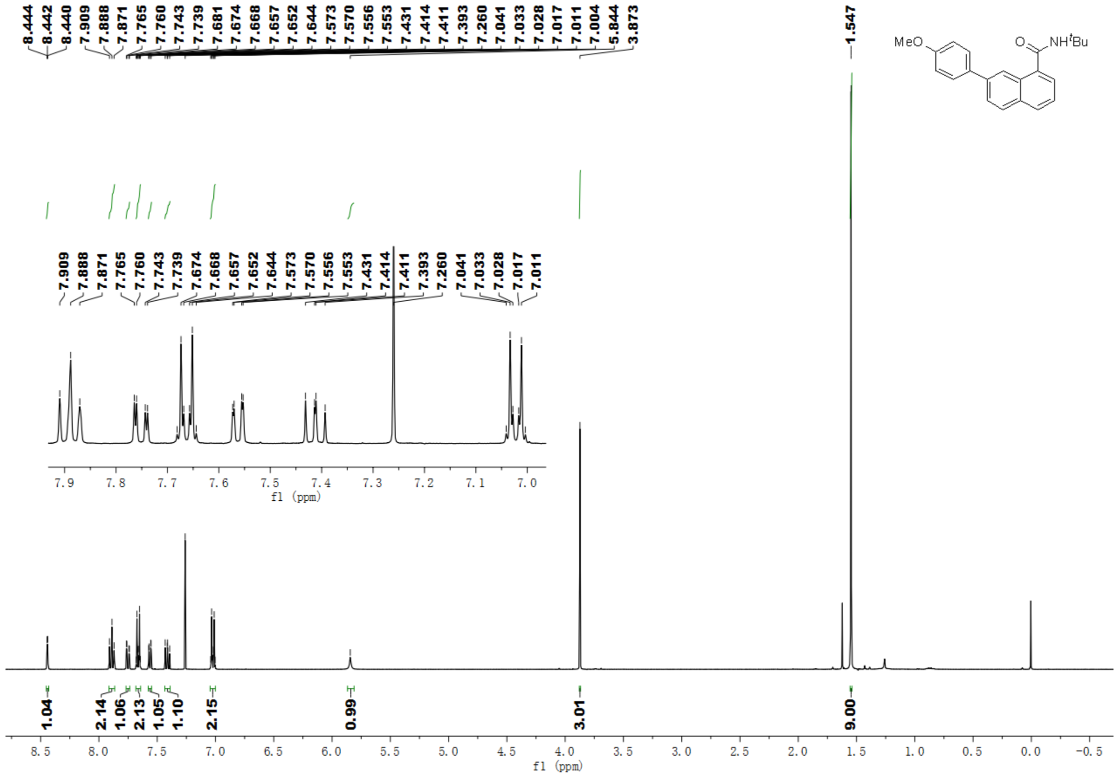

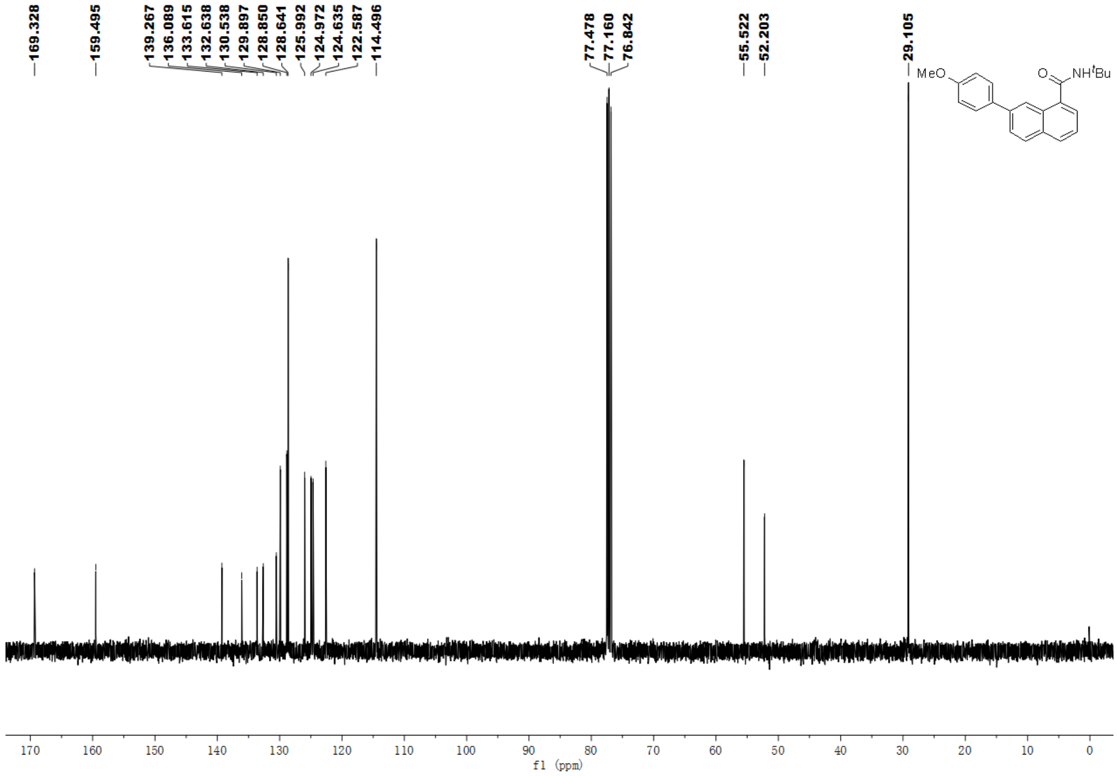

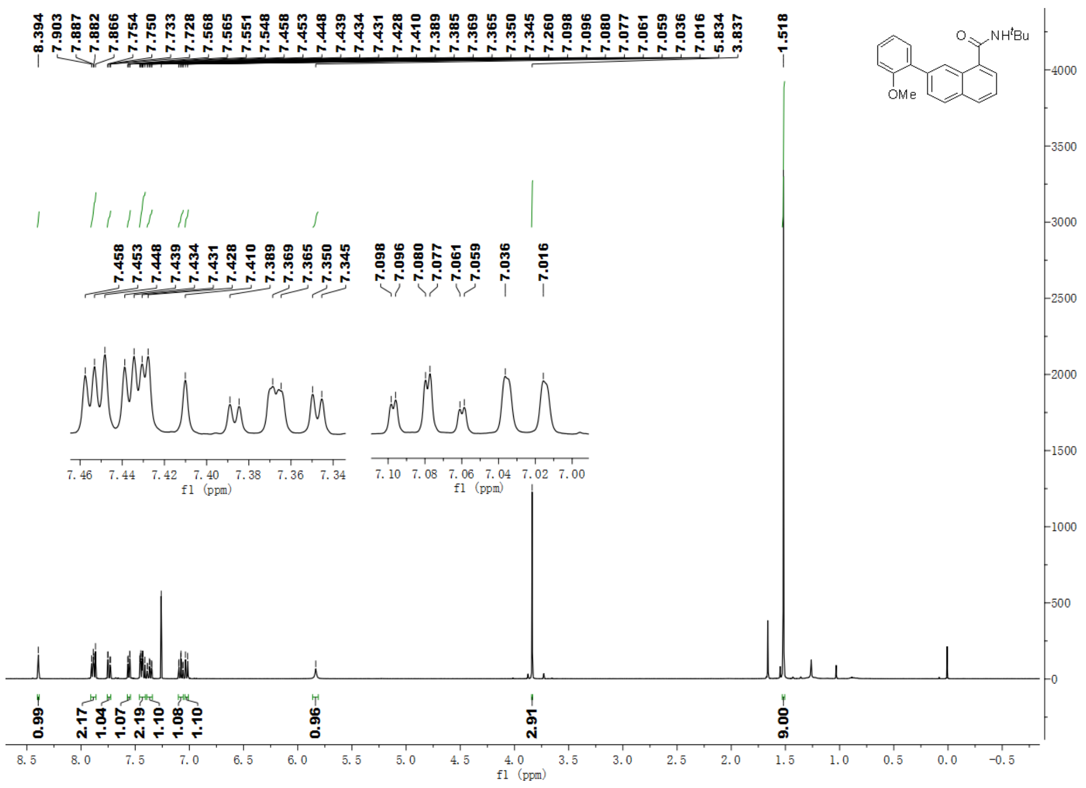

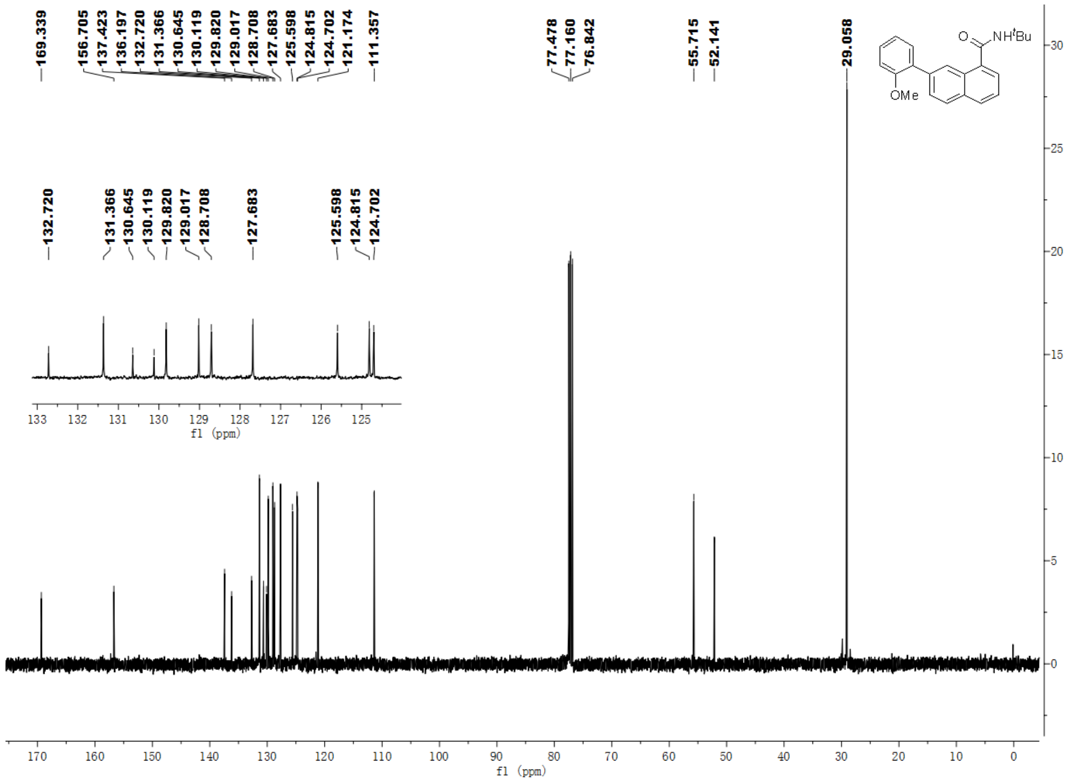

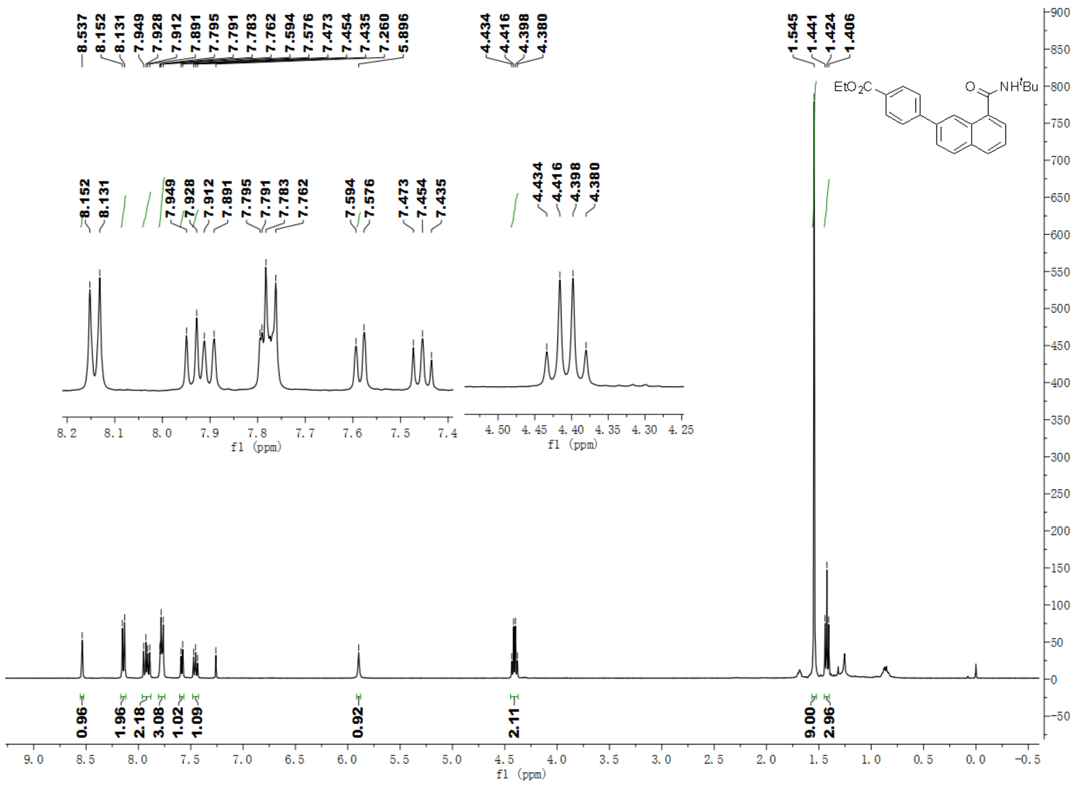

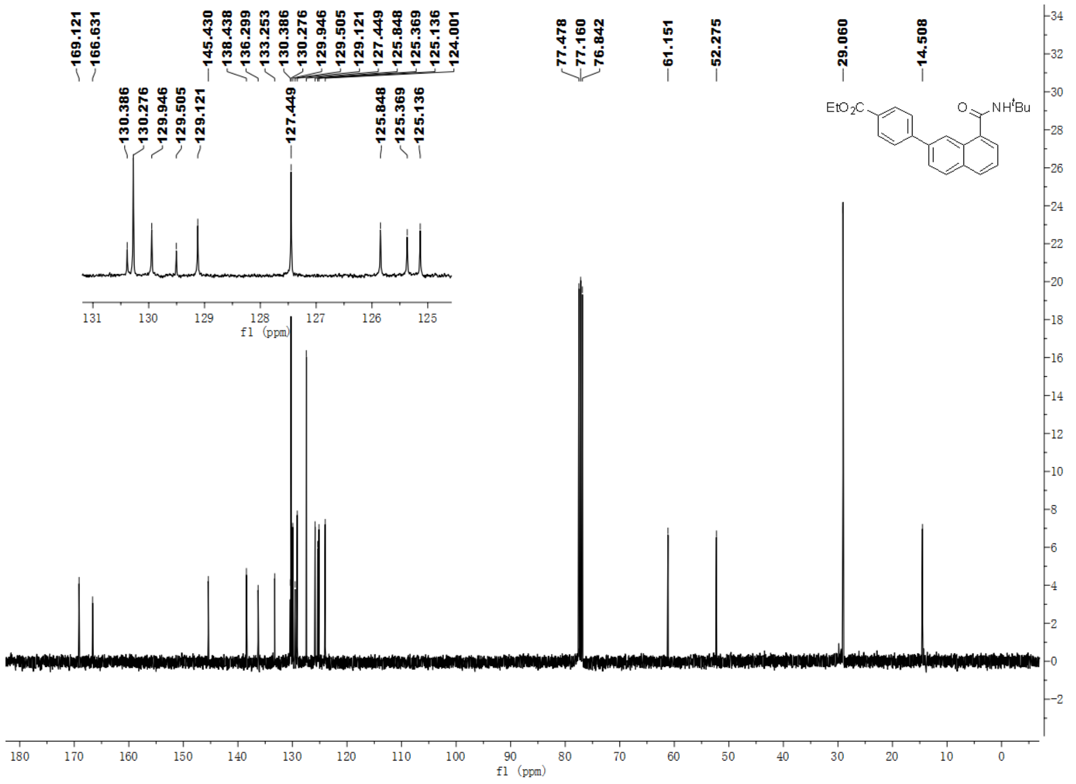

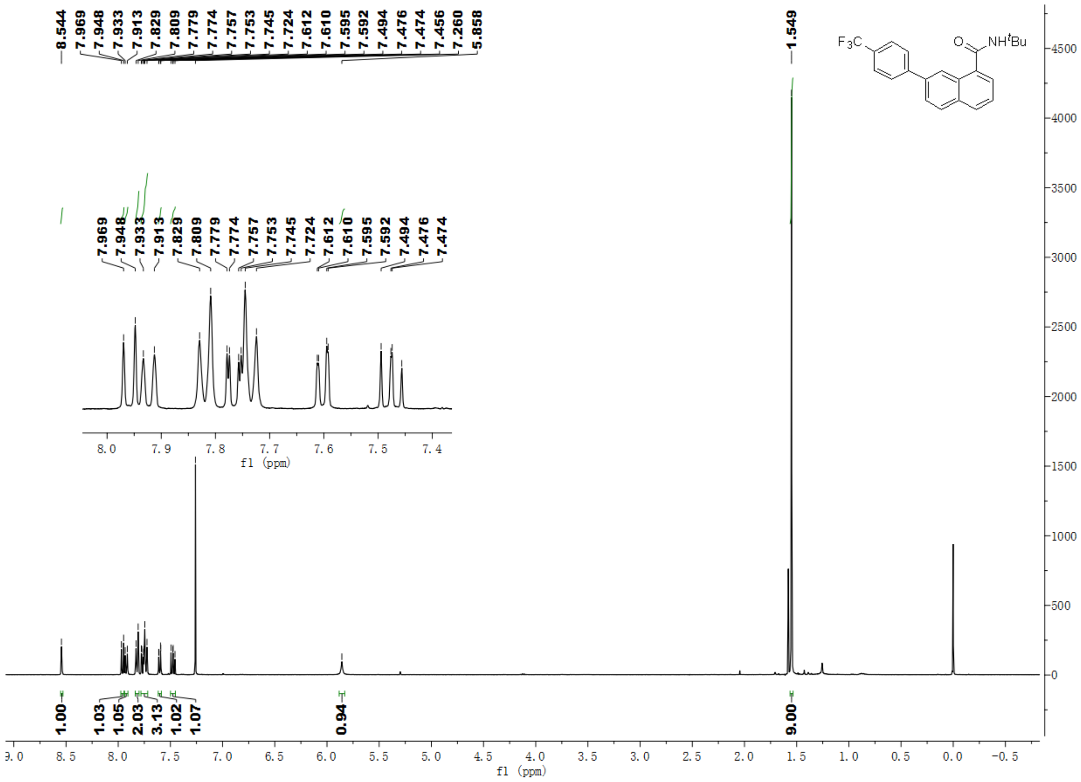

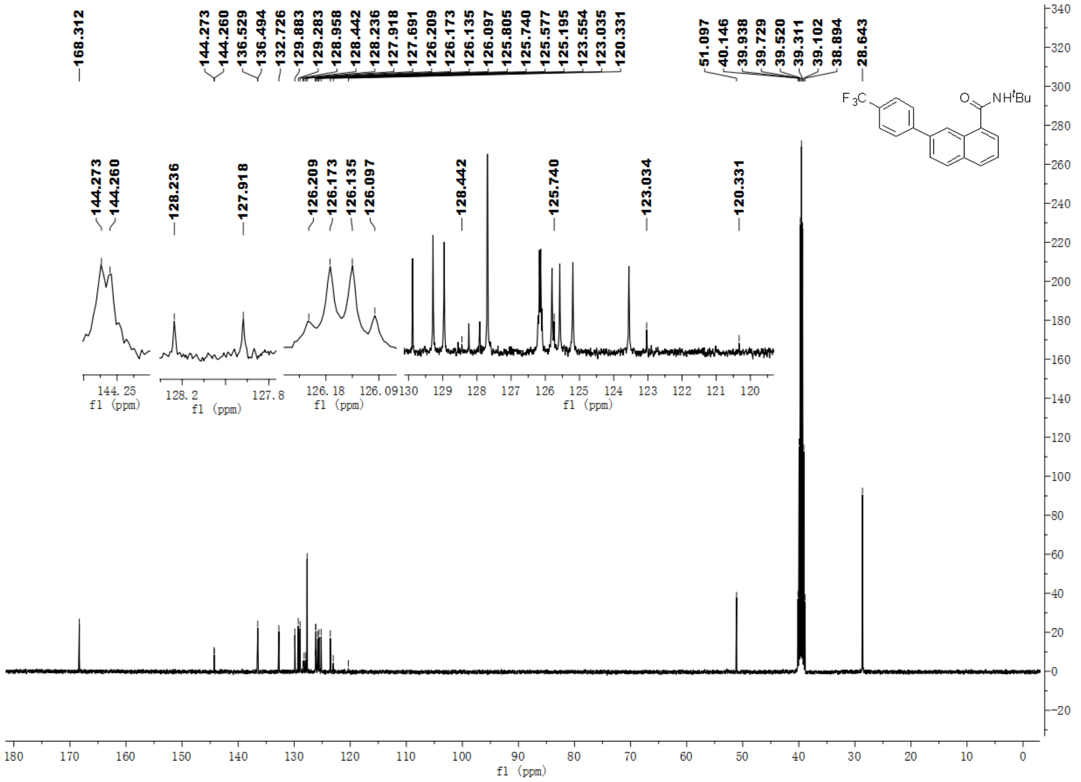

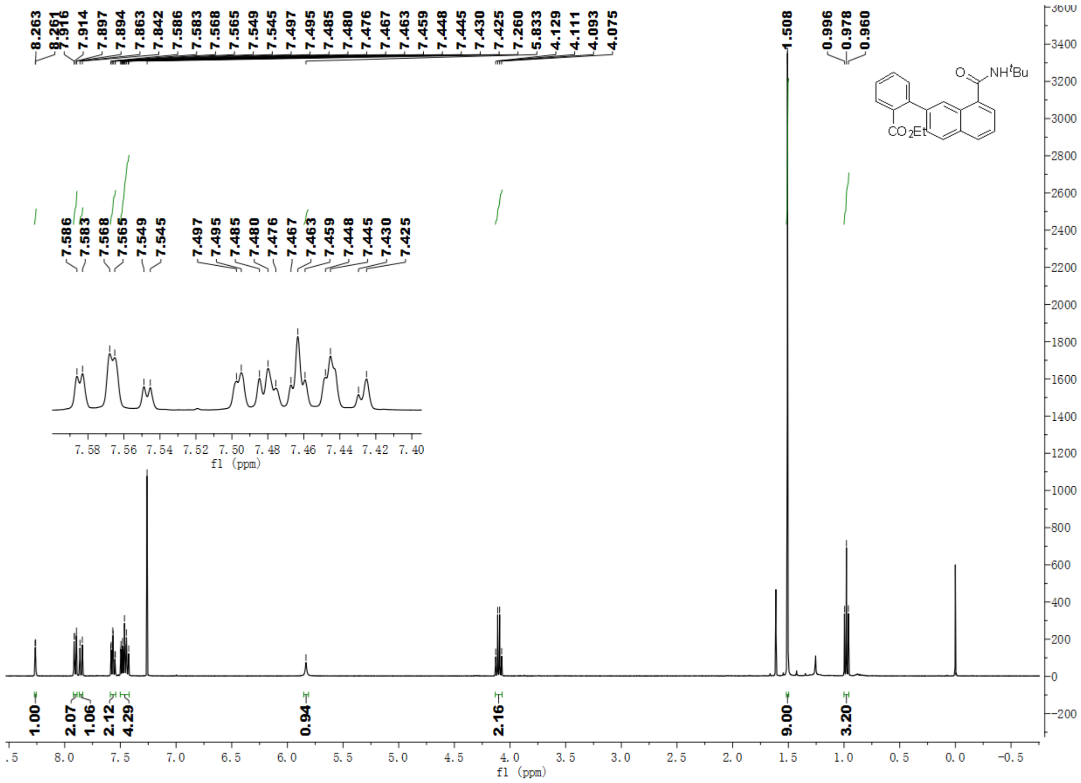

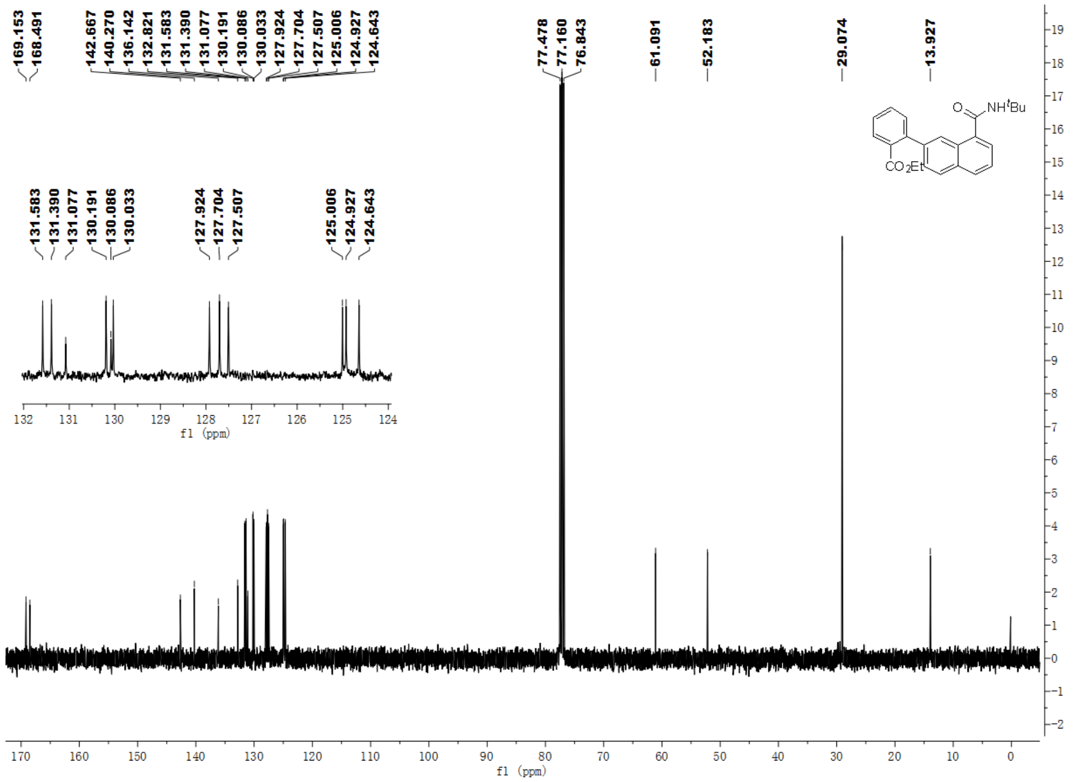

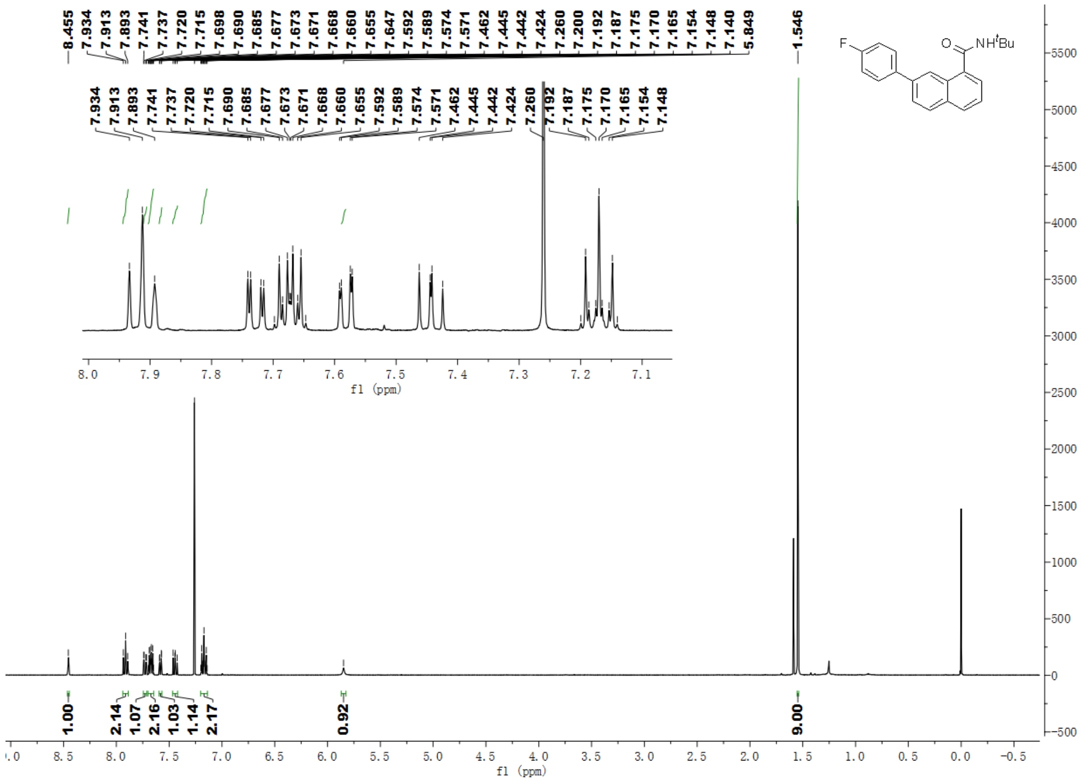
8.455
7.934
7.913
7.893
7.741
7.737
7.720
7.715
7.698
7.690
7.685
7.677
7.673
7.671
7.668
7.660
7.655
7.647
7.592
7.589
7.574
7.571
7.462
7.445
7.442
7.424
7.260
7.200
7.192
7.187
7.175
7.170
7.165
7.154
7.148
7.140
5.849
1.546
F
O
NH^{t}Bu
1.00
2.14
1.07
2.16
1.03
1.14
2.17
0.92
9.00
f1 (ppm)

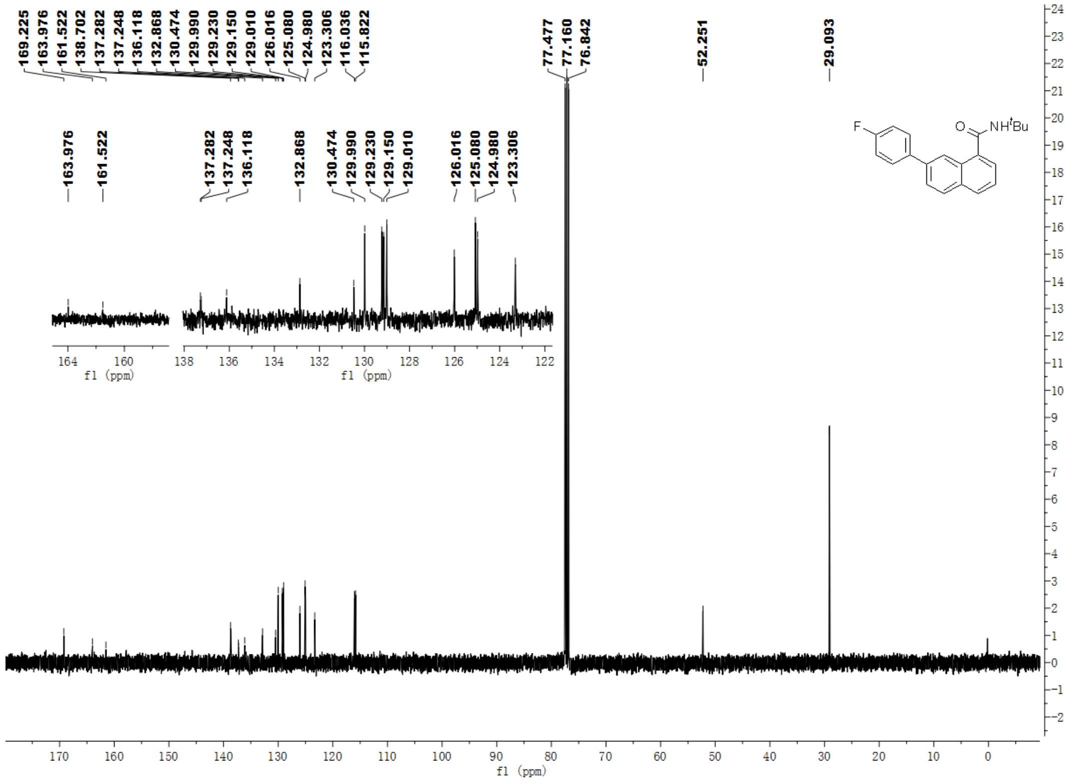
169.225
163.976
161.522
138.702
137.282
137.248
136.118
132.868
130.474
129.990
129.230
129.150
129.010
126.016
125.080
124.980
123.306
116.036
115.822
77.477
77.160
76.842
52.251
29.093
F
O
NH^{t}Bu
f1 (ppm)

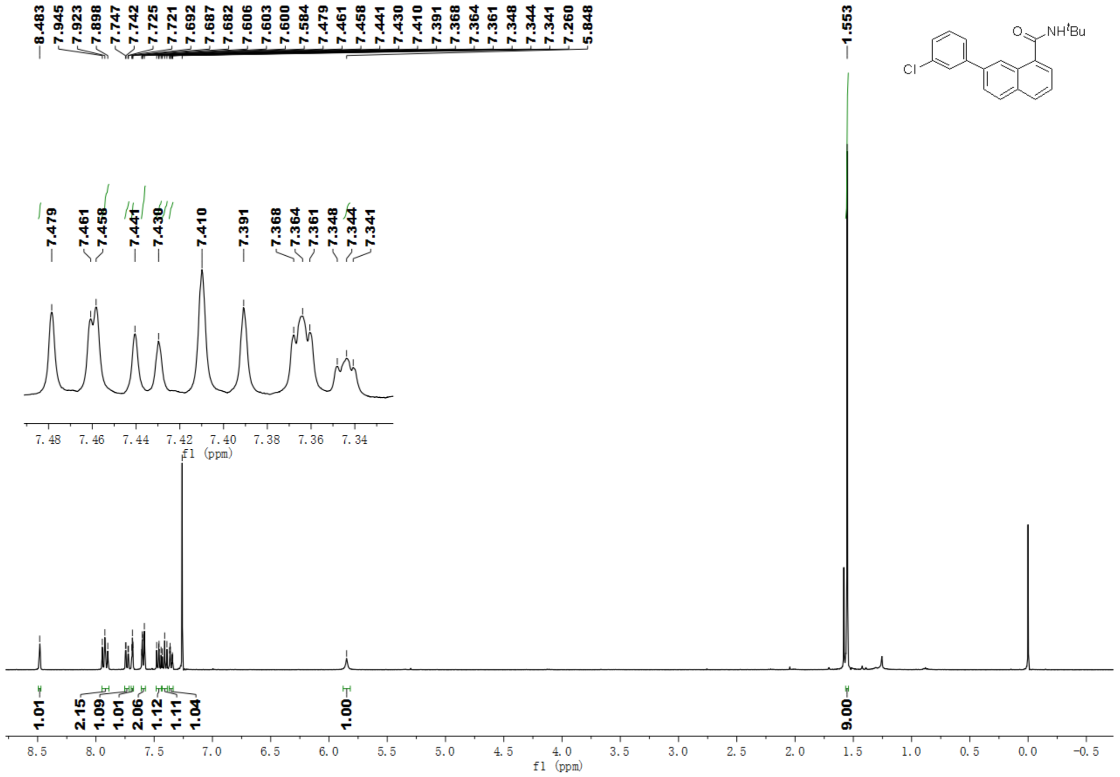

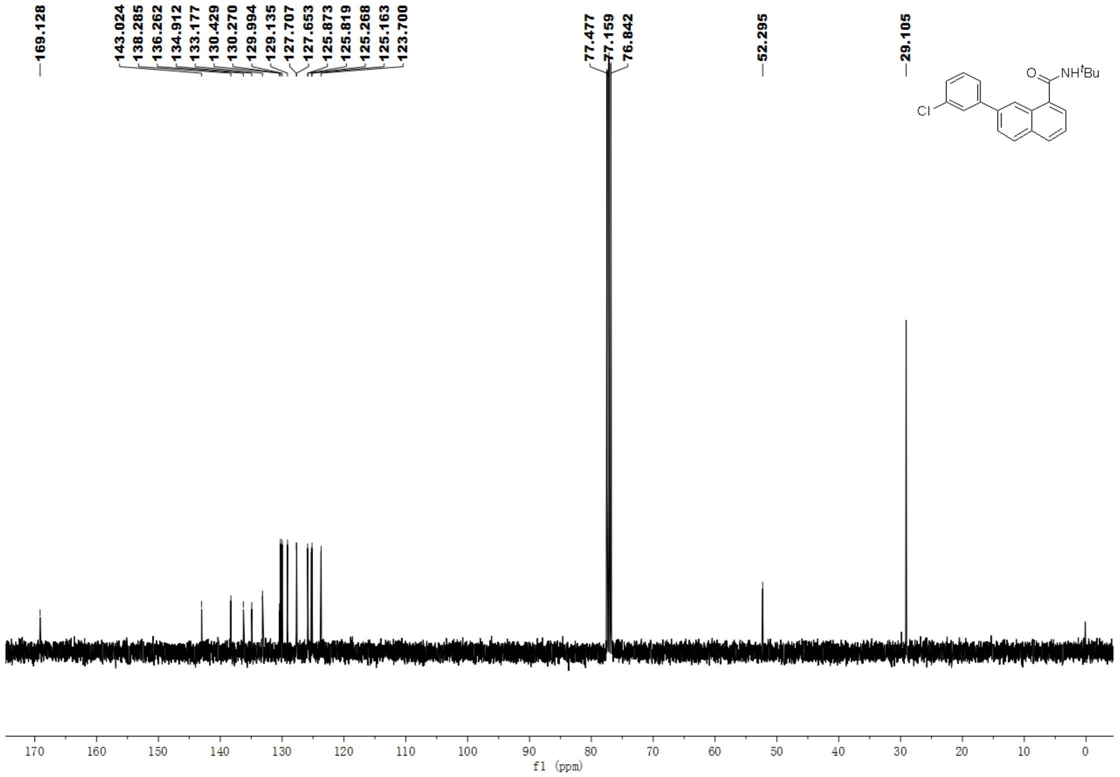

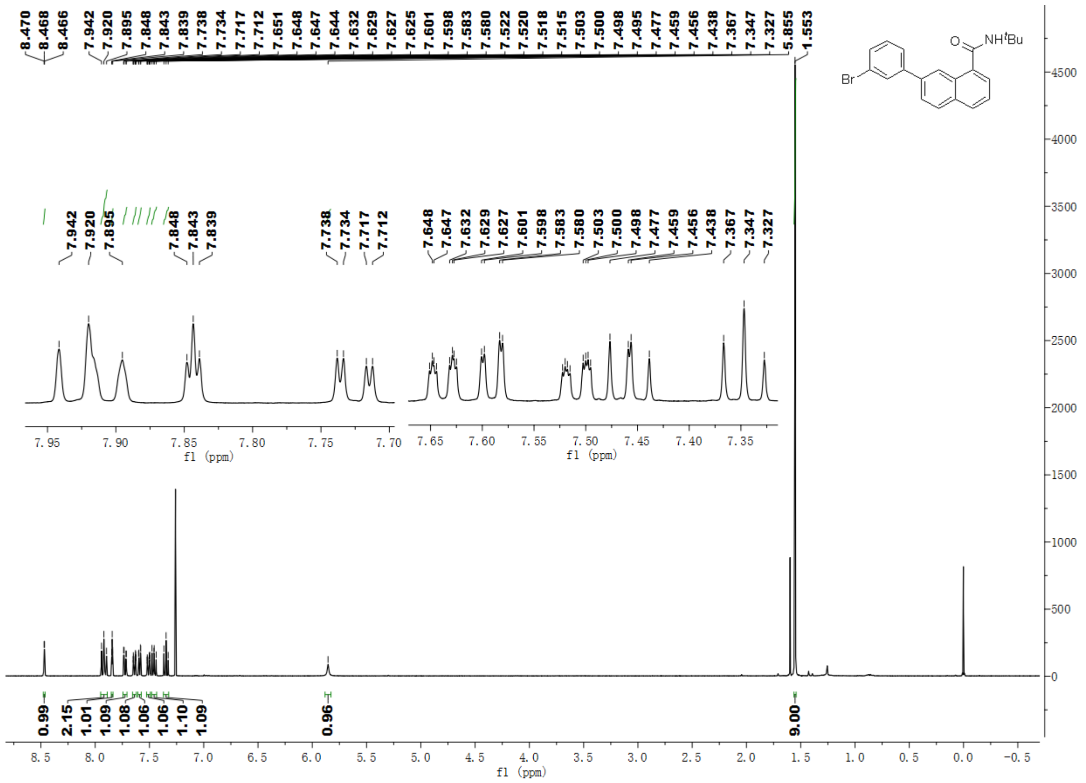

8.470
8.468
8.466
7.942
7.920
7.895
7.848
7.843
7.839
7.738
7.734
7.717
7.712
7.651
7.648
7.647
7.644
7.632
7.629
7.627
7.625
7.601
7.598
7.583
7.580
7.522
7.520
7.518
7.515
7.503
7.500
7.498
7.495
7.477
7.459
7.456
7.438
7.367
7.347
7.327
5.855
1.553
O
NH^{t}Bu
Br
0.99
2.15
1.01
1.09
1.08
1.06
1.06
1.10
1.09
0.96
9.00
f1 (ppm)

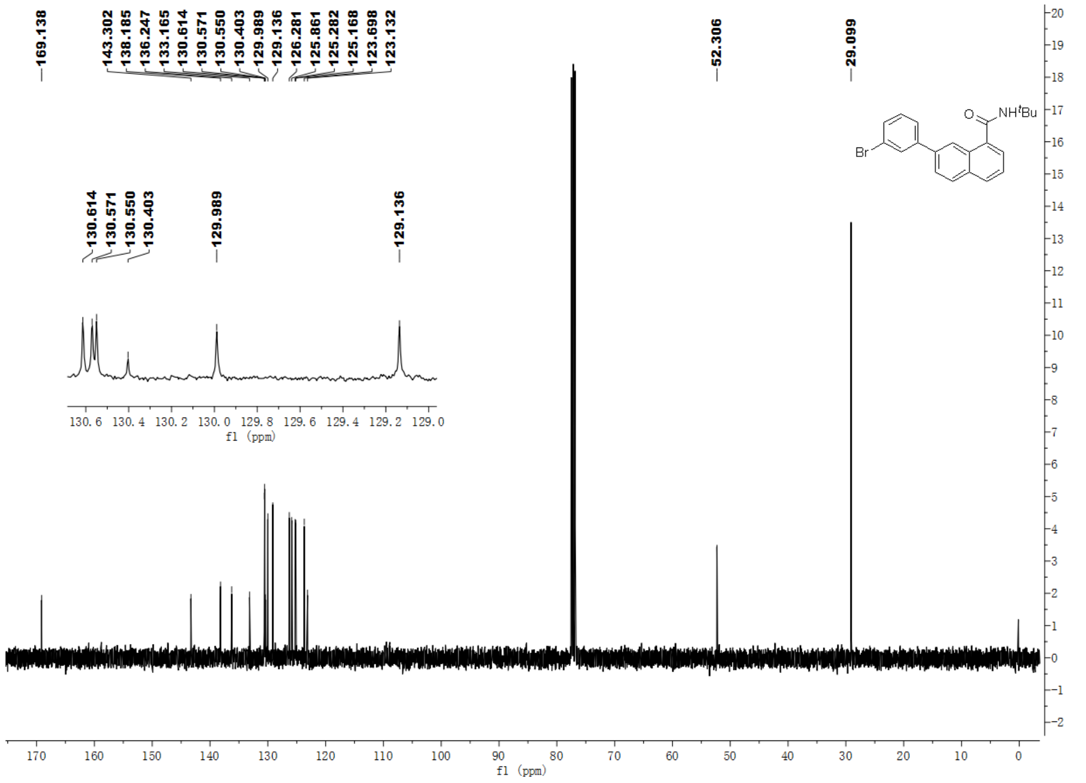

169.138
143.302
138.185
136.247
133.165
130.614
130.571
130.550
130.403
129.989
129.136
126.281
125.861
125.282
125.168
123.698
123.132
52.306
29.099
O
NH^{t}Bu
Br
f1 (ppm)

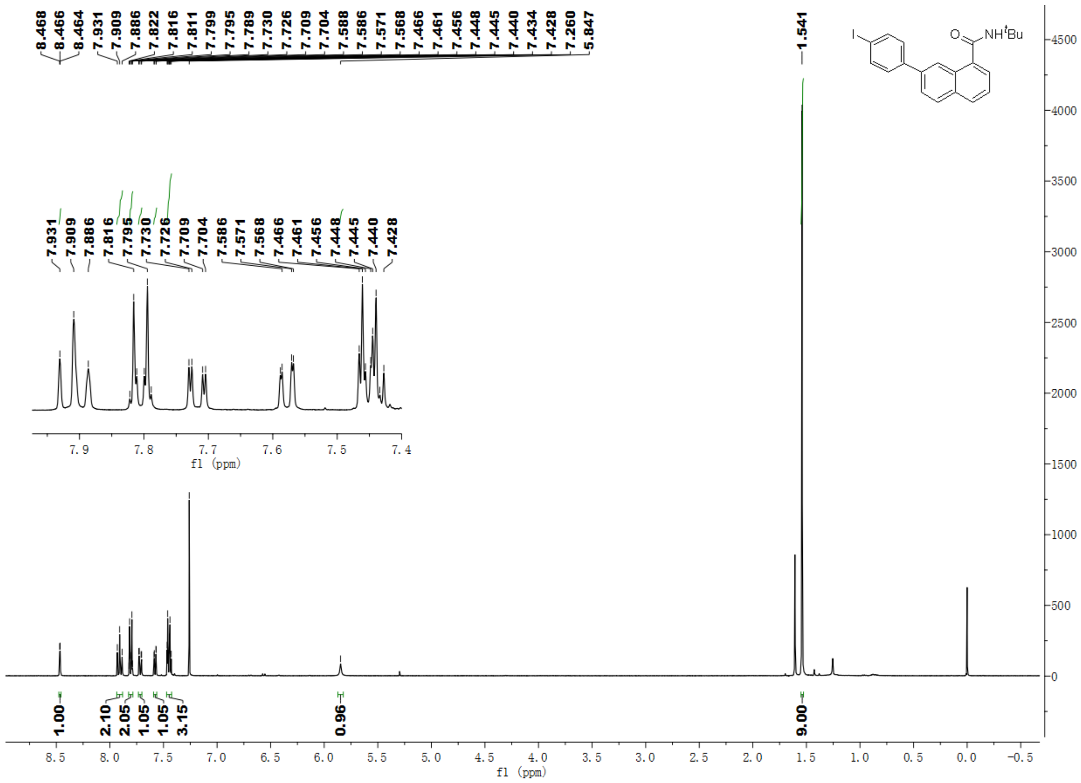

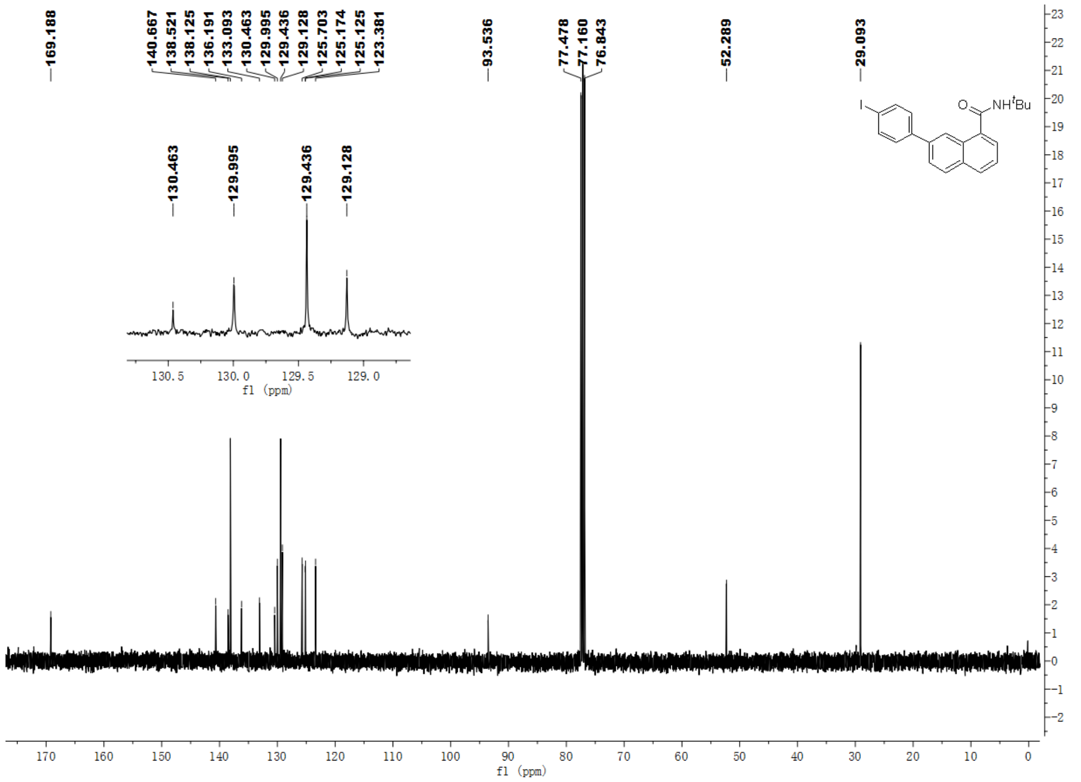

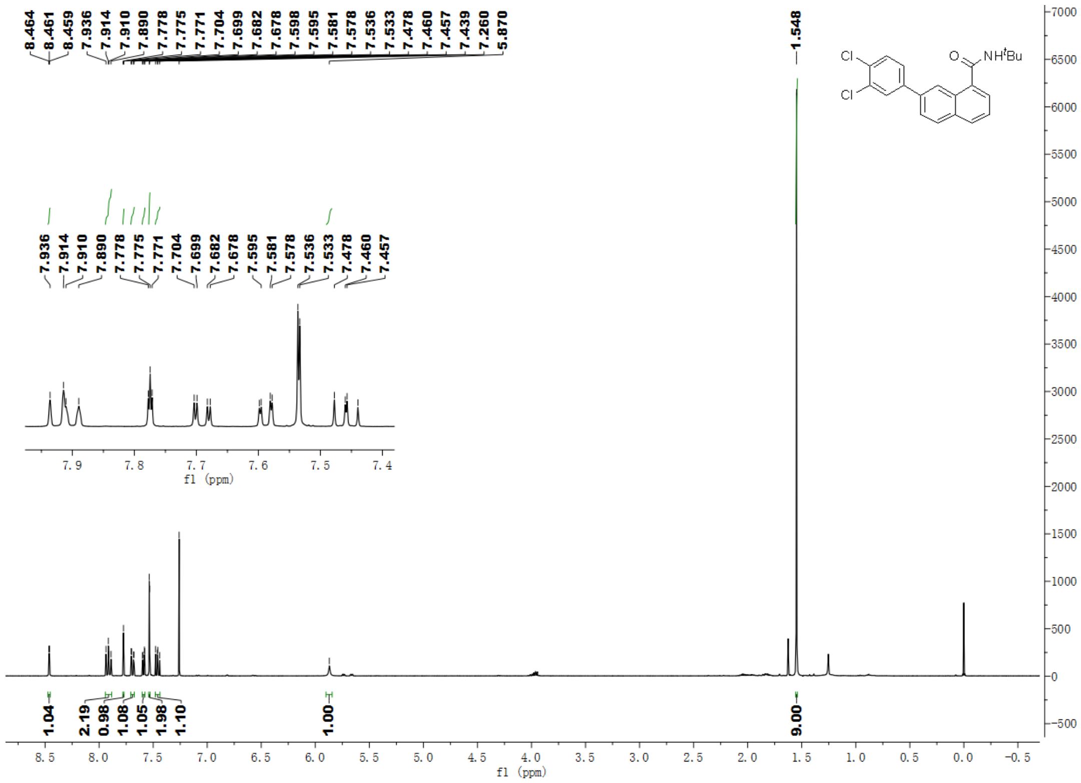
8.464
8.461
8.459
7.936
7.914
7.910
7.890
7.778
7.775
7.771
7.704
7.699
7.682
7.678
7.598
7.595
7.581
7.578
7.536
7.533
7.478
7.460
7.457
7.439
7.260
5.870
1.548
Cl
Cl
O
NH^{t}Bu
1.04
2.19
0.98
1.08
1.05
1.98
1.10
1.00
9.00
f1 (ppm)

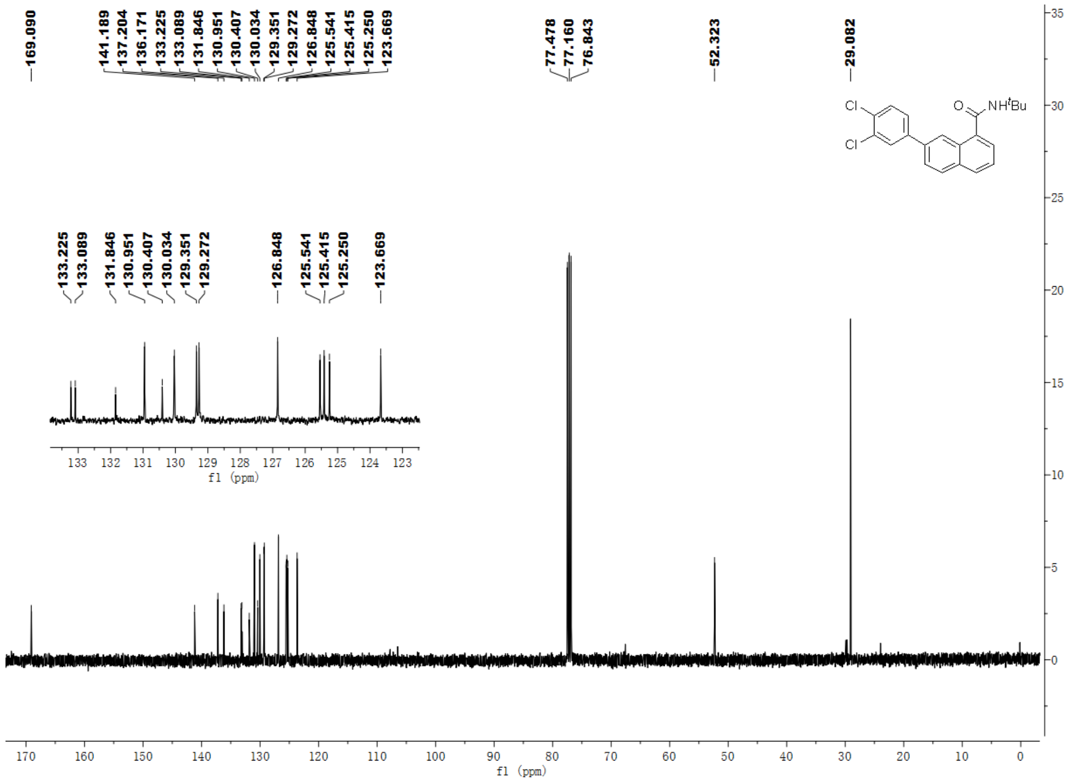
169.090
141.189
137.204
136.171
133.225
133.089
131.846
130.951
130.407
130.034
129.351
129.272
126.848
125.541
125.415
125.250
123.669
77.478
77.160
76.843
52.323
29.082
Cl
Cl
O
NH^{t}Bu
f1 (ppm)

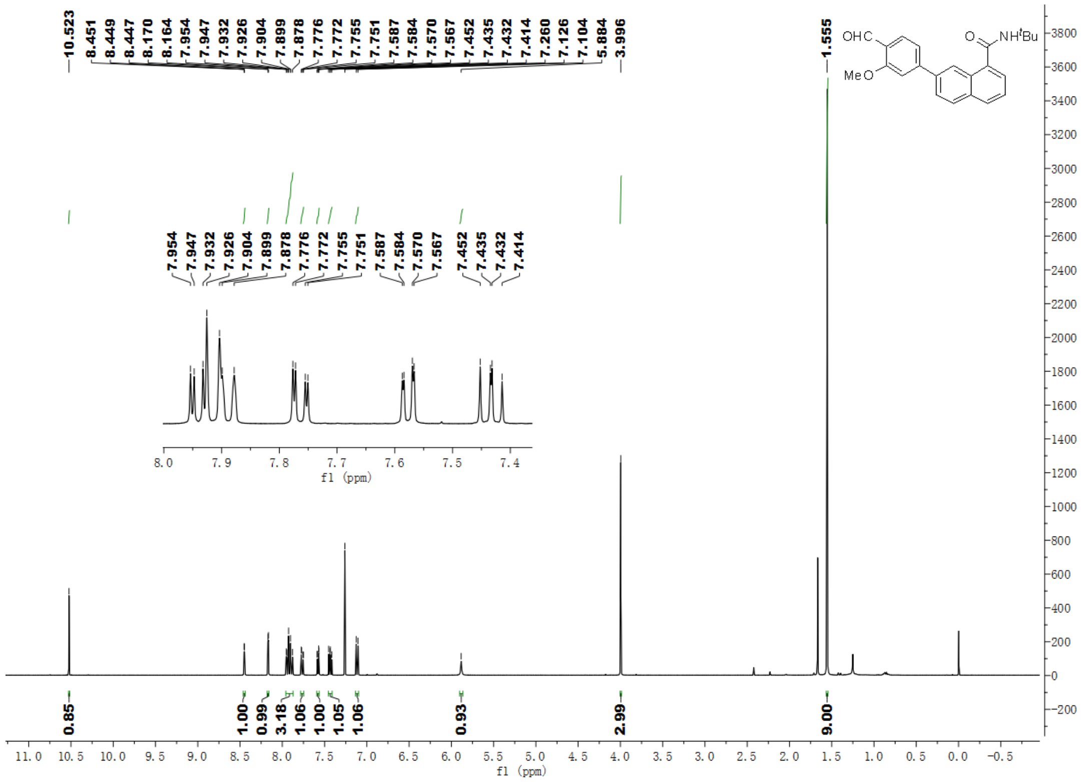

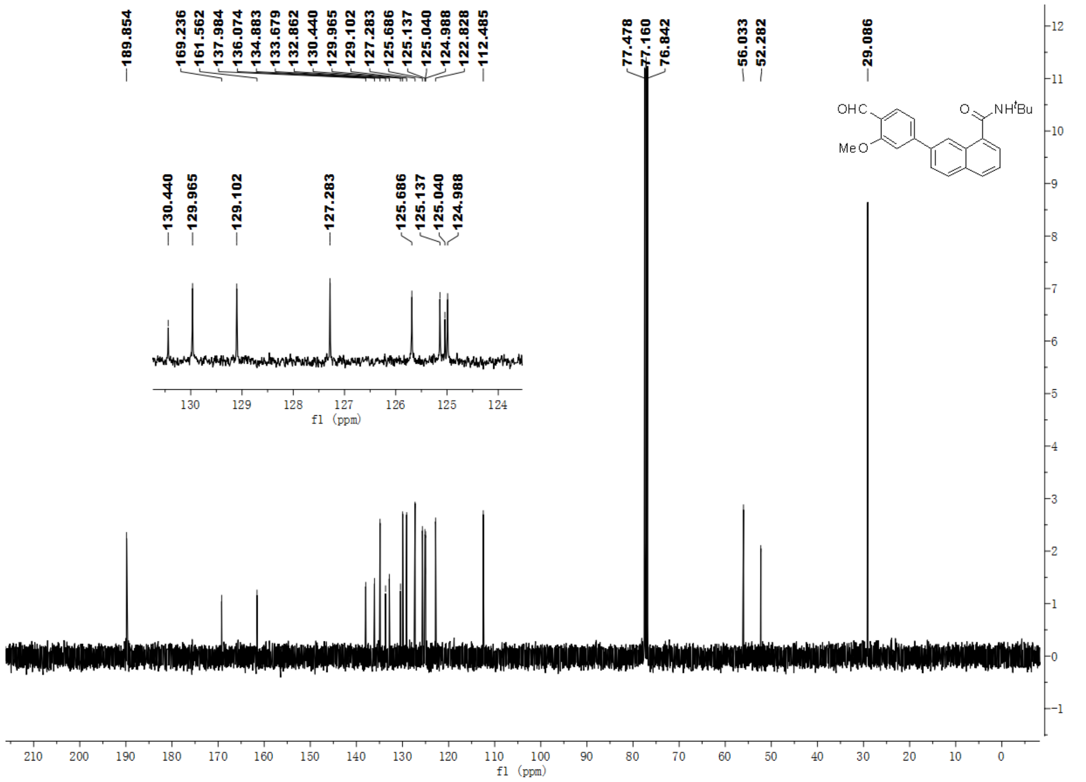

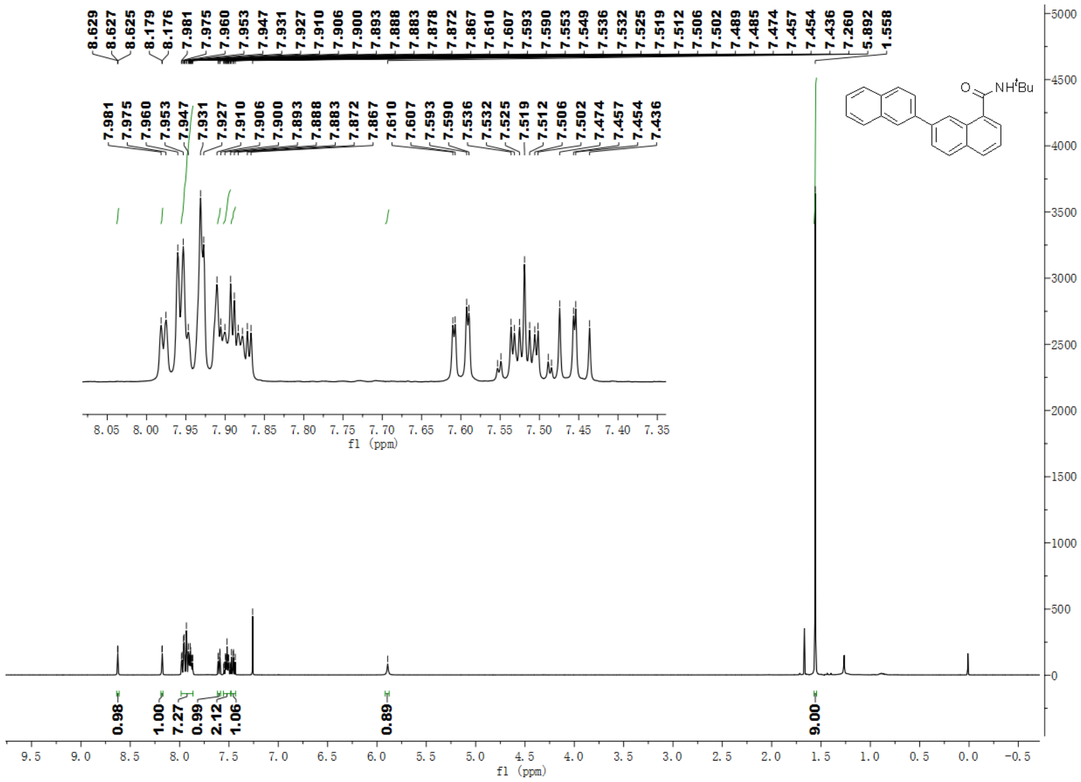

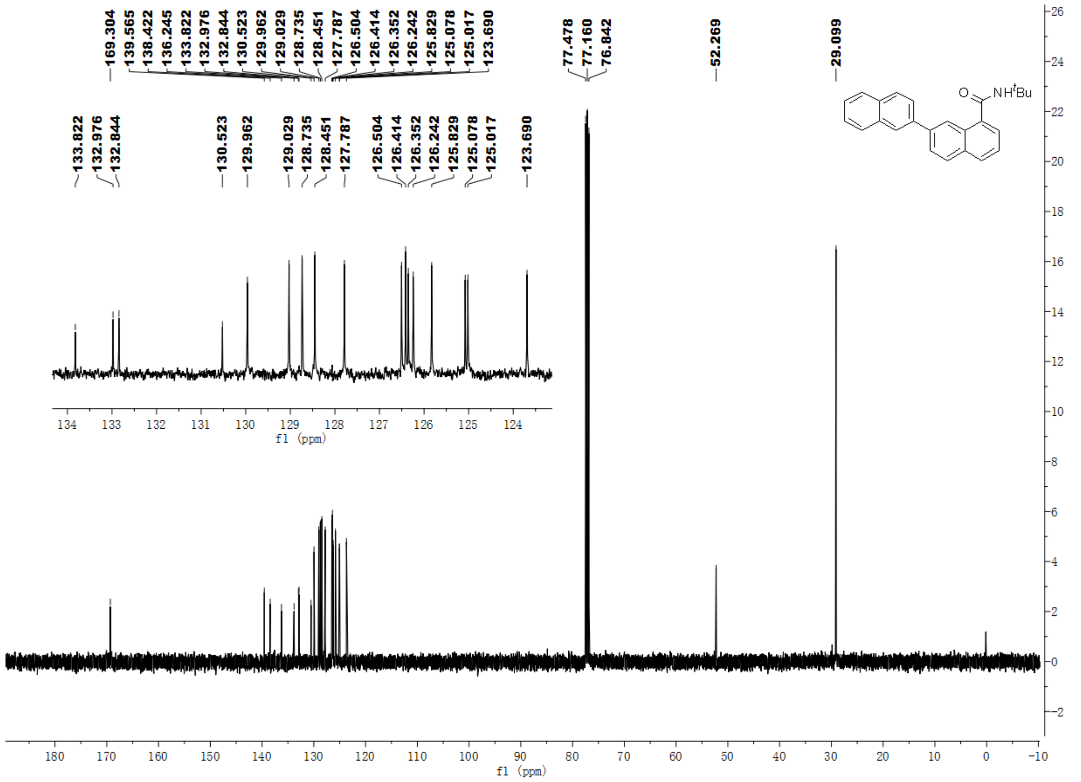

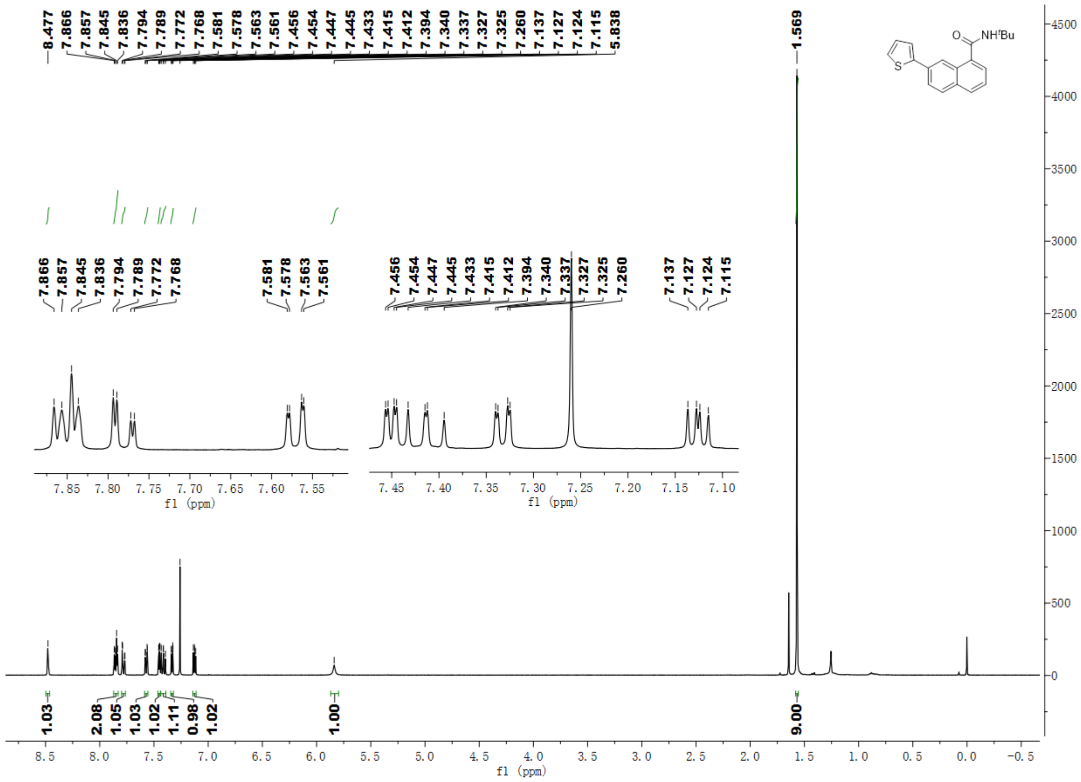
8.477
7.866
7.857
7.845
7.836
7.794
7.789
7.772
7.768
7.581
7.578
7.563
7.561
7.456
7.454
7.447
7.445
7.433
7.415
7.412
7.394
7.340
7.337
7.327
7.325
7.260
7.137
7.127
7.124
7.115
5.838
1.569
O
NH^{t}Bu
S
1.03
2.08
1.05
1.03
1.02
1.11
0.98
1.02
1.00
9.00
f1 (ppm)

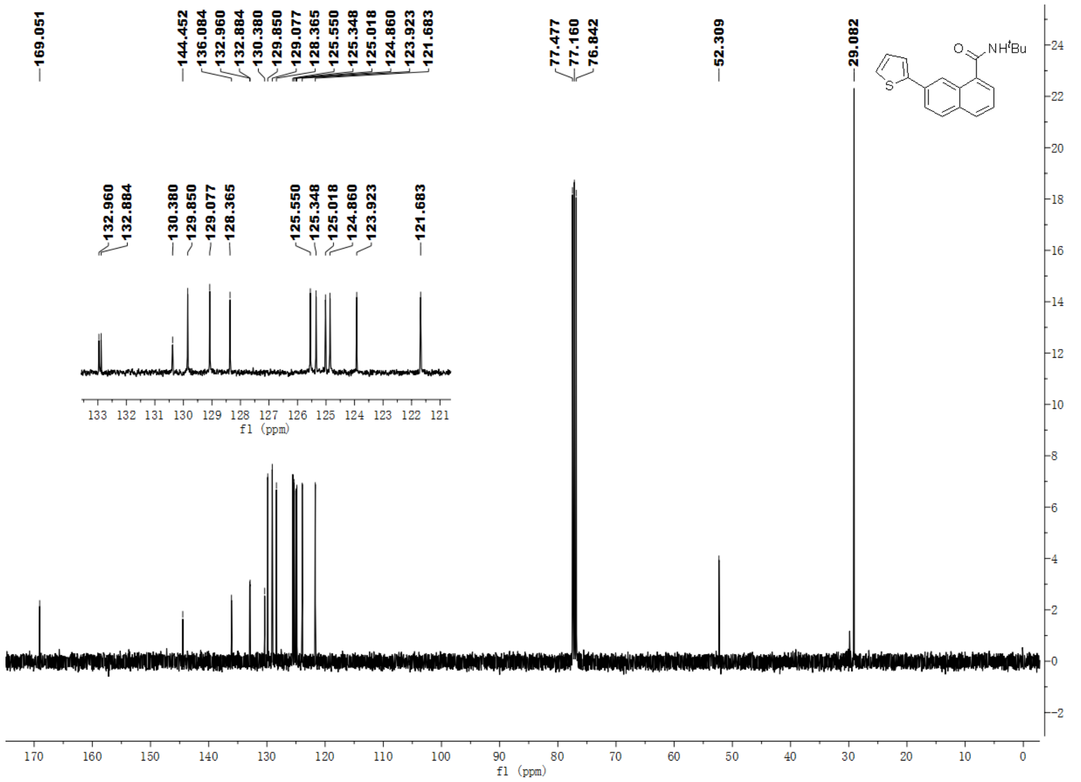
169.051
144.452
136.084
132.960
132.884
130.380
129.850
129.077
128.365
125.550
125.348
125.018
124.860
123.923
121.683
77.477
77.160
76.842
52.309
29.082
O
NH^{t}Bu
S
f1 (ppm)

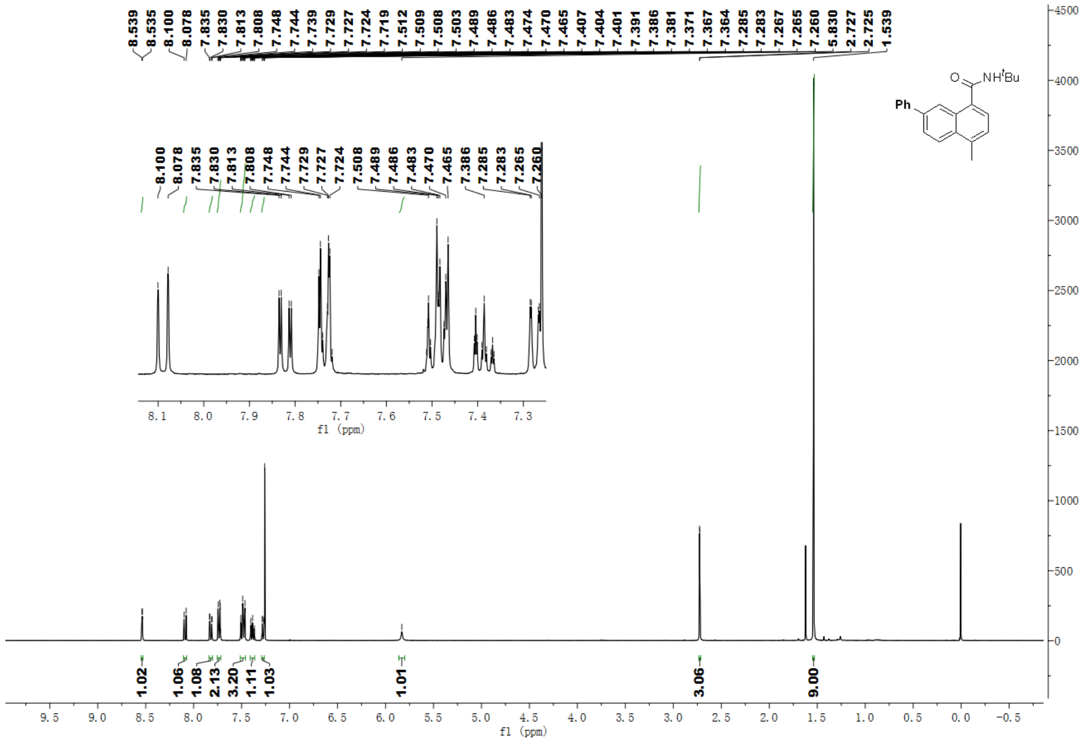

8.539
8.535
8.100
8.078
7.835
7.830
7.813
7.808
7.748
7.744
7.739
7.729
7.727
7.724
7.719
7.512
7.509
7.508
7.503
7.489
7.486
7.483
7.474
7.470
7.465
7.407
7.404
7.401
7.391
7.386
7.381
7.371
7.367
7.364
7.285
7.283
7.267
7.265
7.260
5.830
2.727
2.725
1.539
1.02
1.06
1.08
2.13
3.20
1.11
1.03
1.01
3.06
9.00
f1 (ppm)

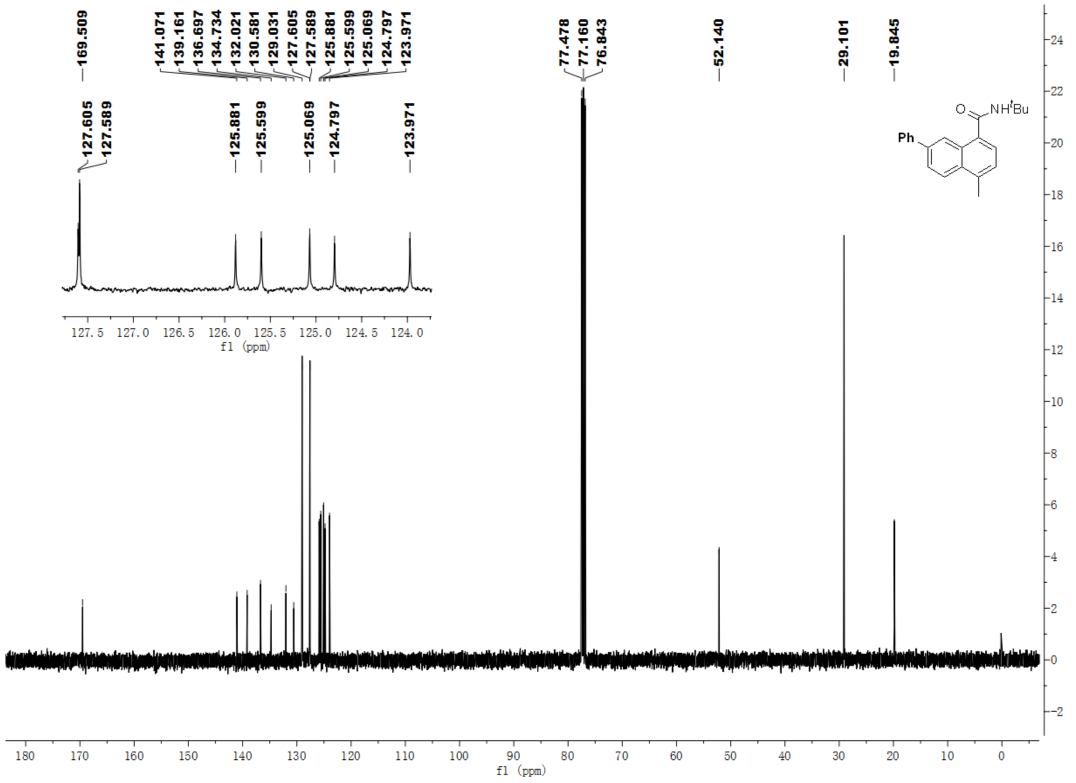

169.509
141.071
139.161
136.697
134.734
132.021
130.581
129.031
127.605
127.589
125.881
125.599
125.069
124.797
123.971
77.478
77.160
76.843
52.140
29.101
19.845
f1 (ppm)

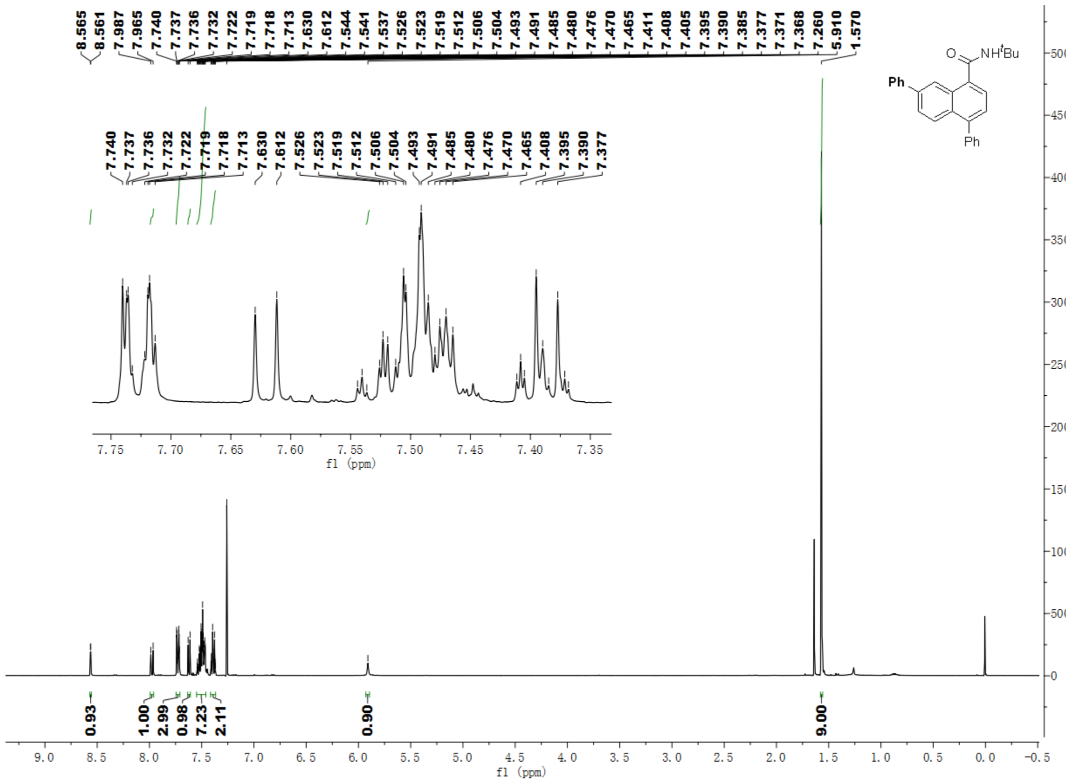

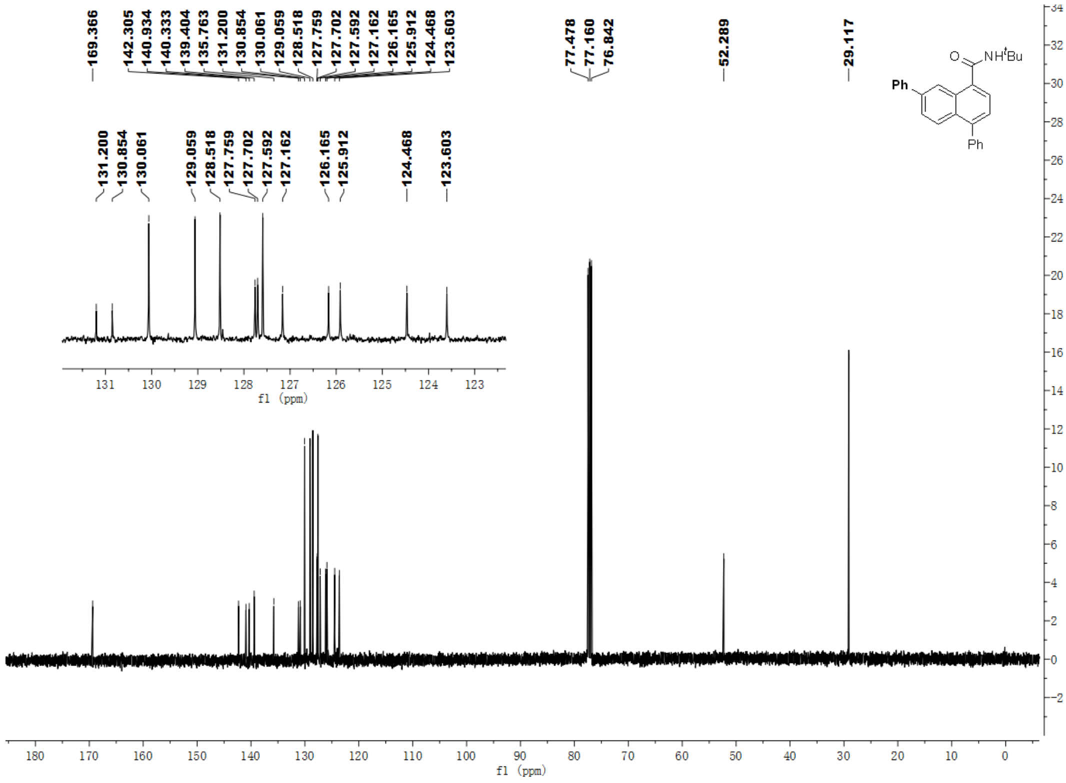

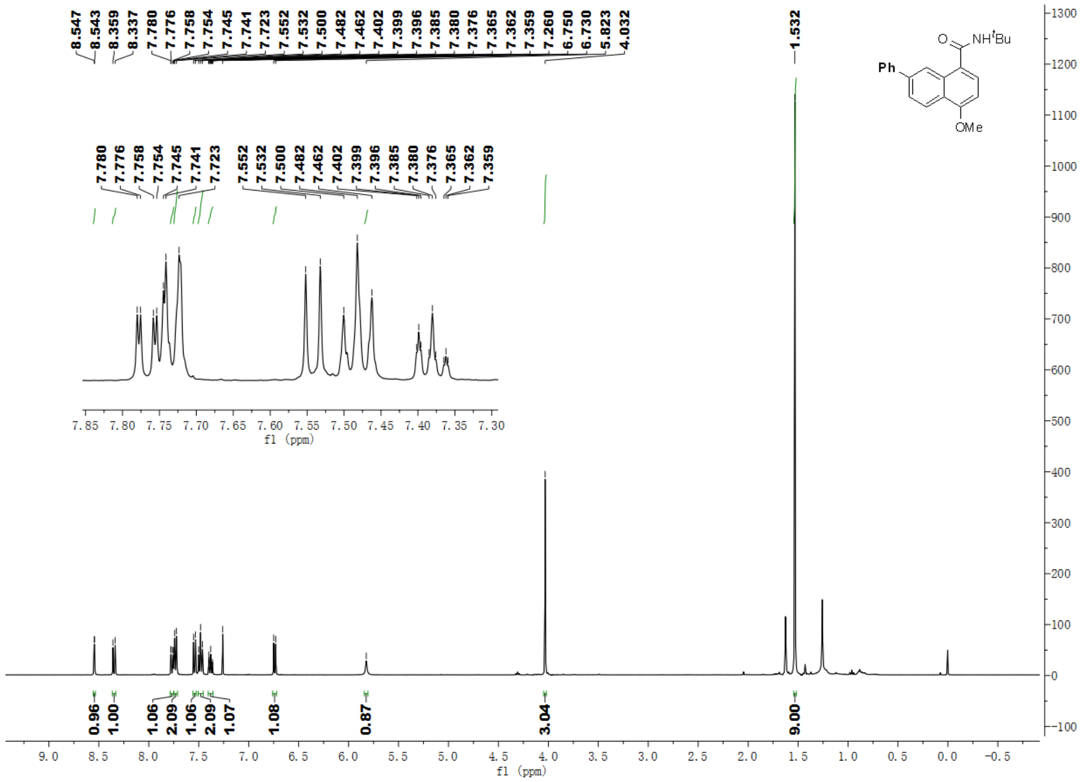
8.547
8.543
8.359
8.337
7.780
7.776
7.758
7.754
7.745
7.741
7.723
7.552
7.532
7.500
7.482
7.462
7.402
7.399
7.396
7.385
7.380
7.376
7.365
7.362
7.359
7.260
6.750
6.730
5.823
4.032
1.532
O
NH^{t}Bu
Ph
OMe
0.96
1.00
1.06
2.09
1.06
2.09
1.07
1.08
0.87
3.04
9.00
f1 (ppm)

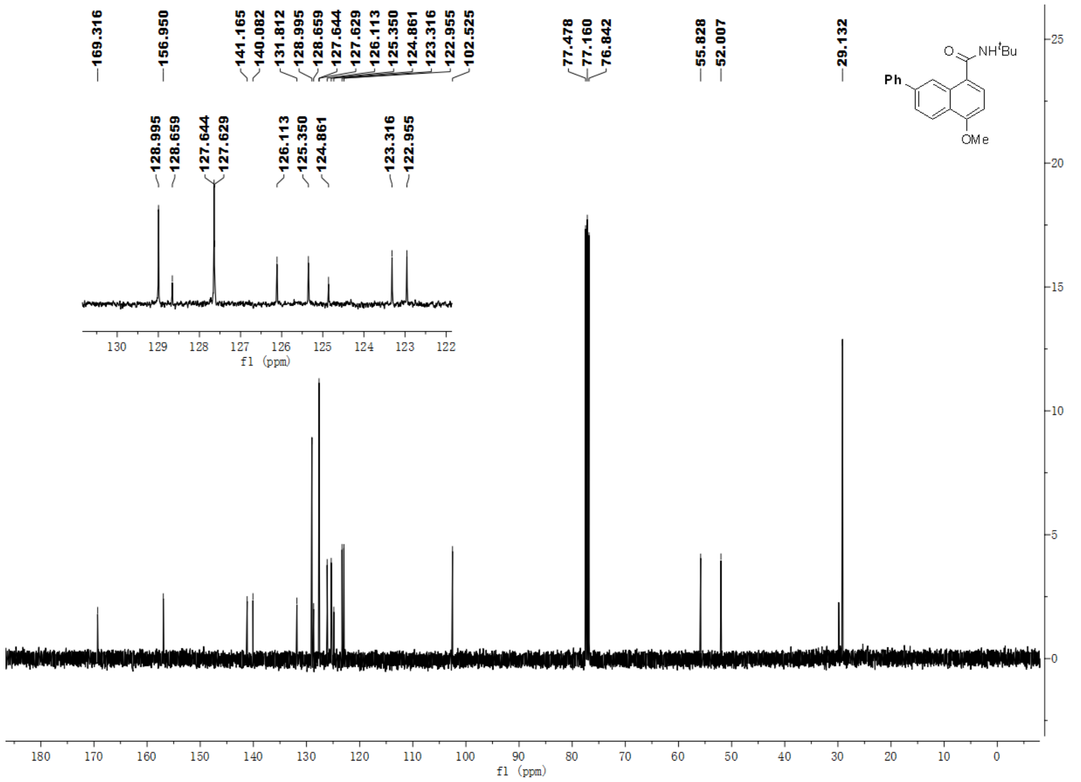
169.316
156.950
141.165
140.082
131.812
128.995
128.659
127.644
127.629
126.113
125.350
124.861
123.316
122.955
102.525
77.478
77.160
76.842
55.828
52.007
29.132
O
NH^{t}Bu
Ph
OMe
f1 (ppm)

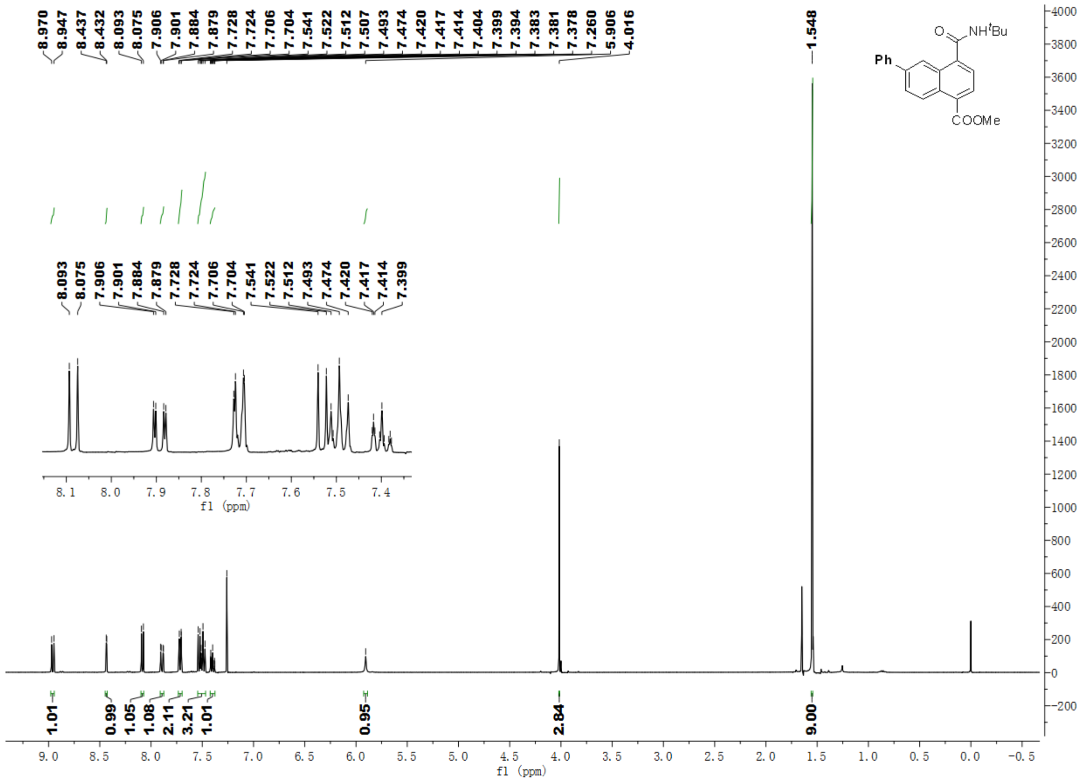
8.970
8.947
8.437
8.432
8.093
8.075
7.906
7.901
7.884
7.879
7.728
7.724
7.706
7.704
7.541
7.522
7.512
7.507
7.493
7.474
7.420
7.417
7.414
7.404
7.399
7.394
7.383
7.381
7.378
7.260
5.906
4.016
1.548
Ph
O
NH^{t}Bu
COOMe
1.01
0.99
1.05
1.08
2.11
3.21
1.01
0.95
2.84
9.00
f1 (ppm)

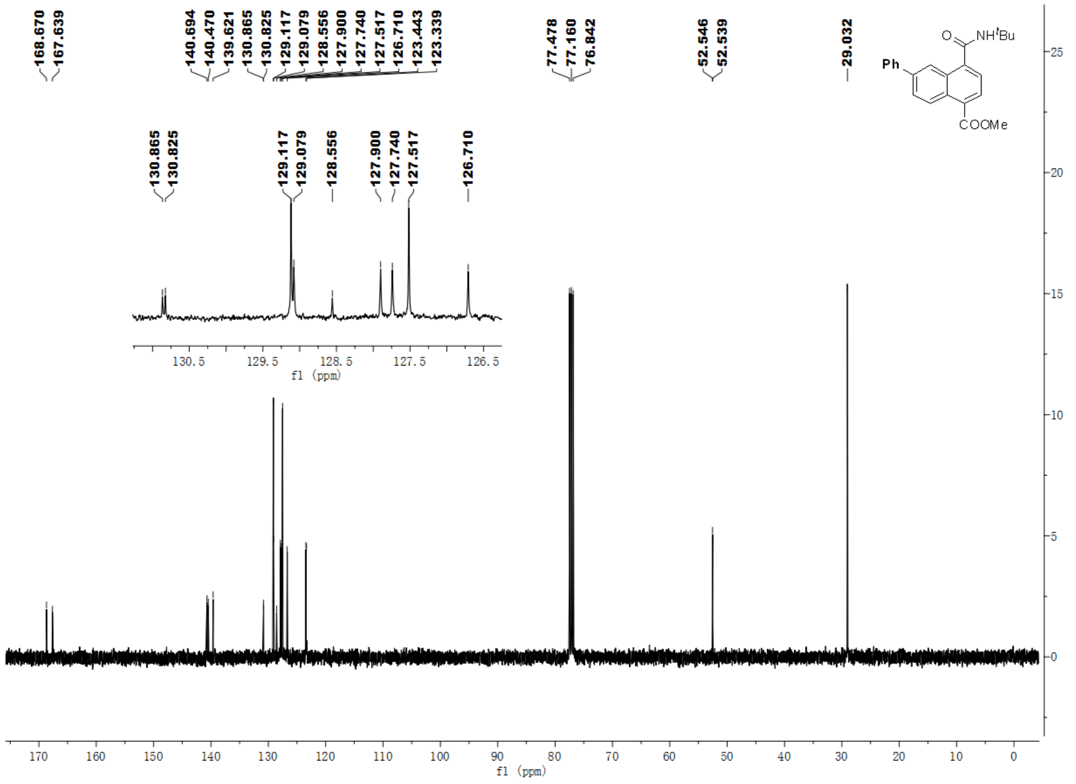
168.670
167.639
140.694
140.470
139.621
130.865
130.825
129.117
129.079
128.556
127.900
127.740
127.517
126.710
123.443
123.339
77.478
77.160
76.842
52.546
52.539
29.032
Ph
O
NH^{t}Bu
COOMe
f1 (ppm)

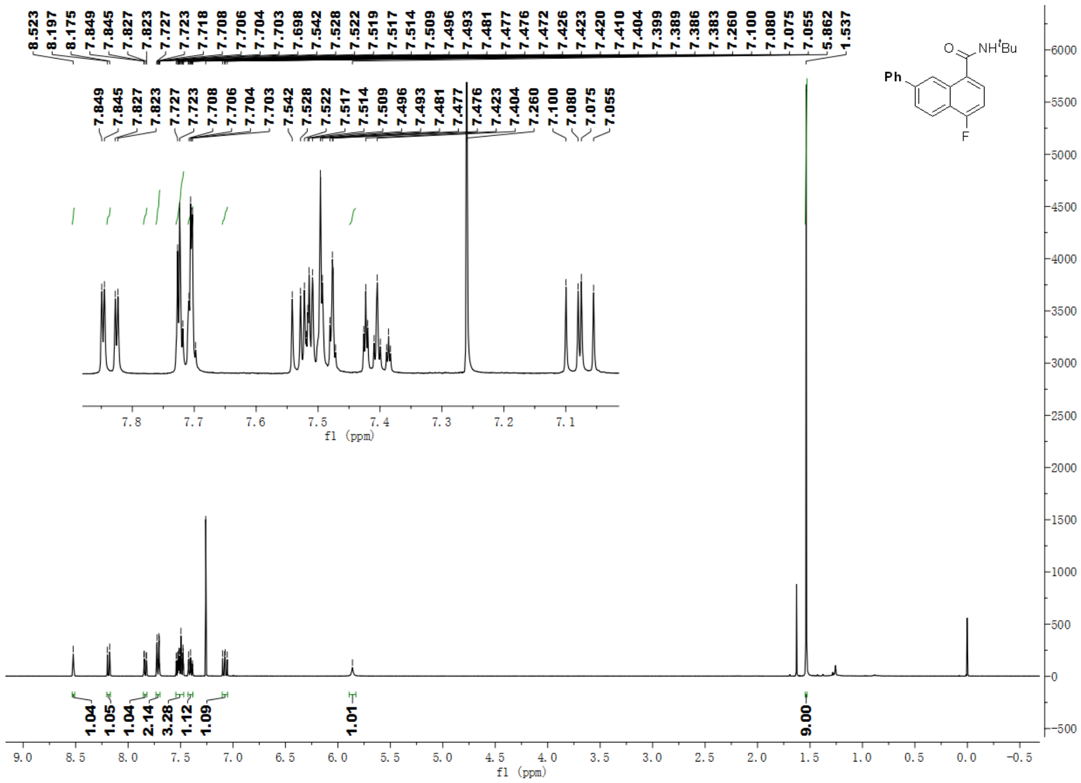
8.523
8.197
8.175
7.849
7.845
7.827
7.823
7.727
7.723
7.718
7.708
7.706
7.704
7.703
7.698
7.542
7.528
7.522
7.519
7.517
7.514
7.509
7.496
7.493
7.481
7.477
7.476
7.472
7.426
7.423
7.420
7.410
7.404
7.399
7.389
7.386
7.383
7.260
7.100
7.080
7.075
7.055
5.862
1.537
O
NH^{t}Bu
Ph
F
1.04
1.05
1.04
2.14
3.28
1.12
1.09
1.01
9.00
f1 (ppm)

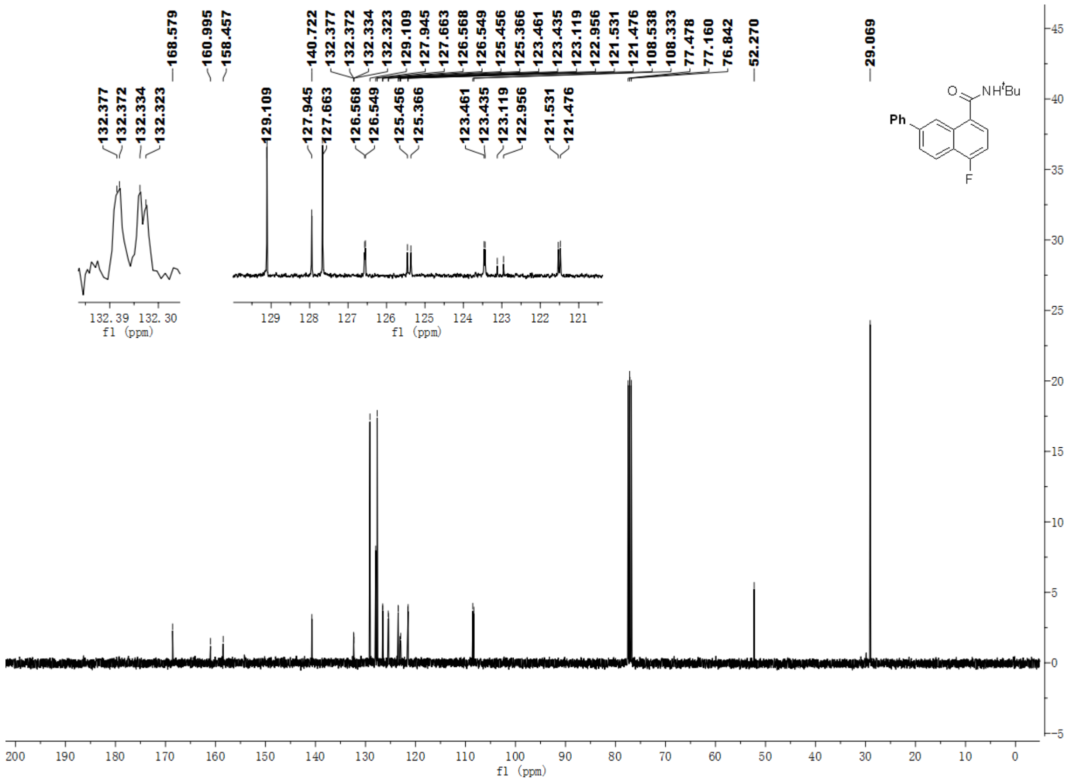
168.579
160.995
158.457
140.722
132.377
132.372
132.334
132.323
129.109
127.945
127.663
126.568
126.549
125.456
125.366
123.461
123.435
123.119
122.956
121.531
121.476
108.538
108.333
77.478
77.160
76.842
52.270
29.069
O
NH^{t}Bu
Ph
F
f1 (ppm)

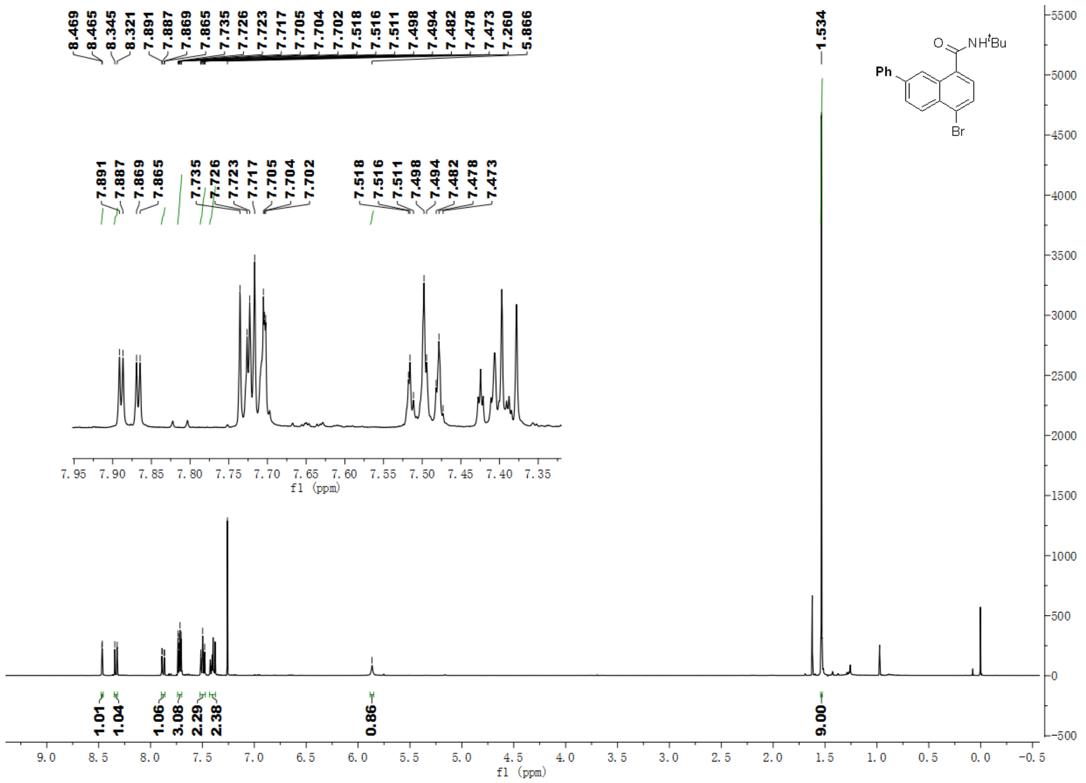

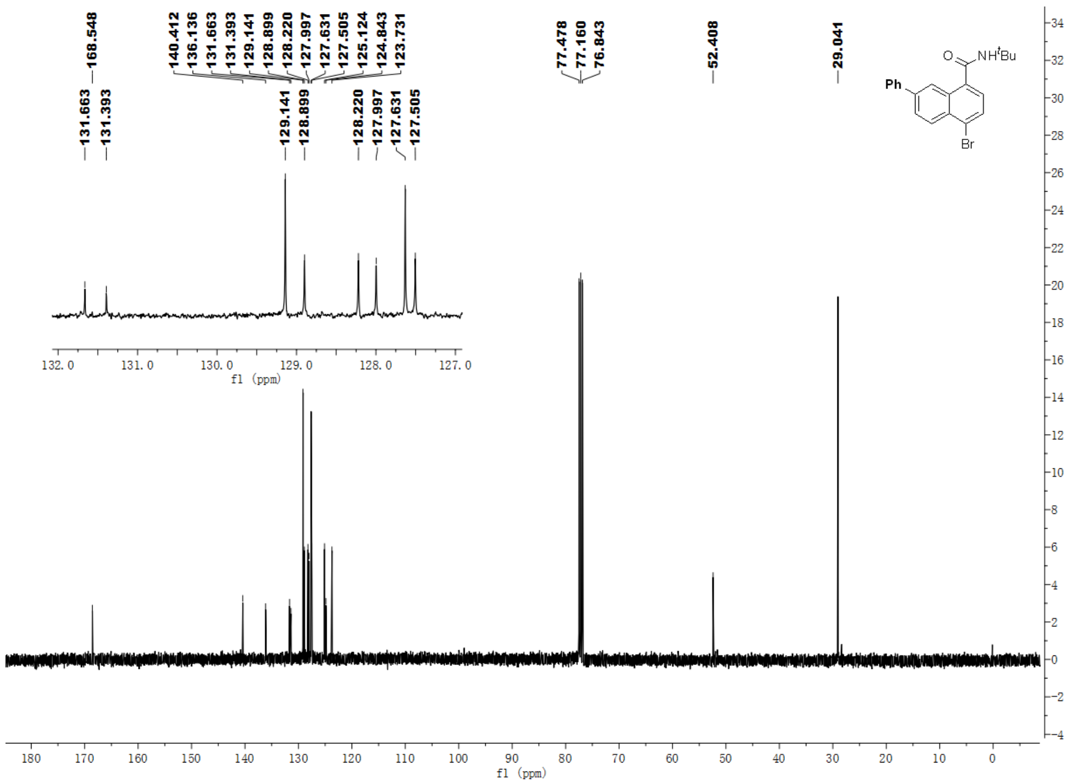

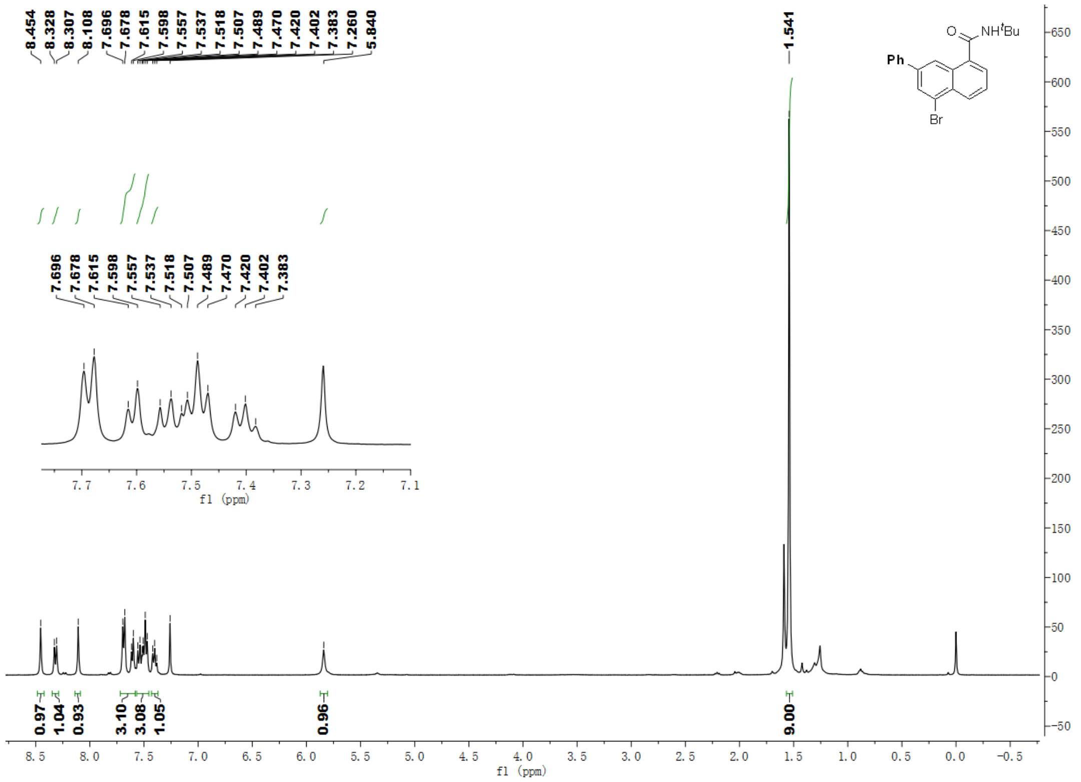

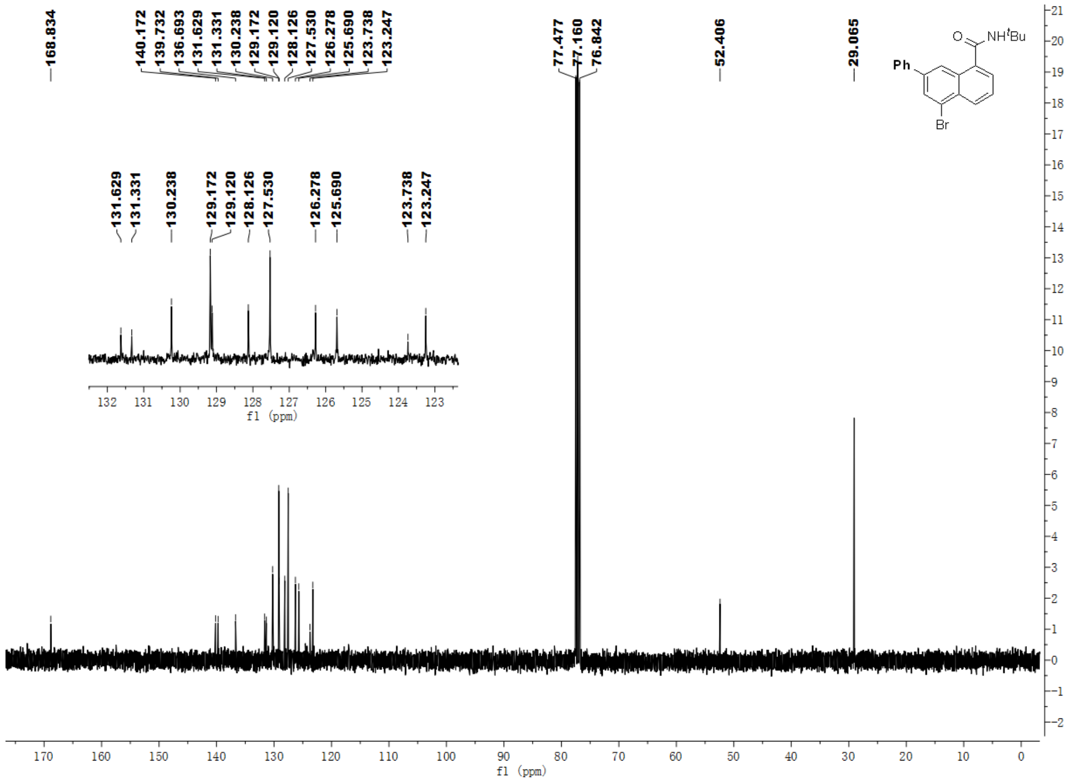

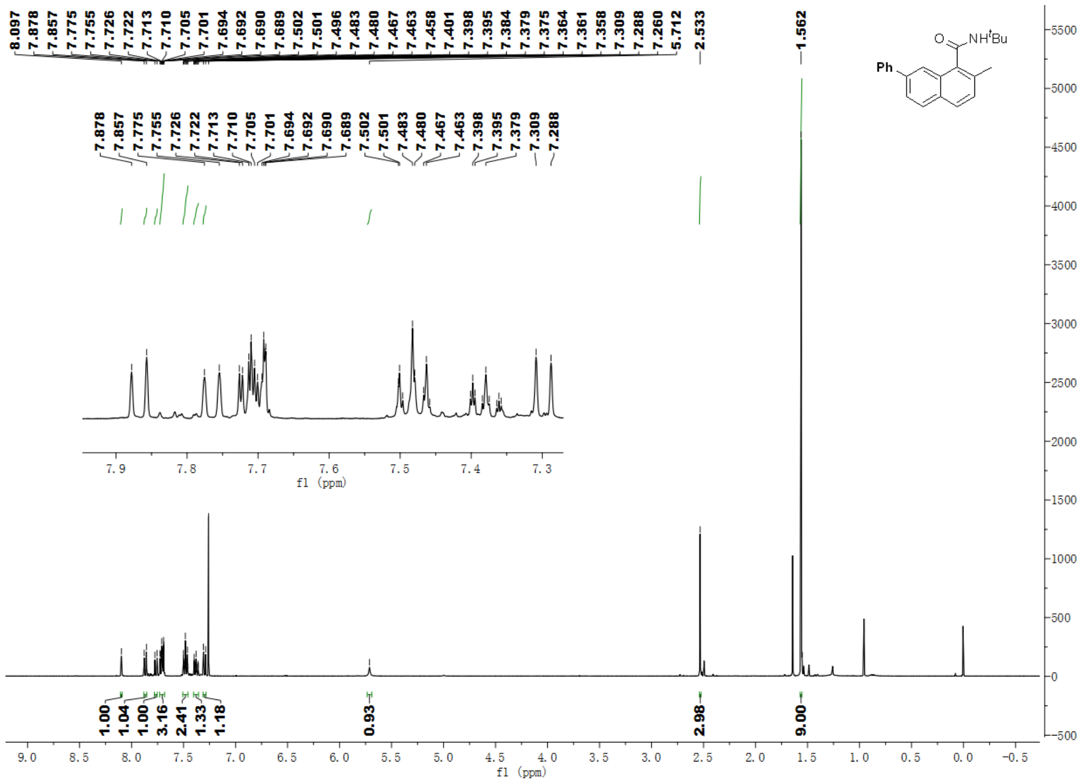

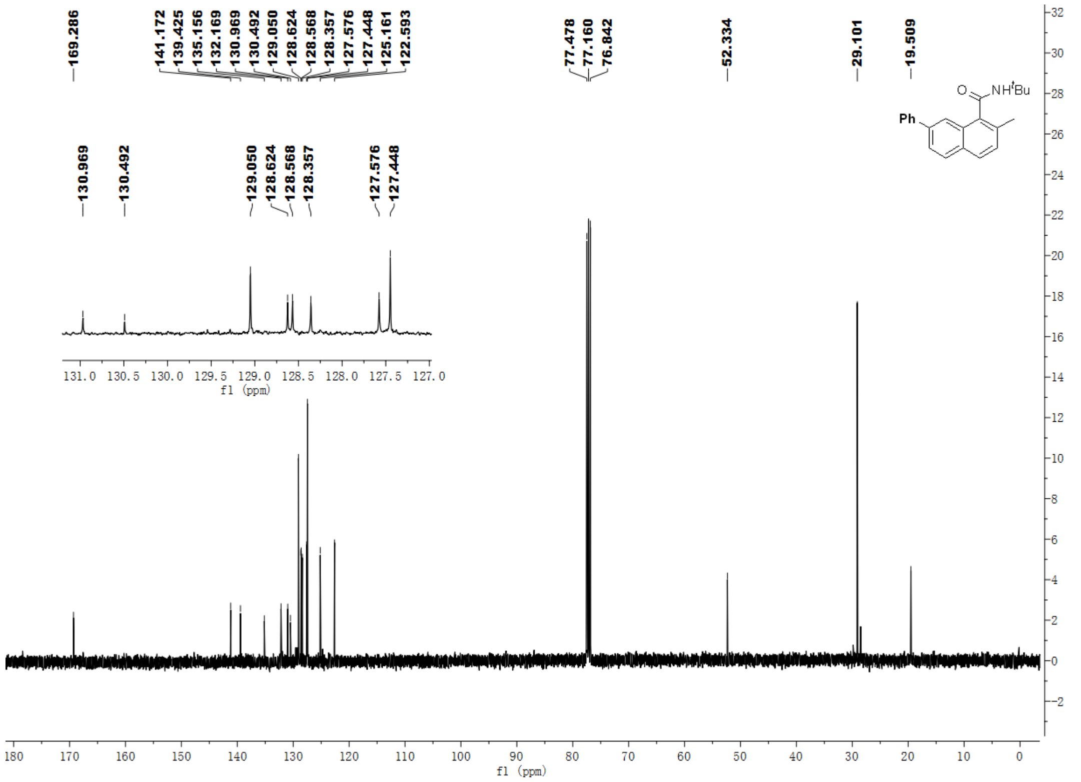

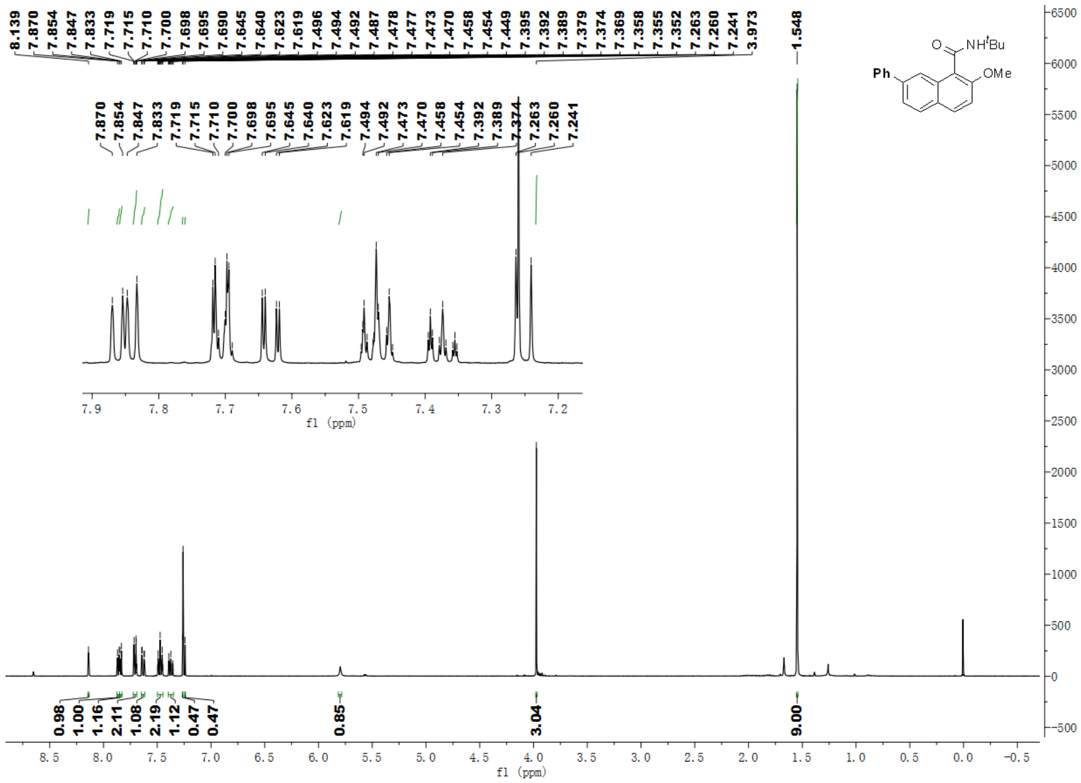
8.139
7.870
7.854
7.847
7.833
7.719
7.715
7.710
7.700
7.698
7.695
7.690
7.645
7.640
7.623
7.619
7.496
7.494
7.492
7.487
7.478
7.477
7.473
7.470
7.458
7.454
7.449
7.395
7.392
7.389
7.379
7.374
7.369
7.358
7.355
7.352
7.263
7.260
7.241
3.973
1.548
0.98
1.00
1.16
2.11
1.08
2.19
1.12
0.47
0.47
0.85
3.04
9.00
f1 (ppm)

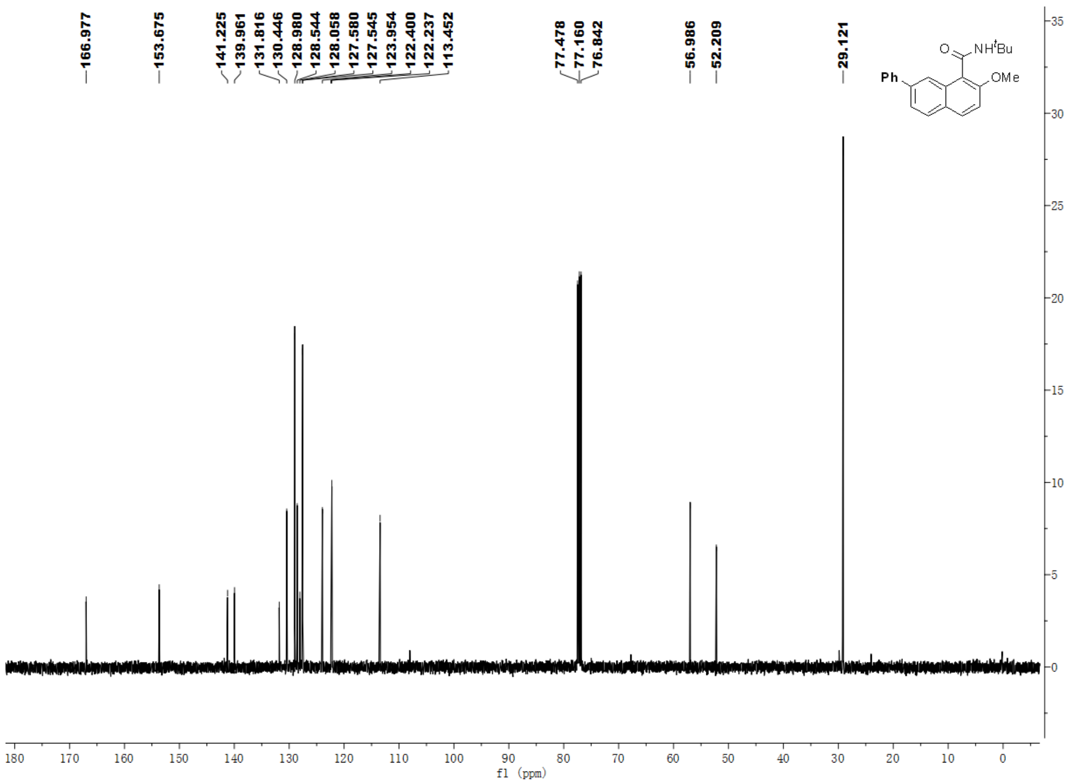
166.977
153.675
141.225
139.961
131.816
130.446
128.980
128.544
128.058
127.580
127.545
123.954
122.400
122.237
113.452
77.478
77.160
76.842
56.986
52.209
29.121
f1 (ppm)

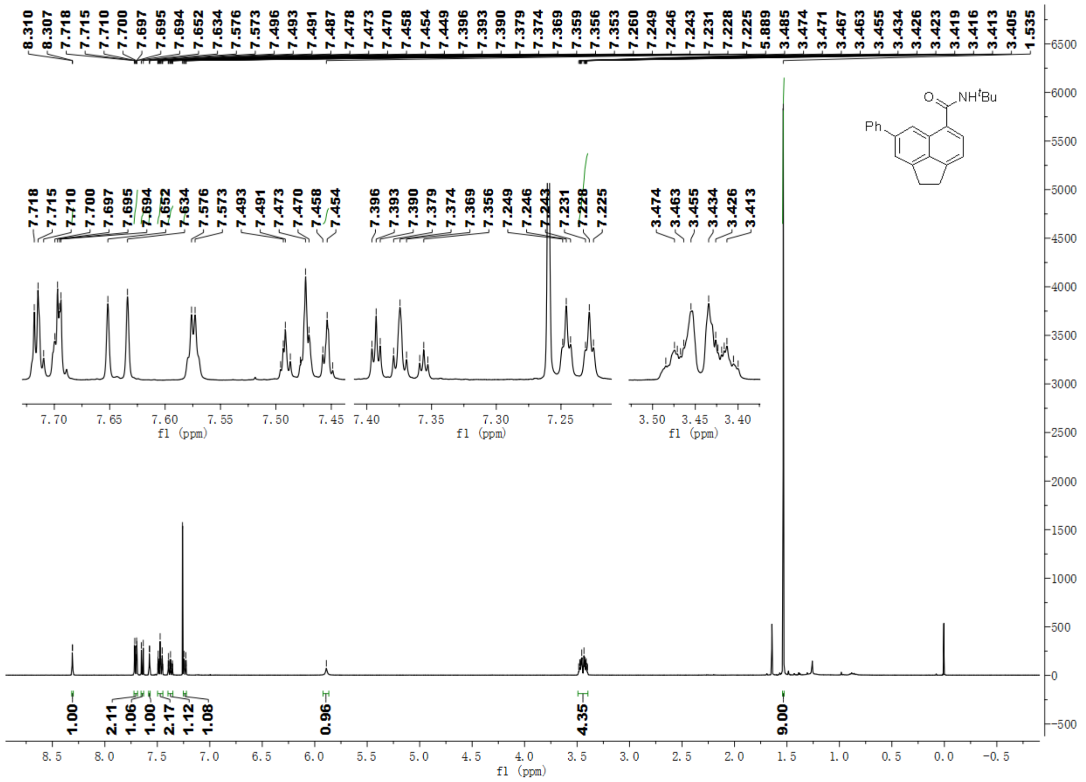

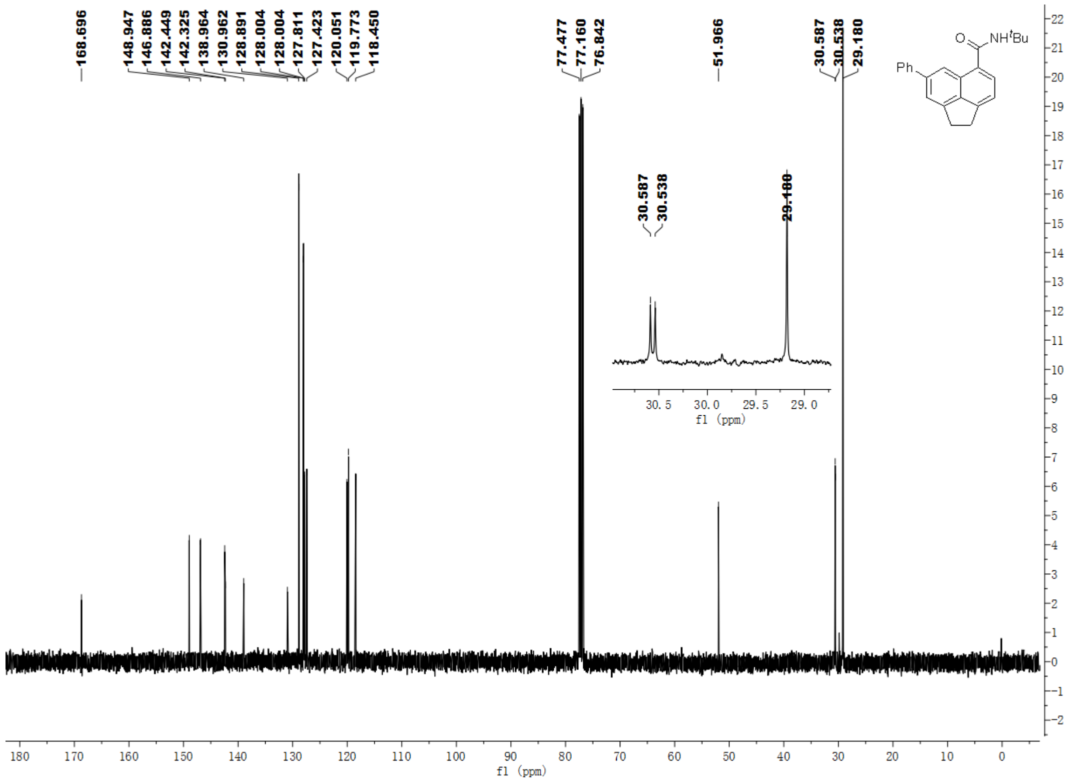

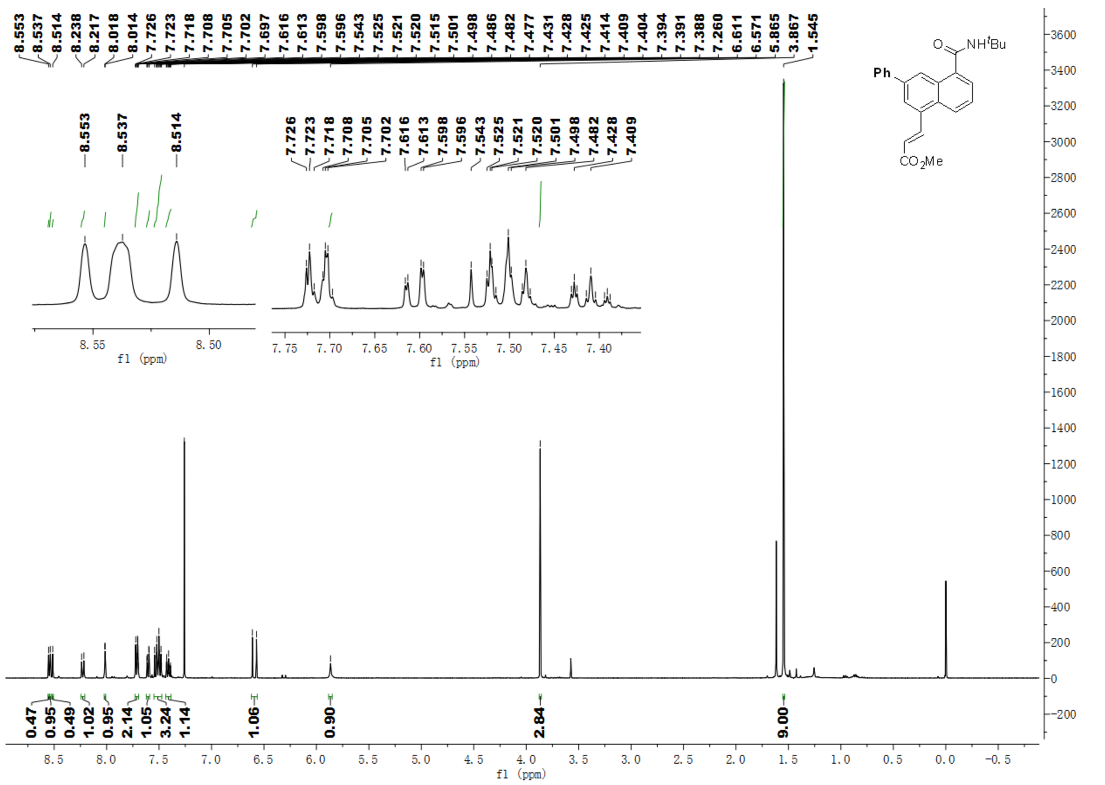

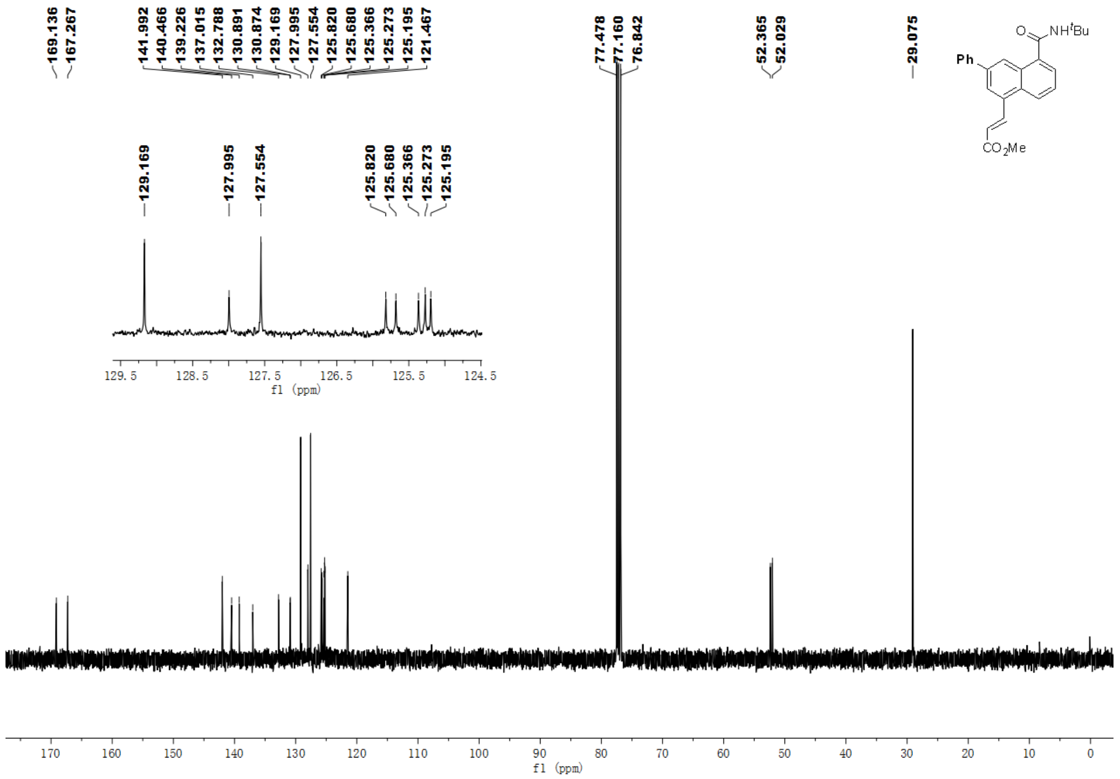

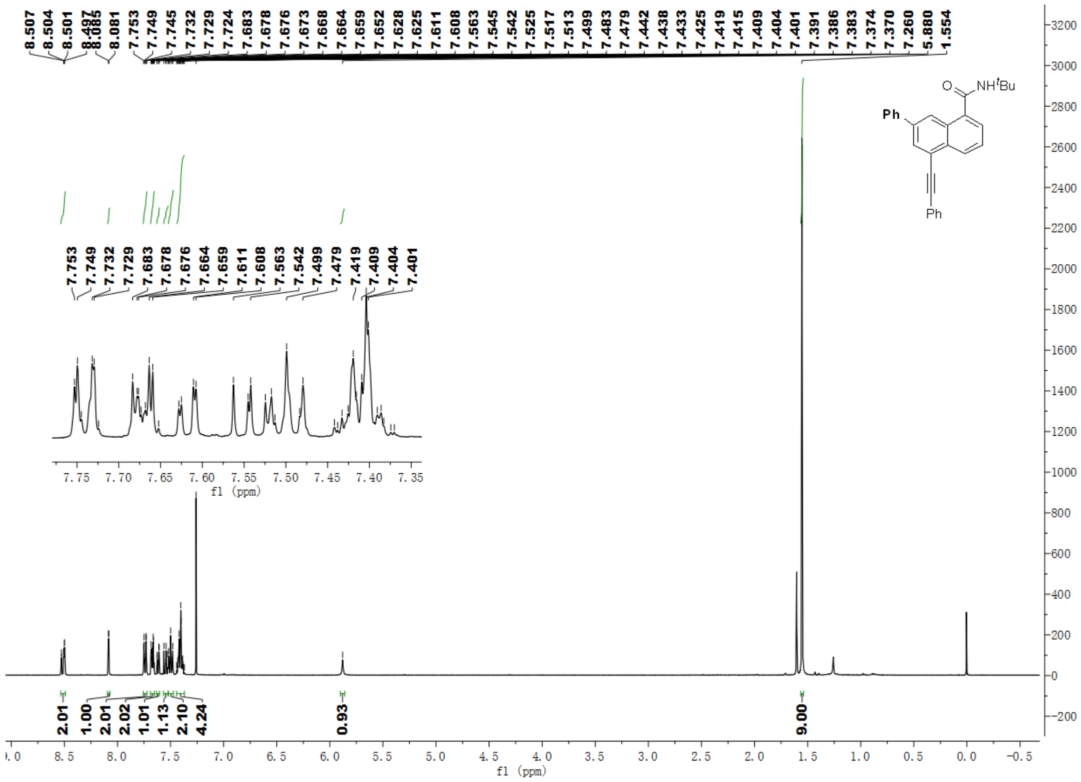
O
NH^tBu
Ph
Ph
f1 (ppm)
f1 (ppm)

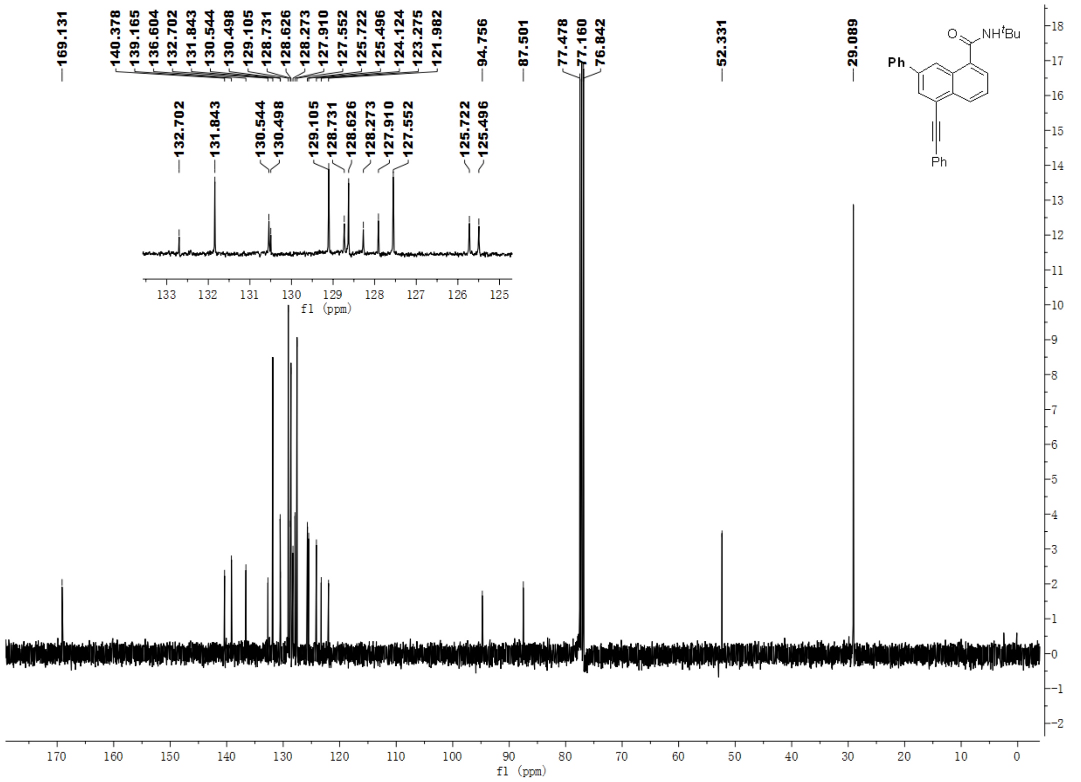
O
NH^tBu
Ph
Ph
f1 (ppm)
f1 (ppm)

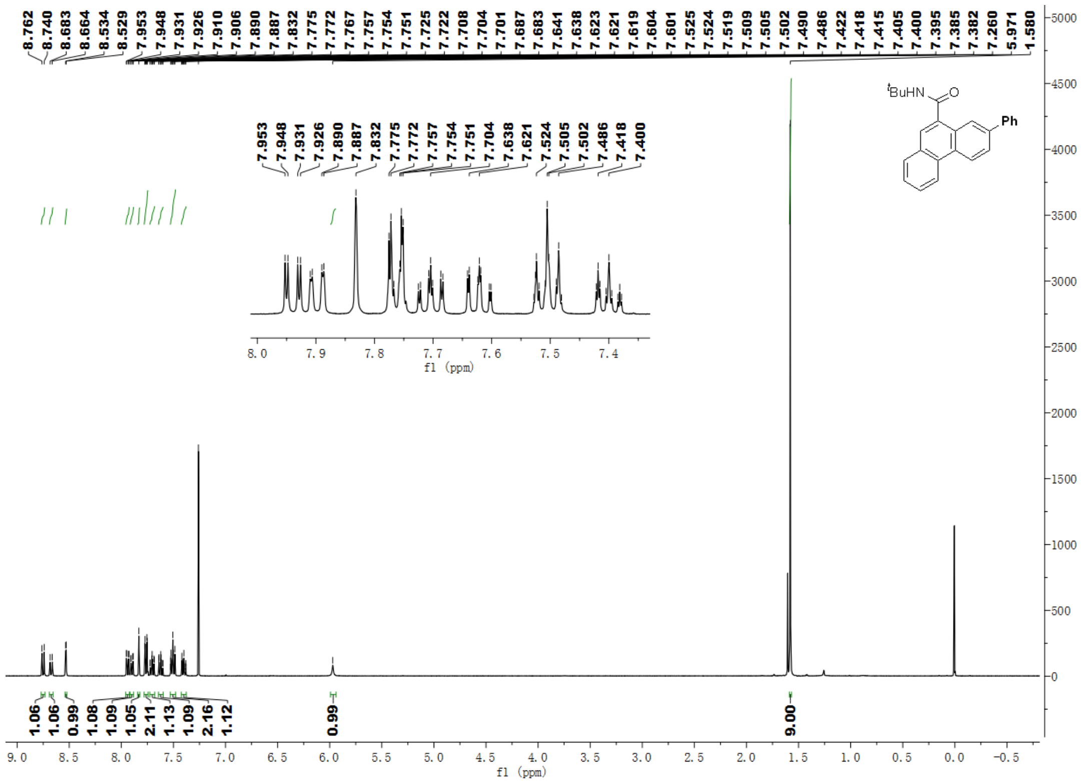

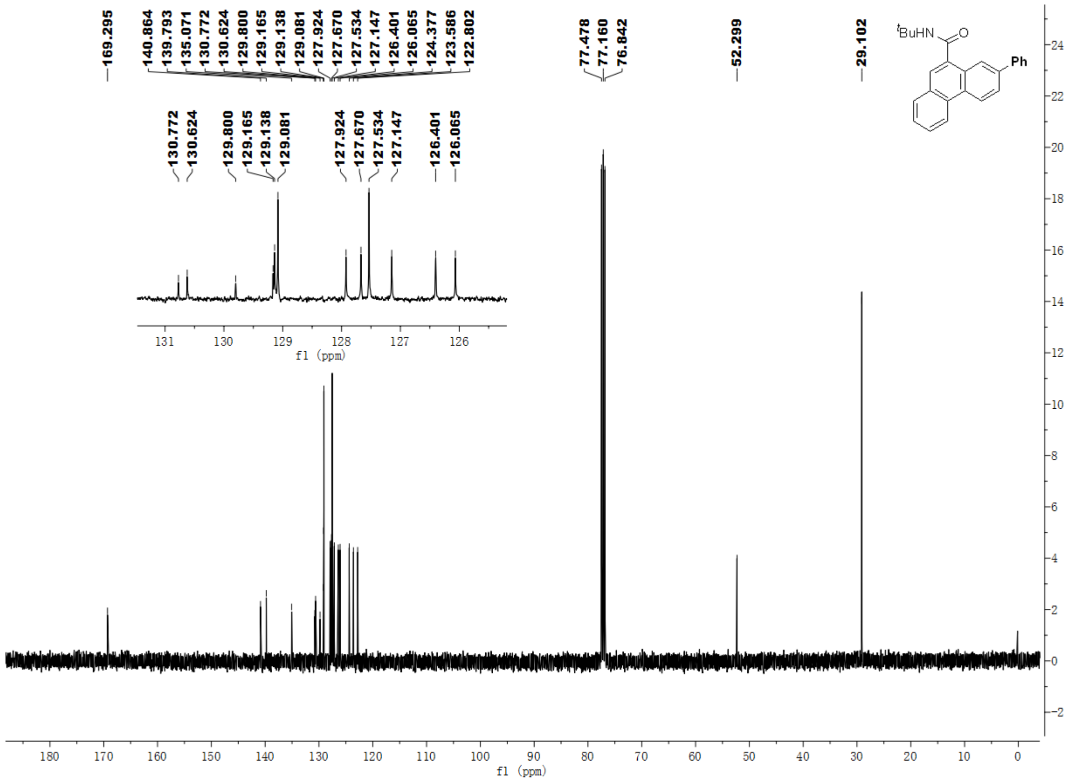

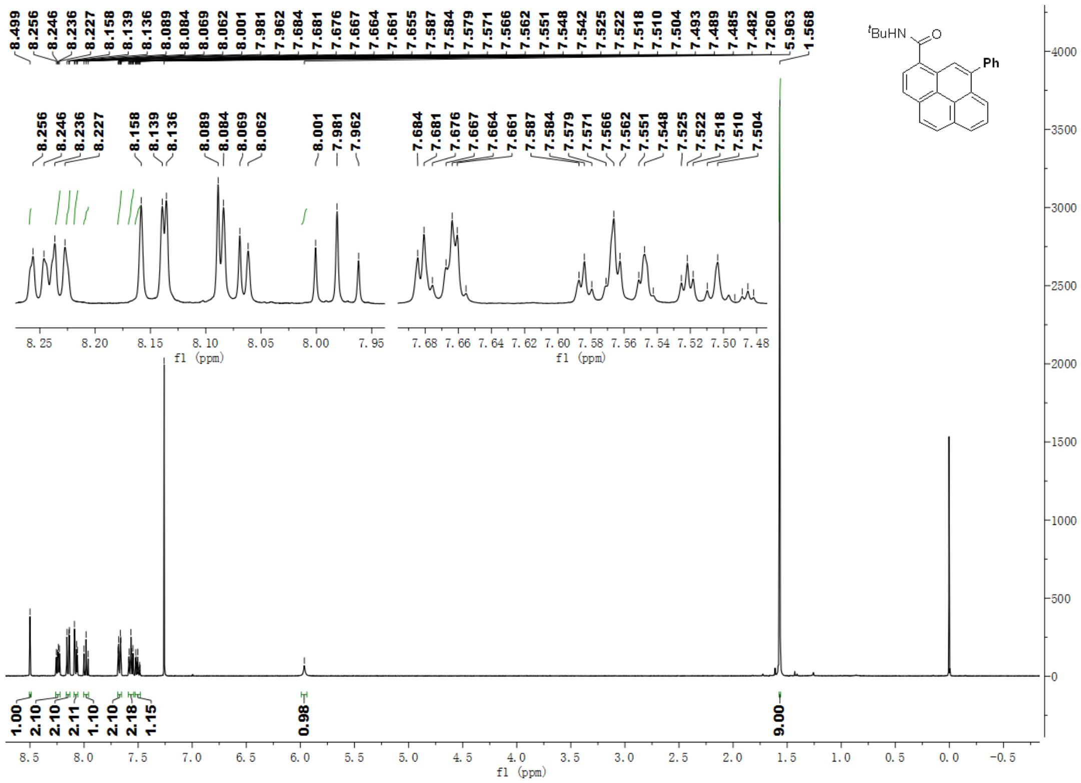

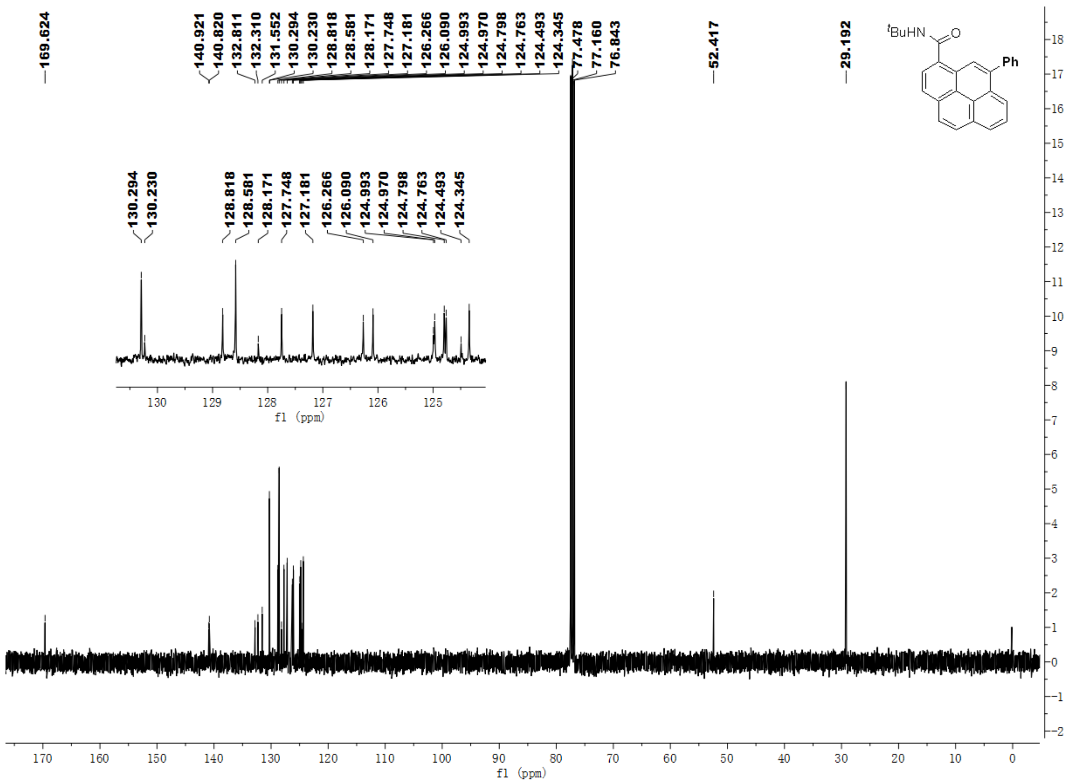

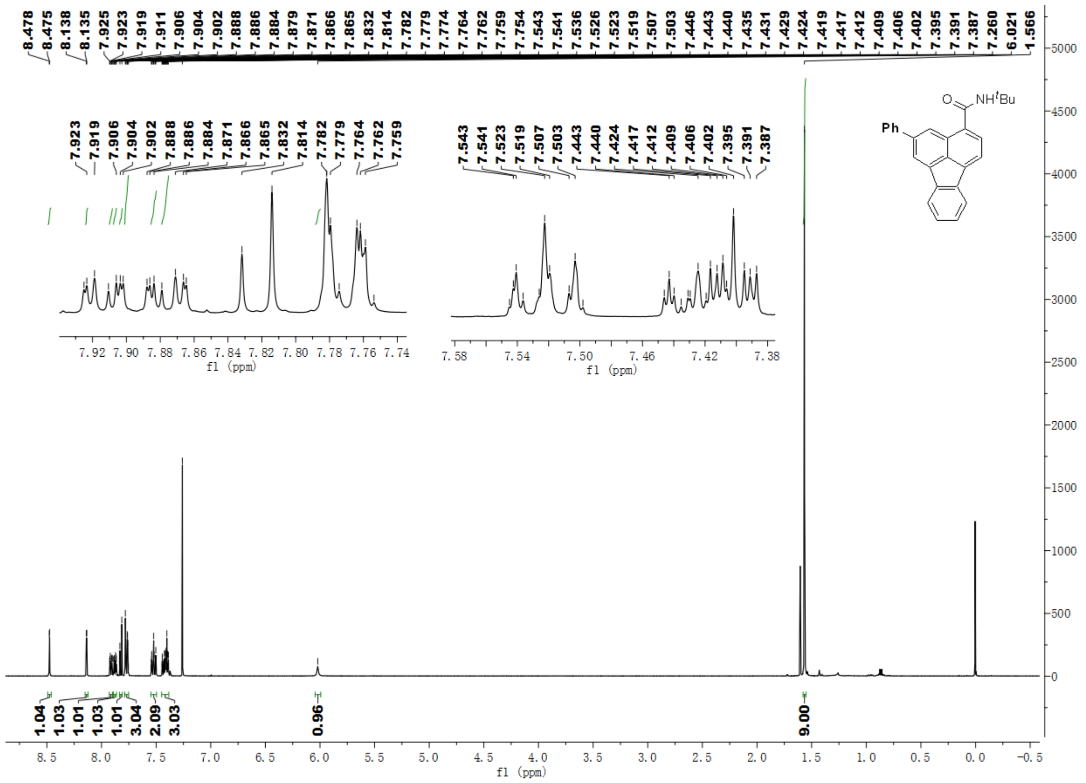
Ph
O
NH^{t}Bu
f1 (ppm)

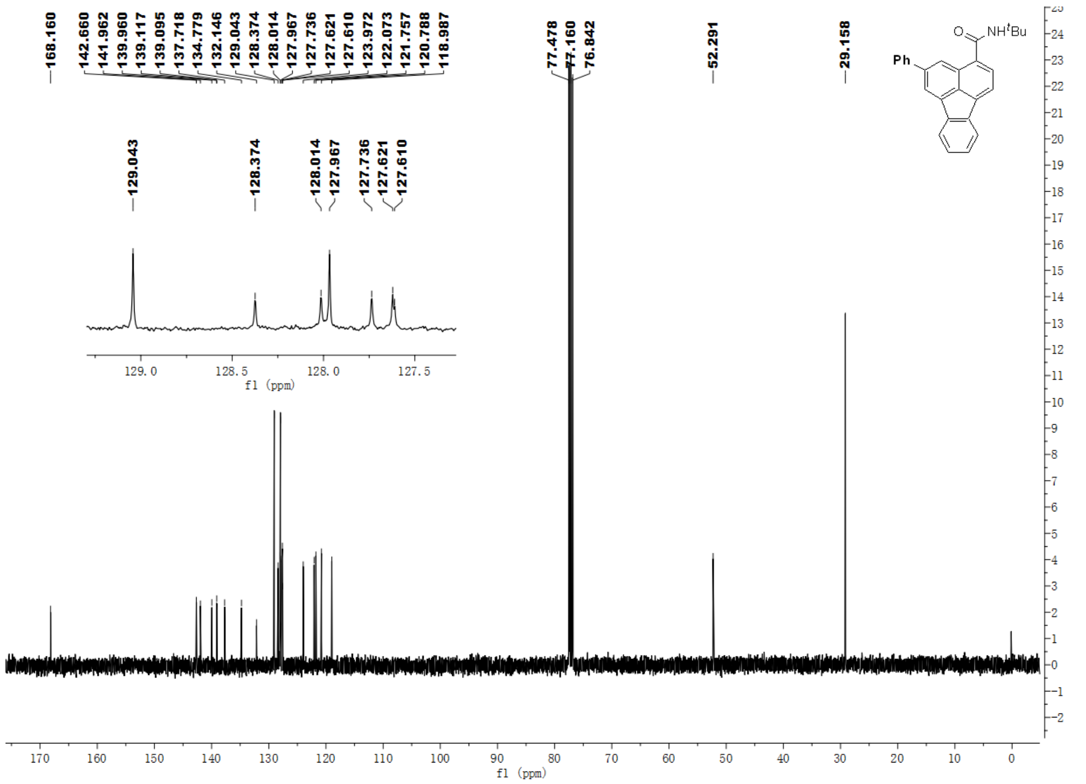
Ph
O
NH^{t}Bu
f1 (ppm)

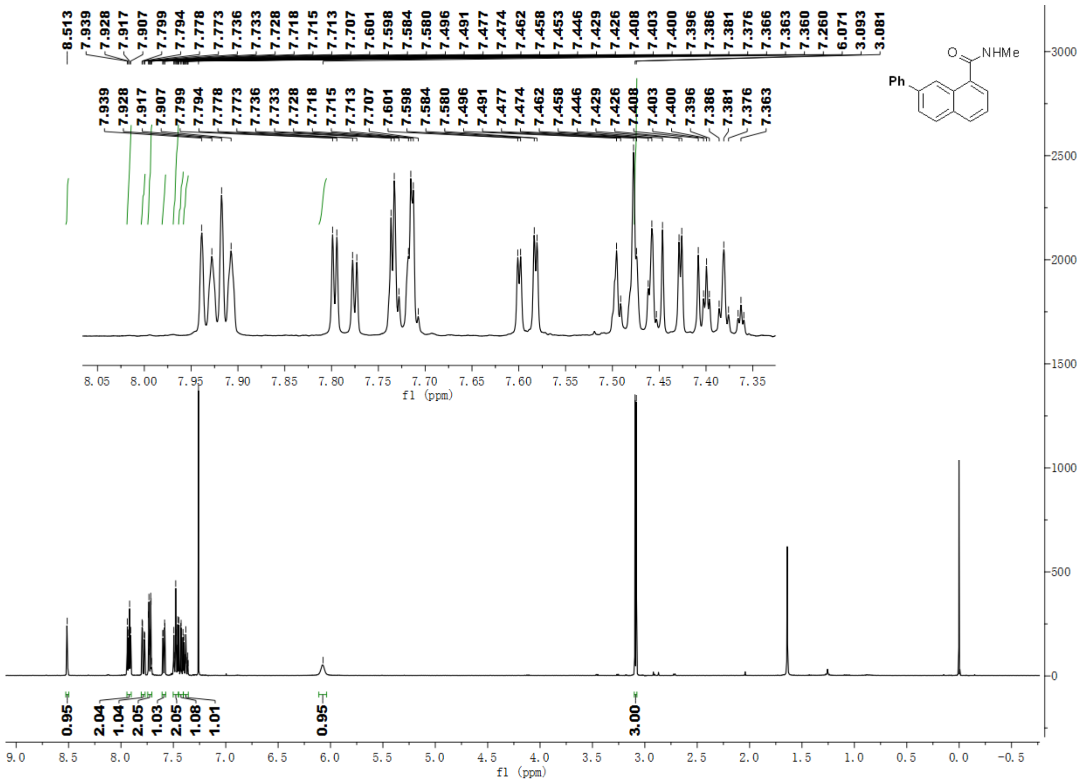

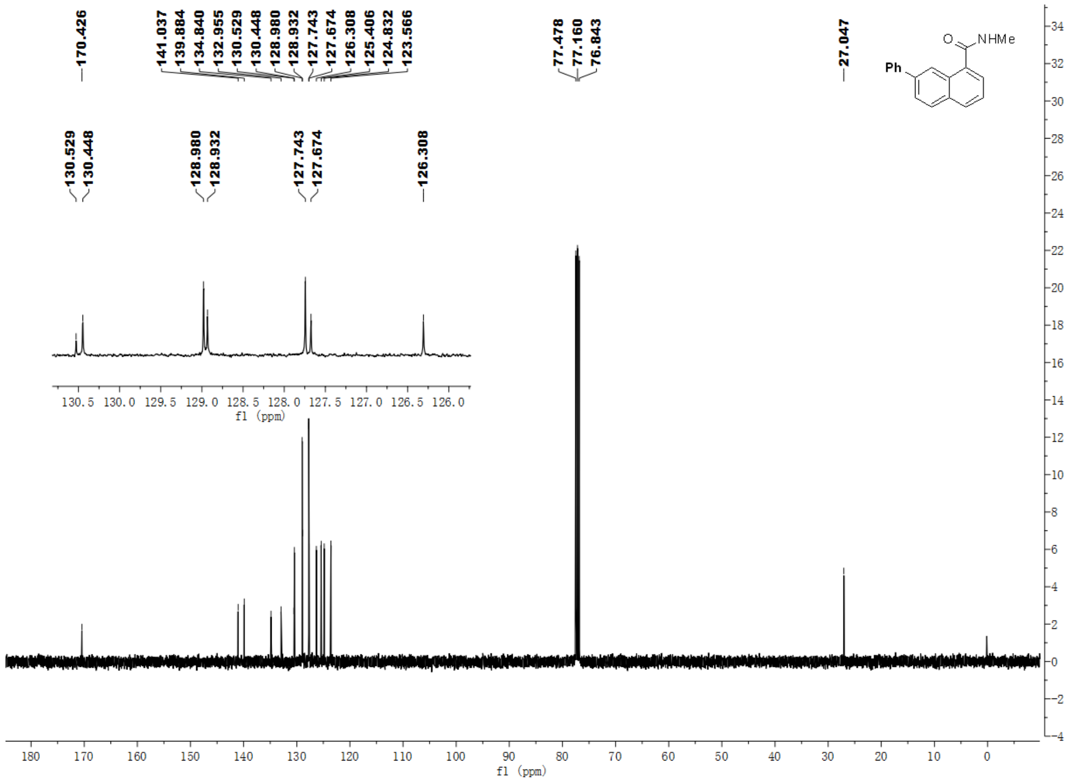

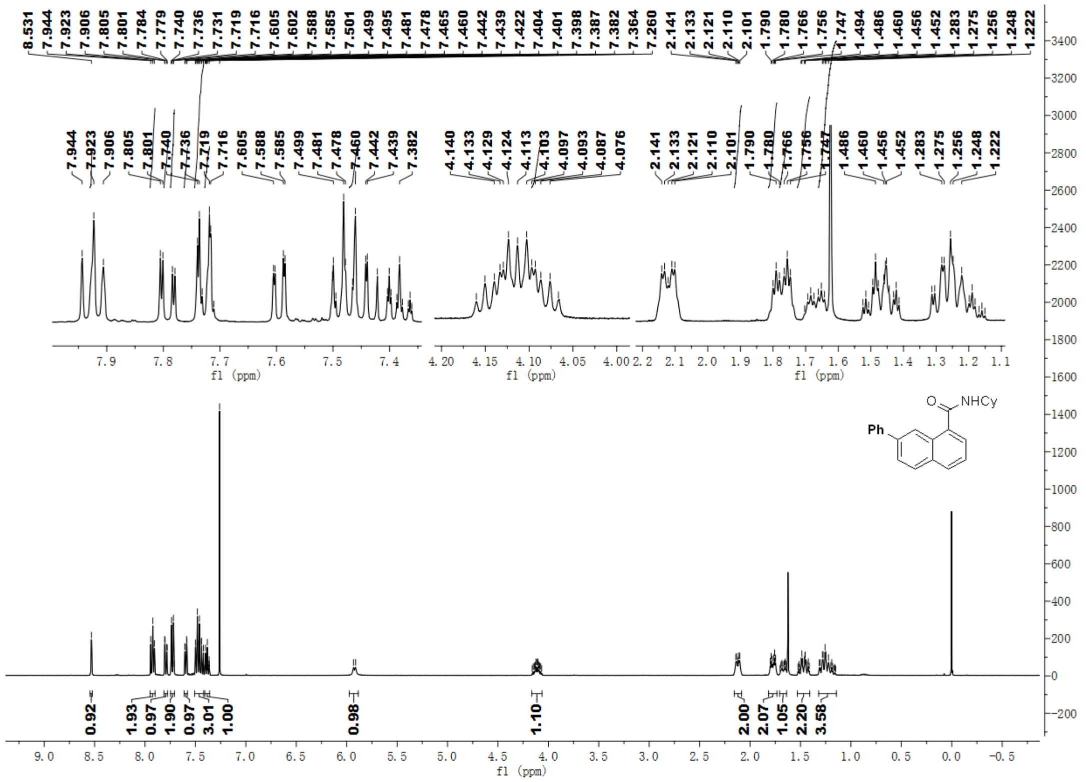

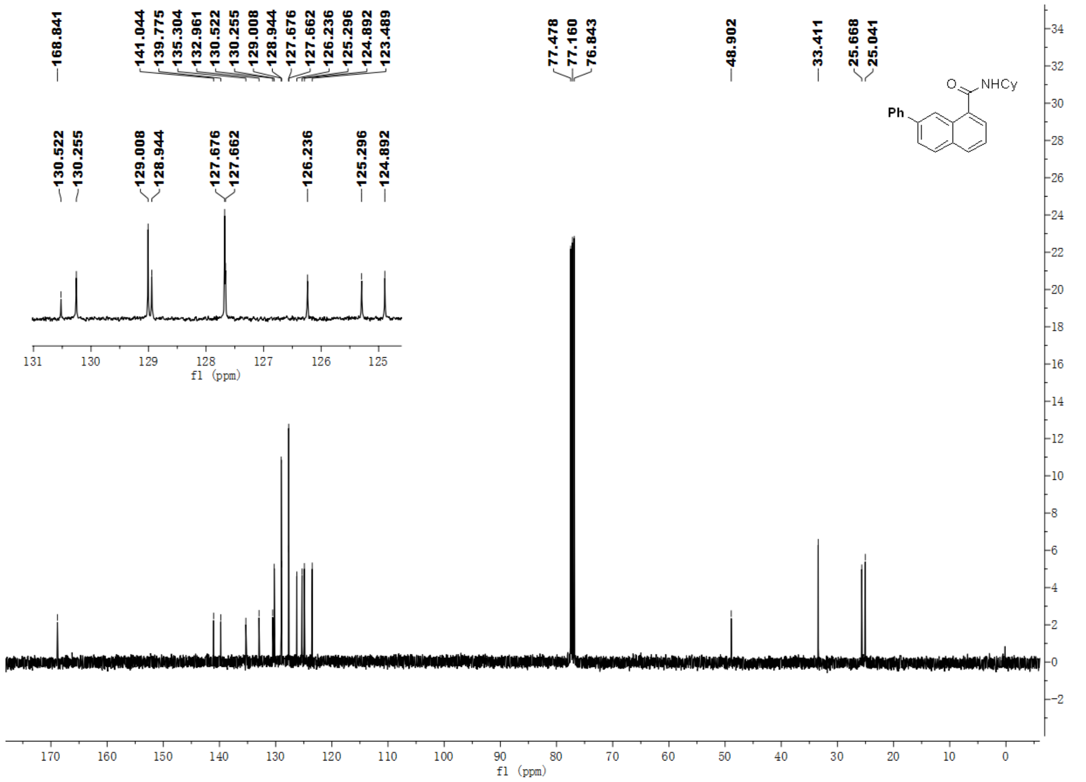

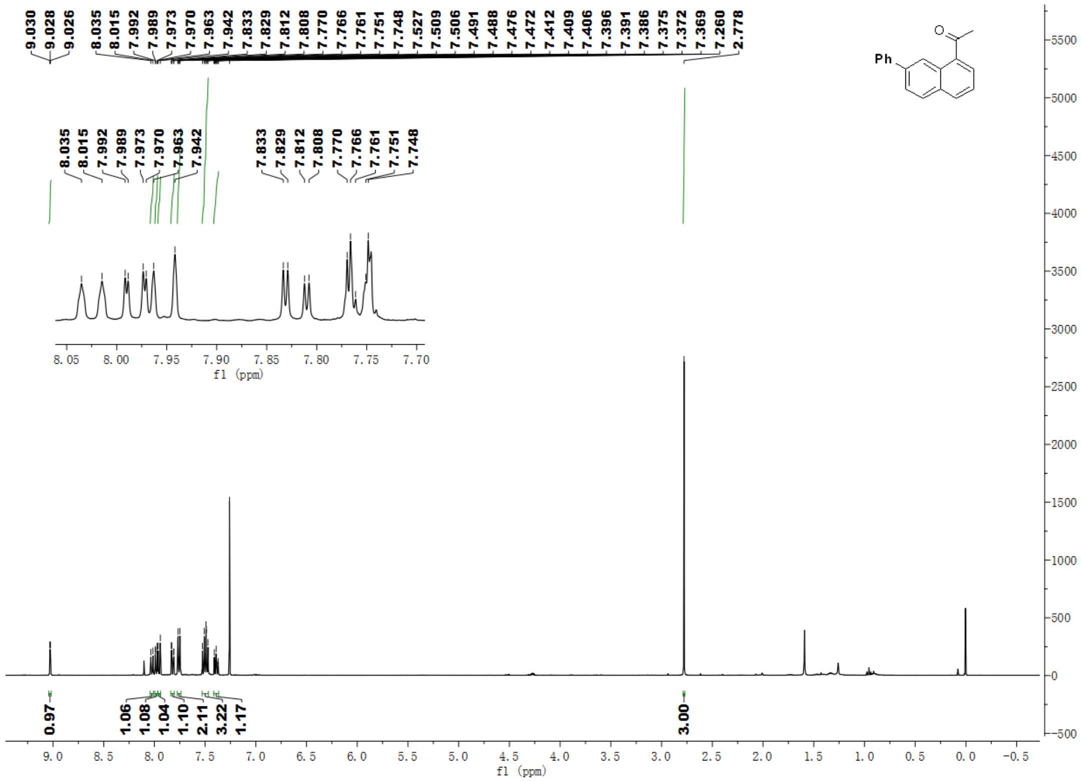

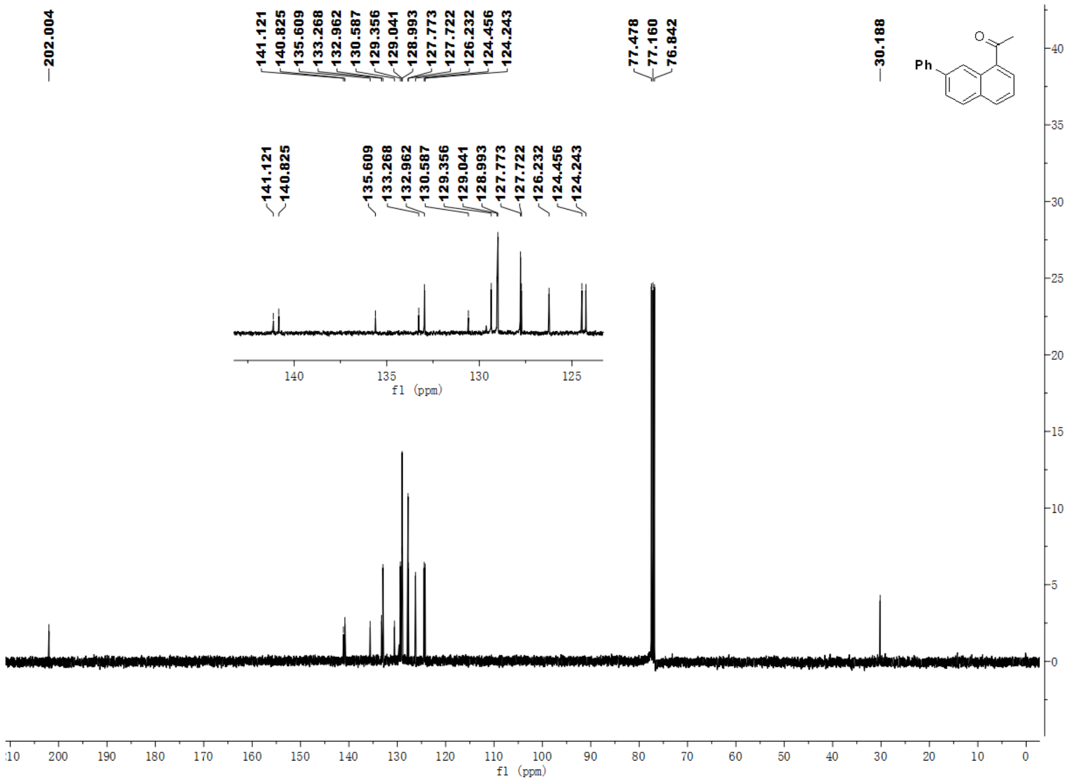

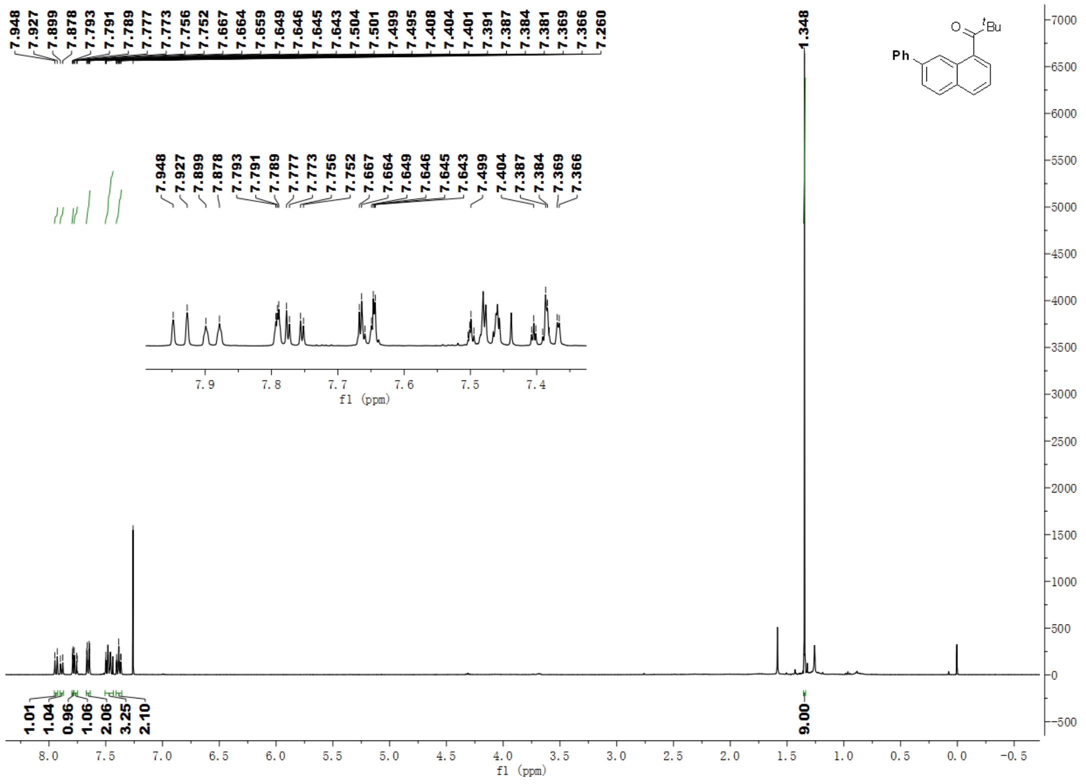
7.948
7.927
7.899
7.878
7.793
7.791
7.789
7.777
7.773
7.756
7.752
7.667
7.664
7.659
7.649
7.646
7.645
7.643
7.504
7.501
7.499
7.495
7.408
7.404
7.401
7.391
7.387
7.384
7.381
7.369
7.366
7.260
1.348
O
tBu
Ph
1.01
1.04
0.96
1.06
2.06
3.25
2.10
9.00
f1 (ppm)

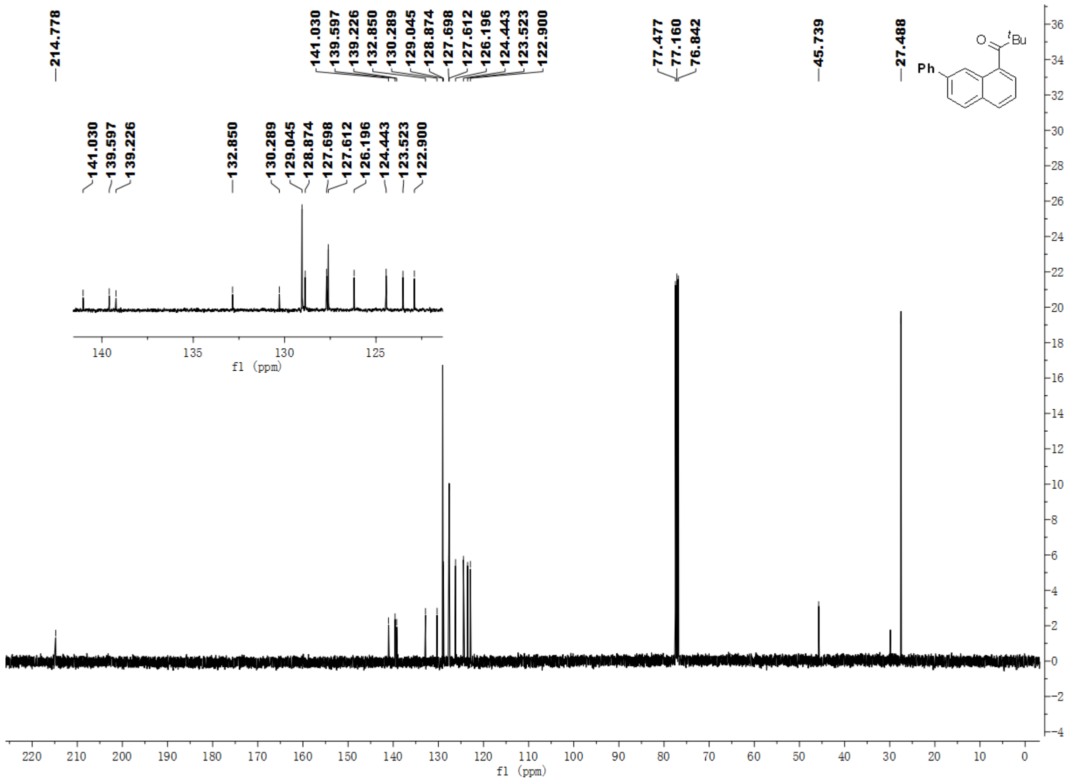
214.778
141.030
139.597
139.226
132.850
130.289
129.045
128.874
127.698
127.612
126.196
124.443
123.523
122.900
77.477
77.160
76.842
45.739
27.488
O
tBu
Ph
f1 (ppm)
